# Conventional laboratory housing increases morbidity and mortality in research rodents: results of a meta-analysis

**DOI:** 10.1186/s12915-021-01184-0

**Published:** 2022-01-13

**Authors:** Jessica Cait, Alissa Cait, R. Wilder Scott, Charlotte B. Winder, Georgia J. Mason

**Affiliations:** 1 Department of Integrative Biology, College of Biological Science, University of Guelph, Guelph, Ontario Canada; 2 Department of Translational Immunology, Malaghan Institute of Medical Research, Wellington, New Zealand; 3 School of Biomedical Engineering, Faculty of Medicine and Applied Science, University of British Columbia, Vancouver, British Columbia Canada; 4 Department of Population Medicine, Ontario Veterinary College, University of Guelph, Guelph, Ontario Canada

**Keywords:** Welfare, Rats, Mice, Health, Mortality, Housing, Enrichment, Standards, External validity, Meta-analysis

## Abstract

**Background:**

Over 120 million mice and rats are used annually in research, conventionally housed in shoebox-sized cages that restrict natural behaviours (e.g. nesting and burrowing). This can reduce physical fitness, impair thermoregulation and reduce welfare (e.g. inducing abnormal stereotypic behaviours). In humans, chronic stress has biological costs, increasing disease risks and potentially shortening life. Using a pre-registered protocol (https://atrium.lib.uoguelph.ca/xmlui/handle/10214/17955), this meta-analysis therefore tested the hypothesis that, compared to rodents in ‘enriched’ housing that better meets their needs, conventional housing increases stress-related morbidity and all-cause mortality.

**Results:**

Comprehensive searches (via Ovid, CABI, Web of Science, Proquest and SCOPUS on May 24 2020) yielded 10,094 publications. Screening for inclusion criteria (published in English, using mice or rats and providing ‘enrichments’ in long-term housing) yielded 214 studies (within 165 articles, using 6495 animals: 59.1% mice; 68.2% male; 31.8% isolation-housed), and data on all-cause mortality plus five experimentally induced stress-sensitive diseases: anxiety, cancer, cardiovascular disease, depression and stroke. The Systematic Review Center for Laboratory animal Experimentation (SYRCLE) tool assessed individual studies’ risks of bias. Random-effects meta-analyses supported the hypothesis: conventional housing significantly exacerbated disease severity with medium to large effect sizes: cancer (SMD = 0.71, 95% CI = 0.54–0.88); cardiovascular disease (SMD = 0.72, 95% CI = 0.35–1.09); stroke (SMD = 0.87, 95% CI = 0.59–1.15); signs of anxiety (SMD = 0.91, 95% CI = 0.56–1.25); signs of depression (SMD = 1.24, 95% CI = 0.98–1.49). It also increased mortality rates (hazard ratio = 1.48, 95% CI = 1.25–1.74; relative median survival = 0.91, 95% CI = 0.89–0.94). Meta-regressions indicated that such housing effects were ubiquitous across species and sexes, but could not identify the most impactful improvements to conventional housing. Data variability (assessed via coefficient of variation) was also not increased by ‘enriched’ housing.

**Conclusions:**

Conventional housing appears sufficiently distressing to compromise rodent health, raising ethical concerns. Results also add to previous work to show that research rodents are typically CRAMPED (cold, rotund, abnormal, male-biased, poorly surviving, enclosed and distressed), raising questions about the validity and generalisability of the data they generate. This research was funded by NSERC, Canada.

**Supplementary Information:**

The online version contains supplementary material available at 10.1186/s12915-021-01184-0.

## Background

Globally, at least 120 million mice and rats are used in biomedical research each year [1–3]. Extensive knowledge has been gleaned from such work, but this has not come without ethical concerns. The vast majority of rodent-based research goes unpublished [4], cannot be replicated [5–8] or fails in translatability [9–11]. Most experimental procedures induce moderate to severe distress or pain [2, 3]. Furthermore, rodents experience chronic impacts from typical laboratory housing; globally, most cages are small and contain little more than food, water and a granular flooring substrate (e.g. corncob). In the wild, in contrast, they dig burrows and create nests as warm, safe resting places, and they explore home ranges that may be several cubic metres (e.g. in buildings), or comprise dozens to hundreds of square metres of field habitat [12, 13]. Laboratory rodents find opportunities to perform these natural behaviours highly rewarding: for example, they prefer cages with nesting material [14, 15], mice building elaborate, well-structured nests if given the right substrates [16]; are motivated to dig burrows [17, 18]; will pay costs in order to exercise, e.g. crossing electrified grids to access running wheels [19] and are motivated to explore novelty [20]. Large cages ‘enriched’ with these opportunities are thus preferred over conventional ones [20] (with mice potentially pushing weights heavier than themselves to reach such environments [21]). Furthermore, conventional cages commonly induce signs of poor welfare that include abnormal behaviours [22–26], cognitive ‘pessimism’ [27–30], impaired sleep quality [31, 32] and reduced resilience to acute stressors [33] (e.g. showing prolonged tachycardia after injection) [34]. Such welfare evidence has accumulated for decades, yet progress towards improving rodent housing has been slow. Since 2010, for example, Europe has required the provision of shelter or nesting for laboratory rodents, to meet one basic biological need [35], Canada following suit for mice in 2019 [36]. However, the USA, likely the number one laboratory rodent user worldwide [1, 37], still does not [38] and nor do many other countries. The use of barren cages thus continues [39]. This is ethically troubling and may have practical implications too: some have proposed that the resulting poor welfare so alters animals’ underlying physiology that they no longer ‘embody healthy biological systems’ [40], such that ‘the applicability of [their] results to the average human, who lives in a stimulating environment, rather than impoverished conditions’ should be questioned [41] (see also [42–45]).

Here we sought evidence for such biological changes. Such evidence is necessary (albeit not sufficient) to support these authors’ hypothesis. Epidemiological research on humans shows the specific types of change that are common under chronic stress. Humans who are chronically stressed have shortened lifespans and are more susceptible to disease [46–48]: the result of stress-induced physiological changes such as supressed immune function [49] and altered hormone signalling [50]. Similar effects can occur in rodents (e.g. rats exposed to ‘chronic mild stress’ procedures show disrupted metabolic profiles [51], and mice experimentally subjected to chronic aggression have shortened lifespans [52]). As the Institute for Laboratory Animal Research (ILAR), the US National Academies body responsible for laboratory animal care guidelines, thus summarizes, ‘animals exposed to prolonged severe stress experience underlying changes in physiological functions (e.g. gastric lesions) or immunosuppression that can … contribute to morbidity and mortality’ ([53]; cited references omitted). This systematic review and meta-analysis therefore aimed to determine if conventional housing has these types of detrimental impacts on rodent health. This hypothesis predicts that compared to ‘enriched’ housing that is more complex and contains resources that support species-typical behaviours, conventional housing will exacerbate disease, especially conditions known to be stress-sensitive, and increase all-cause mortality rates.

## Methods

### Selection of stress-sensitive diseases for morbidity data

There is considerable research on how stress affects disease risk and severity (e.g. a Medline search of ‘psychological stress’ and ‘disease’ generated 1927 hits on May 18, 2020). For feasibility, we therefore first narrowed down a list of relevant diseases by using two extensive reviews on stress and morbidity/mortality as starting points, [54] and [46], hand-searching the titles and abstracts of all references cited, plus papers citing these reviews since publication (found via Google Scholar; see Additional file 1). From these, we selected all diseases described as exacerbated by psychological stress in humans, and mentioned in more than one paper: anxiety disorders, asthma, cancer, cardiovascular disease, major depression, stroke and viral infection. In rodents, these diseases are ‘modelled’ by being induced artificially (thus not always reflecting the natural pathophysiology of disease onset); however, their subsequent severity and duration, and the degree of recovery, all of which are stress-sensitive in humans, are critically dependent on the animal’s physiology (see Additional file 2 for key references).

### Reporting, protocol and registration

A pre-registered review protocol was then deposited in the University of Guelph Atrium (our institutional repository) on May 22, 2020 https://atrium.lib.uoguelph.ca/xmlui/handle/10214/17955; see Additional File 3, plus protocol amendments in Additional file 4). The protocol and this manuscript are both reported in accordance with the Preferred Reporting Items for Systematic Reviews and Meta-Analyses (PRISMA) statement [55] (Additional file 5), and follows the ten appraisal questions for biologists outlined by Nakagawa et al. [56] and practical guidelines for conducting meta-analyses using animal studies [57].

### Disease measures

Eligible studies were required to report a pre-specified disease-relevant outcome (Additional file 2). The review protocol details how these were chosen. Briefly, we generated a shortlist by identifying which signs of each disease are negatively impacted by stress in humans, for cross-reference with those commonly reported in biomedical rodent research (not specific to environmentally ‘enriched’ housing [henceforth EH] literature). For feasibility, we limited our focus to a maximum of three outcomes per disease (though note that each could be measured in multiple ways: see Additional File 2).

### Eligibility criteria

Studies were included if they (i) were published in English; (ii) described primary in vivo research; (iii) used laboratory mice or rats; (iv) used both conventional housing (henceforth CH), and EH, as animals’ long-term living quarters; (v) reported mortality, or used a disease model of interest reporting at least one pre-specified outcome. Eligible studies also required a clear text description or image of the ‘enrichment’ and did not confound conventional housing with isolation (such that differentially housed animals were always in similar social environments [either all individuals, or all paired/grouped]). For studies lacking clear descriptions of the conventional cages, we assumed the minimum housing requirements specified for the relevant year and country (assuming, unless otherwise stated, that researchers would follow both recommended and required minima). Eligible mortality studies had to report a minimum time at risk in weeks or months (shorter endpoints, e.g. within hours or days of disease induction, were considered readouts of specific acute disease models such as models of anorexia, rather than reflecting how stress can increase mortality over a lifetime).

### Data sources and searches

Electronic searches were completed on May 24, 2020, using Medline (via Ovid), CAB abstracts (via CABI), Science Citation Index (via Web of Science), ProQuest Theses & Dissertations (via ProQuest) and Elsevier (via SCOPUS). No restrictions were placed on the search beyond those of the databases themselves. The specific search strategy was created in MEDLINE (OVID interface, 1948 onwards); see Additional file 6 for details. ‘Enrich*’ was used to find relevant housing studies as the typical terminology in such research.

### Article selection

Records from searches were uploaded and de-duplicated in EndNoteX7.8 (Clairvate Analytics, Philadelphia, USA), exported into DistillerSR (Evidence Partners Inc., Ottawa, ON, Canada), further de-duplicated, and then screened in two rounds (title/abstract; full text eligibility) by two independent reviewers (JC and either AC or SL) (see Additional file 7 for screening questions). Prior to screening, a pilot run on the first 100 records for title/abstract, and first 25 records for full text, ensured consistent data collection between reviewers. Any conflicts between reviewers were resolved by consensus.

### Data collection

All data were collected in DistillerSR by two independent reviewers (JC and either AC or SL), conflicts again being resolved by consensus. Study-level data collected on animal, housing, disease and outcome characteristics are shown in Additional file 8.

### Data extraction for housing details and other potential moderating factors

We extracted details of which resources (substrates, items or structures) were included in both housing types. Further, we extracted data on factors likely to compromise EH effectiveness (determined a priori: see pre-registered protocol Additional file 3), hereafter referred to as ‘red flags’: those likely to inadvertently increase aggression (via resource guarding in group-housed male mice) [12, 58, 59], fear (caused by the frequent rotation of novel objects, or providing novel resources to old animals who may be neophobic) [60, 61] or disinterest (possible in old animals, due to anhedonia) [21]. We also flagged any EH supplied for very short timeframes (i.e. less time than the disease could develop in). Meta-regressions were run with and without ‘red flags’ to determine if these factors impacted EH effectiveness (see sections below).

### Data extraction for stress-sensitive diseases

Means, standard deviations (or standard errors) and sample sizes were extracted to calculate and report standardized mean differences (SMD) (Hedge’s G): a unit-less summary statistic used to compare and combine results across studies [62]. A SMD of 0.2, 0.5 or 0.8 was interpreted as a small, medium or large effect respectively [63]. For studies which did not report a specific sample size but gave a range, the smallest possible sample size was used to be conservative. For studies that did not report the mean and standard deviation in the text, we extracted values from graphs using Web Plot Digitizer [64]. Studies that did not report how error bars were generated were excluded. For studies reporting multiple experimental groups or time points, we excluded loss of function and gain of function (within-subject) experiments, and if data were sampled at multiple time points, we only extracted data from the latest reported time point (prior to full recovery from disease). For studies generating more than one SMD, to avoid pseudoreplication only one was kept when analyses were pooled (always the least studied measure across all articles).

### Data extraction for mortality

To assess all-cause mortality, we computed hazard ratios and median survivals by reconstructing Kaplan-Meier curves from curves presented in articles using Web Plot Digitizer [64]. We extracted data in duplicate and compared extracted coordinates for concordance. Any discrepancies between the two reviewers (JC and SL) were resolved by re-extracting coordinates until concordant; one reviewer’s data (JC) were then used for final Kaplan-Meier curve construction, and to calculate hazard ratios (a summary of time-to-event data, which here measures relative instantaneous risk of death between CH and EH populations [62]), confidence intervals and median survival times via methods and R script from Guyot et al. [65].

### Risk of experimental bias

Risks of bias in individual studies were assessed independently by two separate reviewers (JC and SL) using the SYstematic Review Center for Laboratory animal Experimentation (SYRCLE) risk of bias tool [66], disagreements again resolved by consensus.

### Data synthesis and meta-analysis of housing effects

Random-effects meta-analyses were conducted in R 3.6.2 (R Foundation for Statistical Computing, Vienna, Austria) using the random-effects meta-analysis function (rma) in the Metafor package [67]. Each study was weighted by its inverse variance (with secondary analyses *without* study weights also being performed, to avoid any skews from unit-of-analysis errors [see ‘Results’]; these can be found in Additional files 9, 10, 11). For experiments in which only one comparator group was used in a multi-arm study, we increased the assumed variance to avoid unit-of-analysis errors, based on Rücker et al.’s ‘Method Three’ [68]. Each SMD (Hedge’s G) was reported such that a value greater than zero indicated increased morbidity in conventional cages. Hazard ratio data were analysed by imputing log hazard ratios, and reported so that a hazard ratio > 1 represents increased mortality in conventional cages. Median survival times were analysed using the log transformed ratio of means (ROM) and reported so that a ROM < 1 represents reduced median survival in conventional cages [69]. A separate meta-analysis was performed for each stress-sensitive disease. All disease data were then pooled for subsequent analysis of moderator effects (see below). Hazard ratio data were also run through these analyses (see below). However, median survivals were not, since we could only generate this metric for a few studies (those reaching a minimum 50% survival and recording enough deaths afterwards to calculate 95% confidence intervals [CI]).

All R code used can be found in Additional file 12. Studies meeting eligibility criteria but not included in the meta-analysis are summarized in Additional file 13.

### Exploring heterogeneity

Heterogeneity was assessed with the *I*^2^ statistic [67]. For each stress-sensitive disease, data were split into subgroups by measure. For mortality, data were split into subgroups according to whether or not an experimental disease had been induced (i.e. whether animals were being used to model a disease or were instead expected to be healthy). Differences between subgroup effect estimates were analysed statistically by including ‘measure’ as a moderator in the random-effects model. Potential moderators of housing effects (e.g. species, sex; see below) were then explored, using meta-regressions on pooled stress-sensitive disease data and hazard ratio data separately.

### Assessing publication bias

Before this exploration of housing effect moderators, we first assessed evidence for publication bias across studies, specifically selective reporting (e.g. omission of non-significant findings from small studies [70]), both statistically (via rank correlation tests [71]), and by examining the degree of effect asymmetry visually in funnel plots (c.f [72, 73].). Funnel plots were generated for all stress-sensitive disease studies (pooled). Any extreme SMD values (≥ 3) not ‘reflected’ in the plot (indicating the likely non-publication of small studies not rejecting the null hypothesis, thus a publication bias) were removed before further analysis (to be conservative, and also to achieve normal residuals). This process was repeated for studies reporting mortality data (extracted as hazard ratios), revealing no such biases.

### Do housing effects vary with species, sex or disease?

Next, we explored whether specific diseases or groups of animals impacted the SMD, pooling all disease data for analysis, residuals first being checked for normality (Shapiro-Wilk test). We included the following moderators via the ‘mods’ argument: disease (cancer, stroke, depression, anxiety or cardiovascular disease); species (mice or rats); social housing status (individually housed, socially housed or not specified); and sex (male, female or other [mixed or not specified]) as categorical variables, as well as their two-way interactions. Since infarct volume SMDs significantly differed from other measures, based on the preceding analyses comparing subgroups by measure, we also incorporated this as a binary variable (yes this study measured infarct volume / no it did not). Hazard ratio data were then similarly analysed, but instead of including ‘disease model’ as a moderator, we included whether or not any type of experimental disease was induced.

### Do housing effects vary with the number and type of resources supplied?

Finally, to identify key mediators of any differences between CH and EH, we assessed the impact of the type of differentially supplied resource. ‘Resource type’ was scored based on a priori determination of factors likely to reduce health and welfare in CH/improve it in EH (see Additional file 14). These were as follows: wheels, which are highly motivating and can reduce obesity and hyperinsulinemia (especially in rats) [74–78]; nesting opportunities, which are also highly motivating, and enable proper thermoregulation (especially in mice) [79–83]; and opportunities to perform other natural activities (e.g. gnawing, exploration). Based on these, across all disease studies the differences in resources between CH and EH fell into four well-represented categories: studies where EH provided wheels only (*n* = 78), opportunities for other activities (but no wheel or nesting; *n* = 18), both wheels and opportunities for other activities (but no nesting; *n* = 40), and all three resource types (*n* = 24). For hazard ratio data, studies fell into just two well-sampled categories: ones where EH provided a wheel only (*n* = 23) and a heterogeneous group in which EH provided several resources unavailable in CH (*n* = 11). ‘Resource category’ and its interaction with species was then added to each model. Both models were then rerun with ‘red flag’ studies removed.

### Coefficient of variation

To test whether housing condition alters the amount of variability seen in experimental outcomes, we conducted a meta-analysis on the log transformed ratios of the coefficient of variation (CVR) using a random-effects model (as described above) [84].

### Figures

Figures were generated using the Metafor package [67] and stylized using Adobe Illustrator CC (Adobe), except for the risk of bias of individual studies which was generated using GraphPad Prism v7.05 (GraphPad Software, San Diego, California USA).

### Confidence in cumulative evidence

The strength of the body of evidence synthesized in this review was assessed (by JC) using the Grading of Recommendations Assessment, Development and Evaluation (GRADE) guidelines [85]. Briefly, the quality of evidence was assessed based on study design (as high, moderate, low or very low) and reduced if there was a high risk of bias, imprecision, inconsistency or indirectness and increased if there was a large effect.

## Results

### Study selection and characteristics

**Fig. 1.**
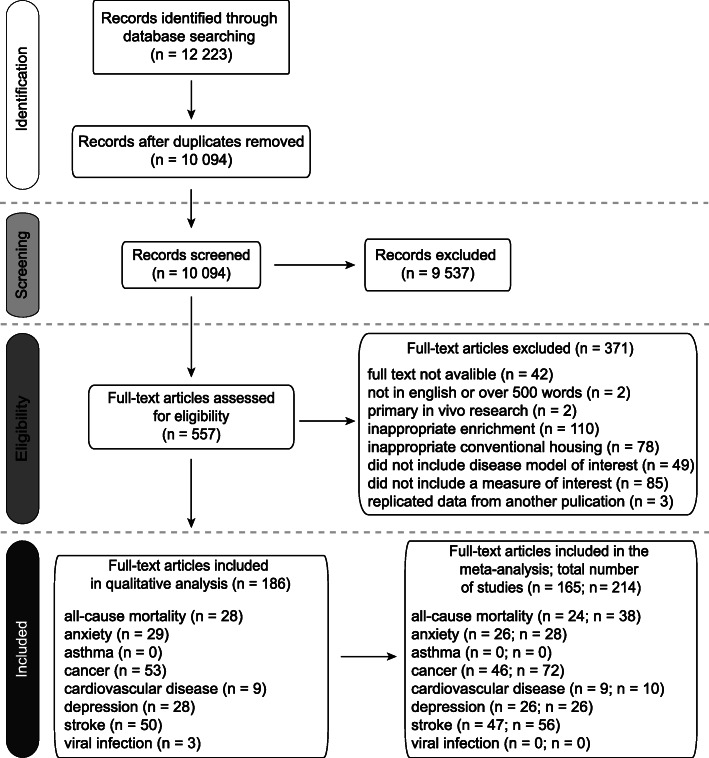
Preferred Reporting Items for Systematic Reviews and Meta-Analyses (PRISMA) flow diagram. Article = one published unit, Study = one group of animals (one article may contain multiple studies)

Search strategy and study selection results are presented in Fig. 1. After de-duplication, 10,094 titles/abstracts were screened, with 9537 being excluded. The full texts of the remaining 557 were then screened, 371 not meeting eligibility criteria, three of which were excluded due to suspected plagiarism (see Additional file 13). This left 186 articles for qualitative synthesis (see Additional file 15; for full reference list see Additional file 16), of which 165 (containing 214 studies using 6495 animals) were included in the meta-analysis (with only one SMD from each study being included in pooled analyses). Of these 214 studies, 59.8% used mice (40.3% rats) and 62.6% used males only while 29.0% used females only (plus 5.6% used both sexes and 2.8% did not specify), and 31.8% housed animals individually (57.0% having > 1 animal per cage, but 11.2% not specifying social housing status at all). 66.8% of studies did not adequately describe their CH, leaving us to infer it from local minimum standards.

**Fig. 2:**
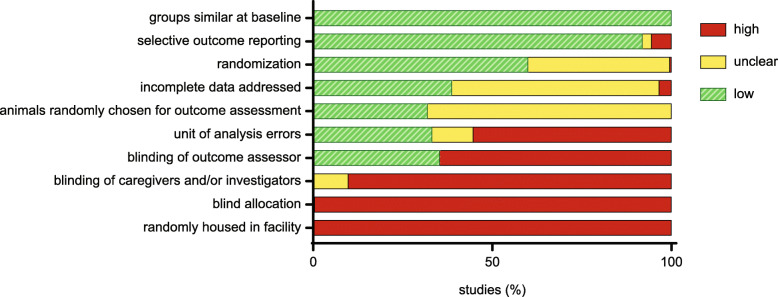
Graphical representation of the results from the SYRCLE risk of bias tool. Green indicates low risk of bias, yellow unclear and red indicates high risk of bias

Assessments of experimental risk of bias can be seen in Additional file 17 and Fig. 2. Notably, 60.0% of studies reported randomization of animals to treatment groups, but only two studies indicated *how* they randomized (a required element of the ARRIVE guidelines [86]), and only 35.3% of studies indicated blinding of outcome assessors. Furthermore, many studies (55.3% of those using socially housed animals) did not use the correct unit of statistical analysis for research like this (which manipulates housing at the cage level), namely ‘cage’, rather than ‘animal’ (cf. [87–90]). No other sources of bias were observed.

### Asthma and viral infection

No studies of asthma and only three of viral infection met inclusion criteria for this review, with only one viral infection study reporting an outcome extractable for meta-analysis. A meta-analysis of viral infection data was therefore not performed. The other five diseases are presented below in order of increasing housing-type effect size.

### Cancer

**Fig. 3:**
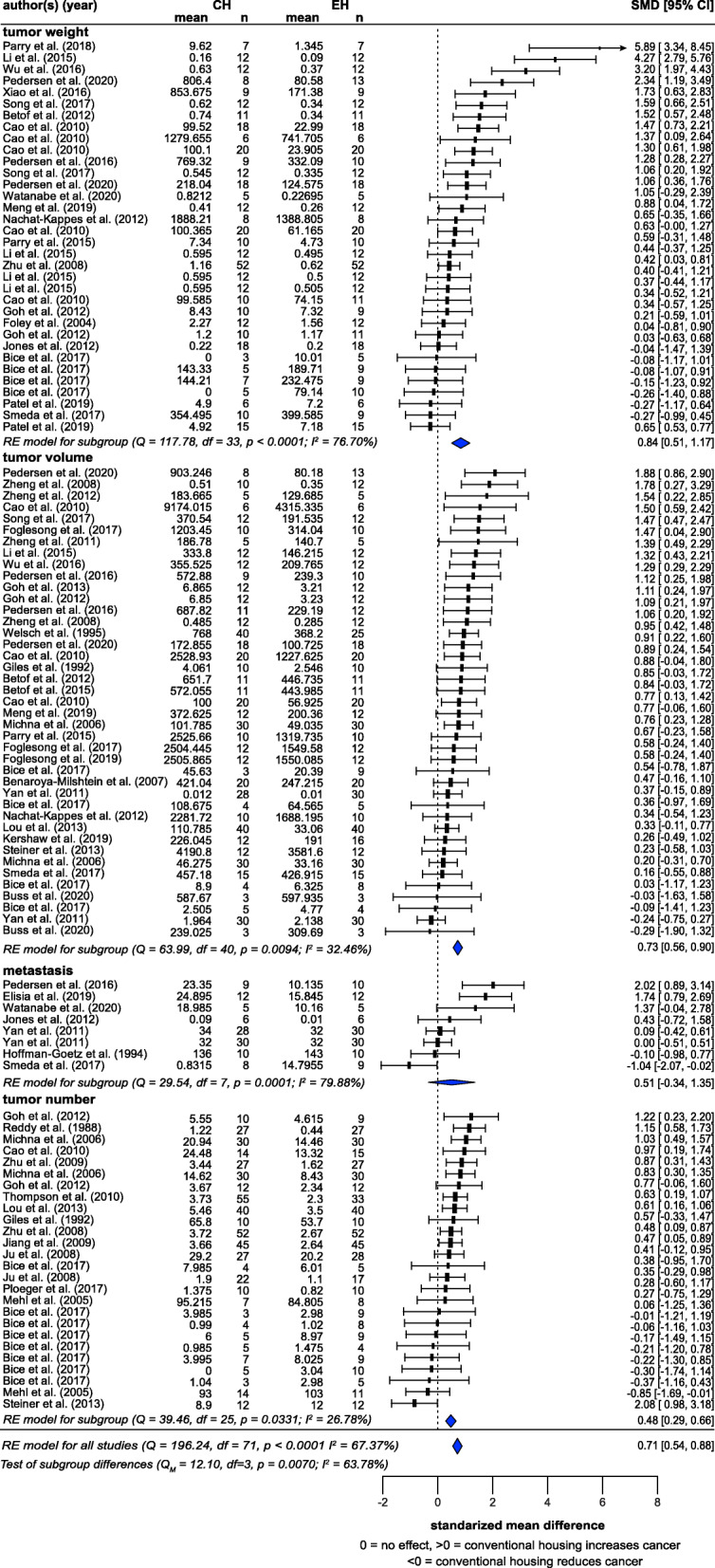
Random-effects meta-analysis of housing effects showing overall standardized mean difference (SMD [Hedge’s G]) and 95% confidence intervals of cancer studies. Subgroups are based on measures. Blue diamond = SMD estimate (with the width reflecting the 95% CI), location of black squares indicates study SMD and size indicates the weight of the study in the meta-analysis. CH = conventional housing, EH = ‘enriched’ housing, RE = random effects. *I*^2^ and Q statistic are tests of heterogeneity. Results of the model testing whether SMDs are significantly different from zero: *n* = 72, *z* = 8.29, *p* < 0.0001

Meta-analysis of 72 studies (from 46 articles) showed that CH significantly exacerbated cancer morbidity, with a medium effect size (SMD = 0.71, *z* = 8.29, *p* < 0.0001) (Fig. 3). There was, however, a substantial amount of heterogeneity (*I*^2^ = 67.37%). Subgroup analyses by measure showed that tumor number (26 studies; SMD = 0.48, *z* = 4.21, *p* < 0.0001), tumor volume (41 studies; SMD = 0.73, *z* = 0.08, *p* < 0.0001) and tumor weight (34 studies; SMD = 0.84, *z* = 5.14, *p* < 0.0001) all showed significant housing effects. Metastatic measures (8 studies) did not reach statistical significance, likely due to the small *N*, although the effect was consistent with the hypothesis (SMD = 0.51, *z* = 1.14, *p* = 0.2000). Subgroups were very consistent: they did not significantly differ (*p* = 0.1764), and controlling for ‘measure’ did not decrease *I*^2^, although some were less heterogeneous than others (tumor number [*I*^2^ = 26.78%], tumor volume [*I*^2^ = 32.46%], tumor weight [*I*^2^ = 76.70%] and metastasis [*I*^2^ = 79.88%]).

### Cardiovascular disease

**Fig. 4:**
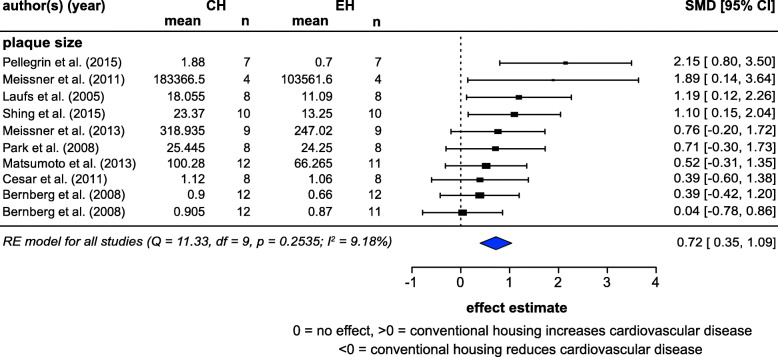
Random-effects meta-analysis of housing effects showing  overall standardized mean difference (SMD [Hedge’s G]) and 95% confidence intervals of cardiovascular disease studies. Blue diamond = SMD estimate (with the width reflecting the 95% CI), location of black squares indicates study SMD and size indicates the weight of the study in the meta-analysis. CH = conventional housing, EH = ‘enriched’ housing, RE = random effects. *I*^2^ and Q statistic are tests of heterogeneity. Results of the model testing whether SMDs are significantly different from zero: *n* = 10, *z* = 4.36, *p* = 0.0018

Ten studies (from nine articles) reported atherosclerotic plaque size. Meta-analysis showed a medium to large effect size, with CH significantly exacerbating plaque magnitude (SMD = 0.72, *z* = 4.36, *p* = 0.0018) (Fig. 4). This analysis had low heterogeneity (*I*^2^ = 9.18%).

### Stroke

**Fig. 5:**
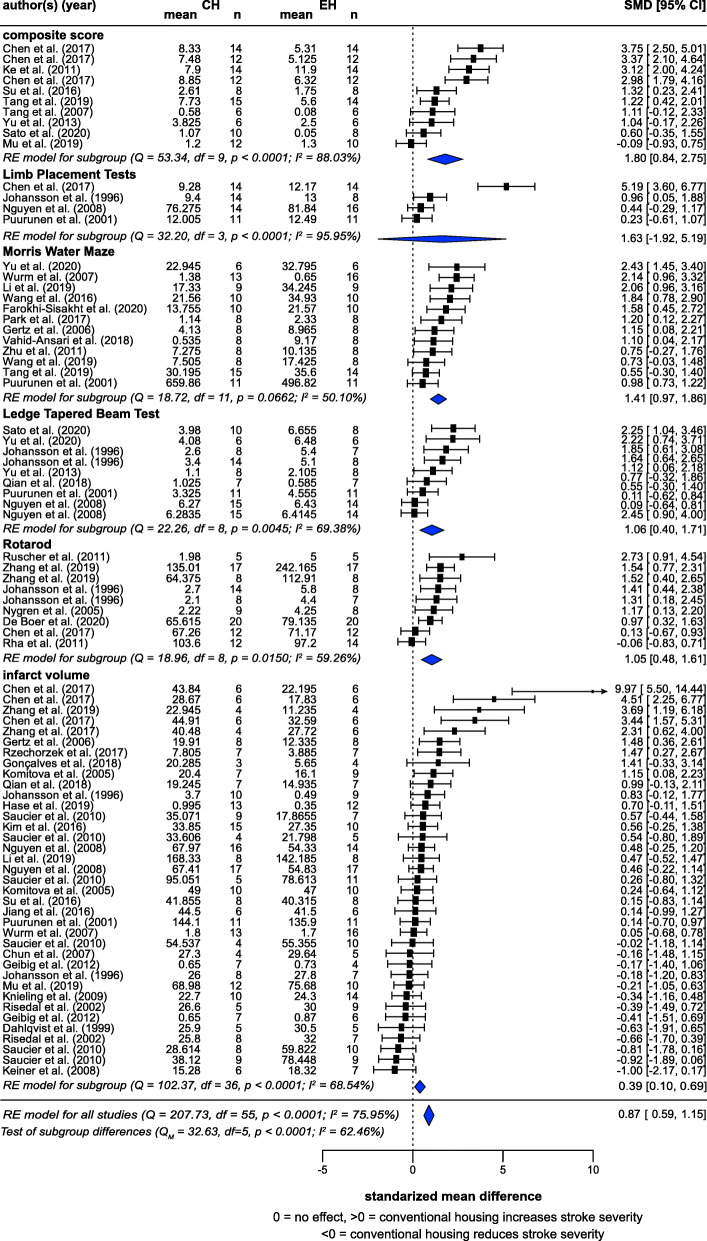
Random-effects meta-analysis of housing effects showing overall standardized mean difference (SMD [Hedge’s G]) and 95% confidence intervals of stroke studies. Subgroups  are based on measures. Blue diamond = SMD estimate (with the width reflecting the 95% CI), location of black squares indicates study SMD and size indicates the weight of the study in the meta-analysis. CH = conventional housing, EH = ‘enriched’ housing, RE = random effects. *I*^2^ and Q statistic are tests of heterogeneity.  Results of the model testing whether SMDs are significantly different from zero: *n* = 56, *z* = 6.11, *p* < 0.0001

Meta-analysis of 56 studies (from 47 articles) showed that CH significantly exacerbated the outcomes of induced stroke, with a large effect size (SMD = 0.87, *z* = 6.11, *p* < 0.0001) (Fig. 5). A substantial amount of heterogeneity was observed (*I*^2^ = 75.95%). Subgroup analyses by measure were consistent; composite score (10 studies): SMD = 1.80, *z* = 4.26, *p* = 0.0021; Morris Water Maze (12 studies): SMD = 1.41, *z* = 7.03, *p* < 0.0001; Ledge Tapered Beam Test (9 studies): SMD = 1.06, *z* = 3.71, *p* = 0.0059; Rotarod (9 studies): SMD = 1.05, *z* = 4.3, *p* = 0.0026; and infarct volume (37 studies): SMD = 0.39, *z* = 2.73, *p* = 0.0098). Effects in Limb Placement Tests (4 studies) did not reach statistical significance, likely due to the small *N*, although the effect was consistent with the hypothesis (SMD = 1.63, *z* = 1.45 *p* = 0.2420). A significant difference between measures (*p* = 0.0023) reflected a lower SMD for infarct volume, and so controlling for ‘measure’ reduced heterogeneity (*I*^2^ = 62.46%).

### Anxiety

**Fig. 6:**
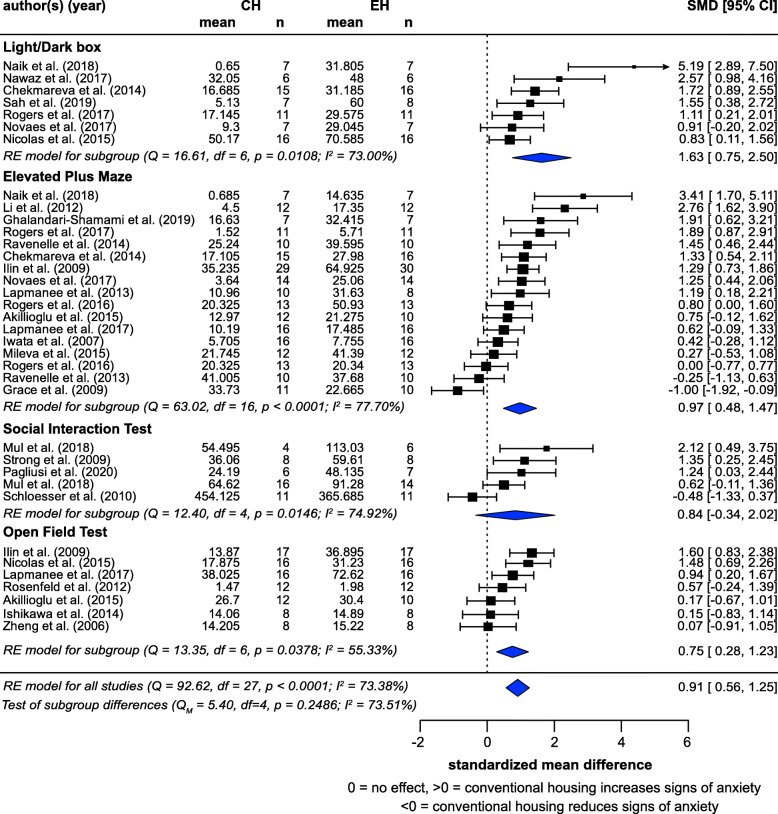
Random-effects meta-analysis of housing effects showing overall standardized mean difference (SMD [Hedge’s G]) and 95% confidence intervals of signs of anxiety. Subgroups are based on measures. Blue diamond = SMD estimate (with the width reflecting the 95% CI), location of black squares indicates study SMD and size indicates the weight of the study in the meta-analysis. CH = conventional housing, EH = ‘enriched’ housing, RE = random effects. *I*^2^ and Q statistic are tests of heterogeneity. Results of the model testing whether SMDs are significantly different from zero: *n* = 28, *z* = 5.14, *p* < 0.0001

Meta-analysis of 28 studies (from 26 articles) showed that CH significantly exacerbated signs of anxiety, with a large effect size (SMD = 0.91, *z* = 5.14, *p* < 0.0001) (Fig. 6). There was a substantial amount of heterogeneity (*I*^2^ = 73.38%). Subgroup analyses by measure were generally consistent: Light/Dark box (7 studies): SMD = 1.63, *z* = 4.55, *p* = 0.0038; Elevated Plus Maze (17 studies): SMD = 0.97, *z* = 4.2, *p* = 0.0007; and Open Field Tests (7 studies): SMD = 0.75, *z* = 3.12, *p* = 0.0018. Effect estimates in Social Interaction Tests (5 studies) did not reach significance, likely due to the small number of studies, but the effect was consistent with the other measures (SMD = 0.84, *z* = 1.197, *p* = 0.1200). No significant difference between these measures was observed (*p* = 0.1843), and controlling for ‘measure’ did not decrease *I*^2^.

### Depression

**Fig. 7:**
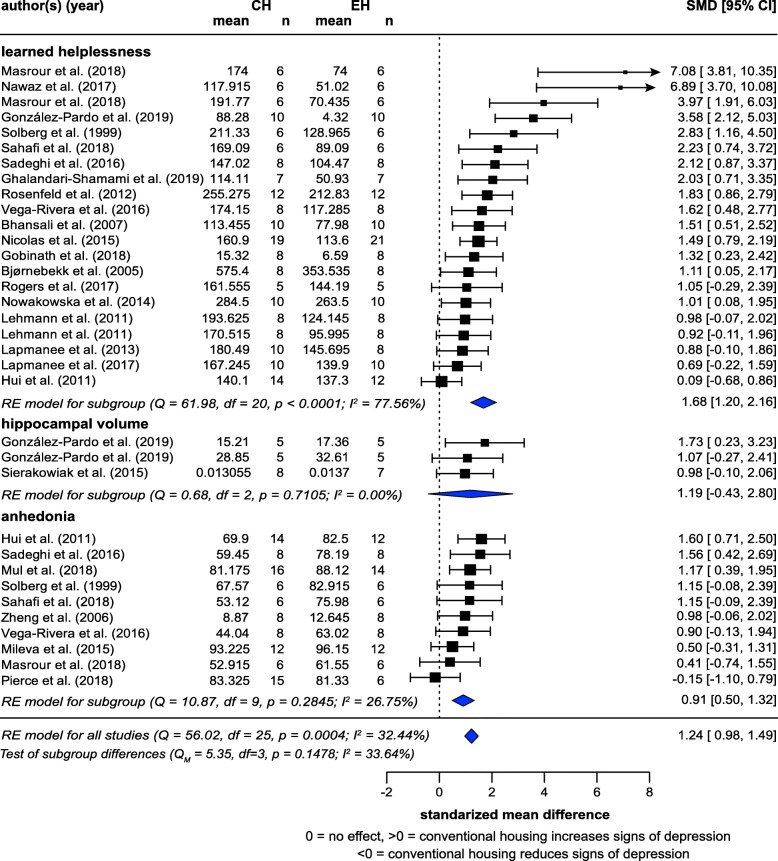
Random-effects meta-analysis of housing effects showing overall standardized mean difference (SMD [Hedge’s G]) and 95% confidence intervals of signs of depression. Subgroups are based on measures. Blue diamond = SMD estimate (with the width reflecting the 95% CI), location of black squares indicates study SMD and size indicates the weight of the study in the meta-analysis. CH = conventional housing, EH = ‘enriched’ housing, RE = random effects. *I*^2^ and Q statistic are tests of heterogeneity. Results of the model testing whether SMDs are significantly different from zero: *n* = 26, *z* = 9.39, *p* < 0.0001

Meta-analysis of 26 studies (26 articles) showed that CH exacerbated signs of induced depression, with a very large effect (SMD = 1.24, *z* = 9.39, *p* < 0.0001) (Fig. 7). Heterogeneity was low (*I*^2^ = 32.44%). When subgroup analysis was performed for each measure, learned helplessness (21 studies; SMD = 1.74, *z* = 7.06, *p* < 0.0001) and anhedonia (10 studies; SMD = 0.911, *z* = 4.99, *p* = 0.0007) showed significant effects. Effects for hippocampal volume (3 studies) did not reach significance, again likely due to the small number of studies, although the effect direction was consistent with the hypothesis (SMD = 1.19, *z* = 3.17, *p* = 0.0870). There was no significant difference between subgroups (*p* = 0.1670).

### Hazard ratios

**Fig. 8:**
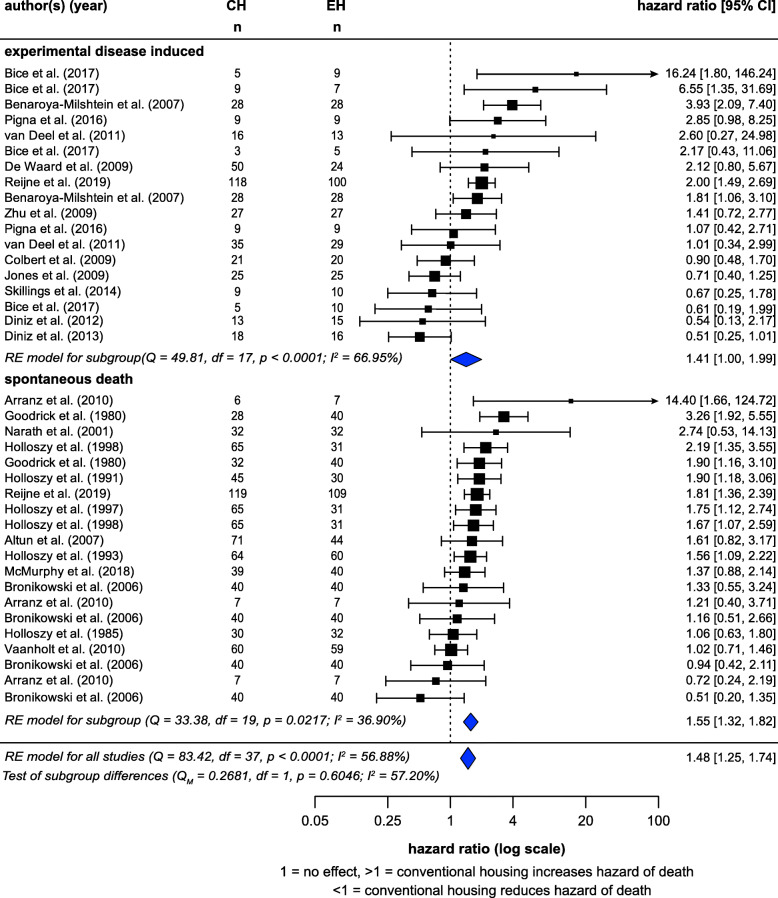
Random-effects meta-analysis of housing effects showing overall hazard ratios and 95% confidence intervals. Subgroups are based on if animals died spontaneously or after disease induction. Blue diamond = SMD estimate (with the width reflecting the 95% CI), location of black squares indicates study hazard ratio and size indicates the weight of the study in the meta-analysis. CH = conventional housing, EH = ‘enriched’ housing, RE = random effects. *I*^2^ and Q statistic are tests of heterogeneity. Results of the model testing whether hazard ratios are significantly different from one:  *n* = 38, *z* = 8.87, *p* < 0.0001

A random-effects meta-analysis of the hazard ratios calculated for 38 studies (from 24 articles) showed a significant effect of housing (CH:EH hazard ratio = 1.48, *z* = 8.87, *p* < 0.0001) (Fig. 8), CH thus increased risk of death at any time point by 48%. There was a substantial amount of heterogeneity (*I*^2^ = 56.88%). Effects were similar in the subset where death occurred with no prior disease induction (hazard ratio = 1.55, *z* = 5.36, *p* < 0.0001) and the subset in which there was prior disease induction (any disease, not just those mentioned previously) (hazard ratio = 1.41, *z* = 1.97, *p* = 0.0486). These subgroups did not differ (*p* = 0.6046), and controlling for subgroup did not decrease *I*^2^.

### Median survivals

**Fig. 9:**
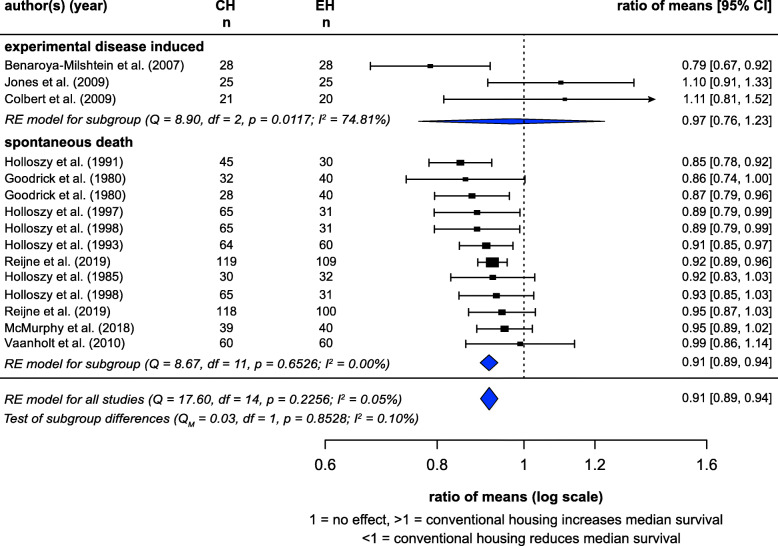
Random-effects meta-analysis of housing effects showing overall ratio of means (ROM) and 95% confidence intervals of median survival. Subgroups are based on if animals died spontaneously or after disease induction. Blue diamond = SMD estimate (with the width reflecting the 95% CI), location of black squares indicates study ratio of means and size indicates the weight of the study in the meta-analysis. CH = conventional housing, EH = ‘enriched’ housing, RE = random effects. *I*^2^ and Q statistic are tests of heterogeneity. Results of the model testing whether ROMs are significantly different from one: *n* = 15, *z* = − 7.04, *p* < 0.0001

A random-effects meta-analysis of median survival times calculated for 15 studies (from 12 articles) showed a significant effect of housing (ROM = 0.91, *z* = − 7.89, *p* < 0.0001) (Fig. 9). Mice and rats in CH had 8.55% lower median survival times than EH counterparts. There was a very low level of heterogeneity (*I*^2^ = 0.05%). For 12/15 studies, the deaths occurred with no prior disease induction, and effects were similar looking only at this subgroup (ROM = 0.91, *z* = − 7.78, *p* < 0.0001). In the other three studies, which did involve disease induction, effects did not reach significance, although the effect direction was consistent with the hypothesis (ROM = 0.97, *z* = − 0.26, *p* = 0.796). Subgroups did not differ in ROMs (*p* = 0.8528).

### Do housing effects on stress-related disease vary with species, sex and disease?

Visual inspection of the funnel plot (Additional file 18A), and a rank correlation test indicated the presence of potential publication bias (tau = 0.253, *p* < 0.001). Much of this skew came from a subset of 9 relatively small-scale studies reporting very large SMDs (> 3 up to over 7). To be conservative, and also to achieve normal residuals, we removed these studies for all subsequent analyses; Additional file 18B shows the revised funnel plot. A random-effects meta-regression then assessed whether animal or disease characteristics predicted the effects of housing. SMD magnitudes were not predicted by species, sex, social housing or their interactions (see Table 1), nor were they affected by disease when ‘red flags’ were included, though housing had smaller effects on infarct volume than on all other measures (see Table 1). Adding these moderators did reduce overall heterogeneity, however (*I*^2^ = 54.91%). After removing ‘red flag’ studies, there was a significant effect of disease, driven by larger effect sizes of housing on stroke outcomes (*p* = 0.0235).

**Table 1:** Meta-regression of potential moderators of housing effects on stress-sensitive disease

	Test statistic	***p***
**Species**	F_1,154_ = 0.2222	0.6381
**Sex**	F_2,154_ = 0.0160	0.9841
**Social status**	F_2,154_ = 0.6564	0.5201
**Disease**	F_4,154_ = 1.3522	0.2532
**Measure: infarct volume versus others**	F_1,154_ = 22.7385	**< 0.0001**
**Species × sex**	F_2,154_ = 1.6491	0.1956
**Species × social status**	F_2,154_ = 0.5794	0.5615
**Sex × social status**	F_4,154_ = 0.6208	0.6484
**Resource category**	F_3,139_ = 0.8280	0.4806
**Resource category × species**	F_3,139_ = 1.0409	0.3766
**After removal of ‘red flags’**		
**Species**	F_1,91_ = 0.0351	0.8517
**Sex**	F_2,91_ = 0.2542	0.7761
**Social status**	F_2,91_ = 0.4339	0.6493
**Disease**	F_4,91_ = 2.5952	**0.0415**
**Measure: infarct volume versus others**	F_1,91_ = 15.8439	**0.0001**
**Species × sex**	F_2,91_ = 0.9884	0.3761
**Species × social status**	F_2,91_ = 0.9929	0.3745
**Sex × social status**	F_4,91_ = 0.2229	0.9250
**Resource category**	F_3,83_ = 2.5128	0.0641
**Resource category × species**	F_3,83_ = 0.8890	0.4504

Results from a random-effects meta-regression investigating potential moderators of housing effects (effects of conventional housing versus housing ‘enriched’ with resources supporting species-typical behaviour) on stress-sensitive disease (standardized mean differences). (See Additional file 10 for a replicate excluding study weights). Bold *p* values are significant at *p* < 0.05.

### Do housing effects on mortality vary with species and sex?

Visual inspection of the funnel plot (Additional file 19) and rank correlation test indicated no publication bias (tau = 0.007, *p* = 0.9500); all studies were therefore retained for subsequent analyses (in which residuals were approximately normal). Hazard ratio magnitudes were not predicted by species, sex, social housing status or their interactions (Table 2). However, adding these moderators did reduce overall heterogeneity (*I*^2^ = 45.71%).

**Table 2:** Meta-regression of potential moderators of housing effects on hazard ratios

	Test statistic	***p***
**Species**	F_1,27_ = 0.3206	0.5759
**Sex**	F_1,27_ = 0.1448	0.8659
**Social status**	F_2,27_ = 0.8277	0.4479
**Spontaneous or induced disease**	F_1,27_ = 0.0141	0.9065
**Species × sex**	F_1,27_ = 0.0003	0.9854
**Species × social status**	F_1,27_ = 0.0120	0.9136
**Sex × social status**	F_2,27_ = 0.6782	0.5160
**Resource category**	F_1,26_ = 0.0025	0.9608
**After removal of ‘red flags’**		
**Species**	F_1,18_ = 0.5480	0.4687
**Sex**	F_2,18_ = 0.0773	0.9259
**Social status**	F_2,18_ = 0.1923	0.8267
**Spontaneous or induced disease**	F_1,18_ = 0.8882	0.3584
**Species × sex**	F_1,18_ = 0.1946	0.6643
**Species × social status**	F_1,18_ = 1.6277	0.2182
**Sex × social status**	F_2,18_ = 0.5397	0.5921
**Resource category**	F_1,17_ = 0.0595	0.8103

Results from a random-effects meta-regression investigating potential moderators of housing effects (effects of conventional housing versus housing ‘enriched’ with resources supporting species-typical behaviour) on mortality rates (hazard ratios). (See Additional file 11 for a replicate excluding study weights)

### Do housing effects vary with the number and type of resources supplied?

For stress-related diseases, SMDs were not predicted by ‘resource category’ (F_3,139_ = 0.8280, *p* = 0.4806) nor its interaction with species (F_3,139_ = 1.0409, *p* = 0.3766) (Table 1), although adding it did modestly reduce the *I*^2^ statistic (*I*^2^ = 45.24%). Removing ‘red flag’ studies had little effect on these null results (Table 1). A trend appeared for ‘resource category’, but this made little biological sense (effects were paradoxically smallest when *all* resources were supplied and largest when only wheels and other resources were supplied [but nesting was absent]), and it also vanished when study weightings were removed (see below).

Turning to mortality, hazard ratios were also not predicted by ‘resource category’ (F_1,26_ = 0.0025, *p* = 0.9608). Almost all (11/12) studies providing multiple items (not just wheels) used mice, so we were unable to assess interactions between resource category and species. Adding the ‘resource category’ term did not reduce the *I*^2^ statistic (*I*^2^ = 48.32%), and again removing ‘red flag’ studies had negligible effect on these null results (Table 2).

### Random-effects meta-analyses with study weights removed

All random-effects models were then rerun omitting the weighting for study variance, because variance had been calculated using the *N* provided by each study, which in many cases were pseudoreplicative (see above). Results changed little; however, this conservative approach did slightly increase the effect sizes (please see Additional file 9, 10, 11).

### Coefficient of variation

We assessed whether the coefficient of variation differed between conventional housing and enriched conditions in stress-related diseases. There was no significant effect of housing on the coefficient of variation (CVR = 0.03, *z* = 0.93, *p* = 0.3520).

### Confidence in cumulative evidence

Assessment of the strength of evidence evaluated using GRADE guidelines indicated high quality (Additional file 20), indicating that results can be treated with high confidence.

## Discussion

### Conventional housing has strong, robust deleterious effects on health

Our hypothesis was that for laboratory rodents used in biomedical research, the behavioural restriction inherent in their conventional housing (CH) causes sufficient stress to impair functioning and compromise health. This hypothesis made two predictions: that CH would consistently increase the morbidity of induced stress-sensitive diseases, and also elevate all-cause mortality, over levels seen in ‘enriched’ housing (EH) that better supports species-typical behaviour and meets animals’ preferences. Using data from over 214 studies and over 6000 rodents, both predictions were met.

Conventional rodent housing thus significantly increased the severity of five stress-sensitive diseases. Effect sizes ranged from medium for cancer to very large for signs of depression. Only one slight discrepancy emerged: infarct volume was impacted less by housing than were other measures (with its SMD of 0.39). This was probably because after strokes were surgically induced, CH and EH animals were often both housed in isolated, barren cages for 24 h, and this period is when the majority of tissue damage occurs [91]. However even for this measure, the effect of subsequent housing was significant, suggesting that CH impaired lesion recovery. Overall, to summarize the impact of housing with one single SMD, the mean affect size was 0.74 (95% CI = 0.63–0.84). This means that CH exacerbated rodent morbidity with a medium to large effect. Such effects remained substantial after correcting for publication bias, and after eliminating weightings that were skewed by pseudoreplicative study reporting (SMD = 0.79, 95% CI = 0.67–0.90), an issue discussed further below. Effects also seemed consistent across rats and mice, socially housed and isolated animals, males and females, as well as across diseases. However, removing ‘red flags’ and controlling for infarct volume measures did reveal a second discrepancy: very large effects of CH exacerbating experimentally induced functional stroke outcomes (SMD = 1.63 [95% CI = 0.99-1.73]; compared to other diseases: SMD = 0.68 [95% CI = 0.53-0.83]). The robustness and reason for this needs future research.

Mortality rates were affected by housing too: for CH animals, the instantaneous hazard of dying was elevated by about 50%. Again such effects were not by-products of publication bias; they proved robust to eliminating weightings that were skewed by pseudoreplicative study reporting (hazard ratio = 1.61, 95% CI = 1.36–1.98), and they were also rather consistent, affected little by whether or not deaths arose from an experimentally induced disease, nor by animals’ species, sex or social environment. Similar to humans, in whom chronic stress elevates mortality for a range of disorders (e.g. [92, 93]), the cause of death in these studies was diverse. Mortality from experimentally induced disease varied widely (e.g. Huntington’s disease, amyotrophic lateral sclerosis and cardiomyopathy) and when disease was not induced, the cause of death was often unknown. However, effects were large enough that EH increased median survival by 9.3%. For context, this effect is greater than that of leading life-extending compounds resveratrol (which increases median survival by 4.1% [94]) and metformin (which increases mean lifespans by 5.8% [95]).

A meta-analysis is only as good as the studies its uses, and ours did show risks of experimental bias, not only in unit-of-analysis errors, but also in areas of blinding and cage randomization throughout the facility. Thus animal (rather than cage) was often used as the unit of replication, as is common in biomedical literature [87, 88]. This is an important pseudoreplicative error, since here the treatment (‘enrichment’) was applied to cages (not individuals) [87–90]. Furthermore, it was present in around one third of our studies using socially housed animals. However, rerunning our models excluding weights for inverse variance (which would be inflated for affected studies) had no substantive effect on the conclusions, likely because our total *N* was so large. The common lack of reported blinding was also concerning. However, the biases typically introduced by non-blind outcome assessment are smaller than our effect sizes: even the largest estimates, which suggest it inflates effect sizes by 0.19 [96], could not account for housing effects of the magnitude that we calculated. Furthermore, our assessment may sometimes reflect poor *reporting* rather than a true absence of blinding [97]: surveys indicate that some 20% of studies not reporting blinding did actually use it (suggesting that our true rate of blinding may be closer to 50%). The third prevalent risk of experimental bias was not reporting housing animals randomly throughout the room, which may lead to differential cage temperatures or light exposures [98, 99]. However, given the large number of studies and laboratories, this would only have contributed non-systematic error, not bias. Furthermore, collectively the quality of evidence was high, as assessed via GRADE guidelines. Overall, the results of this systematic review can therefore be treated with high confidence (with more data being unlikely to change these estimates of effect).

### Ethical and research implications

Conventional rodent cages are intended to meet ‘physical, physiologic and behavioural needs.’ [38], but whether they do so is generally not closely attended to. Describing housing is also not on the ‘essential’ list for the ARRIVE 2.0 reporting guidelines [86]. Thus while projects and procedures are regularly ethically reviewed, housing is not subject to the same scrutiny as long as it meets local minimum standards. Our findings reveal this to be a major ethical oversight. It has long been known that CH animals are behaviourally frustrated, at risk of ‘pessimism’, abnormal behaviour and impaired sleep, and low in resilience (as reviewed in the Introduction). CH can also have metabolic effects, rendering animals obese (especially rats) and hypothermic (especially mice) [77, 78, 80, 82], and CH generally compromises brain development [100, 101]. Our results now also demonstrate that as a result of this stress, CH rodents are consistently more vulnerable to mental and physical health problems: they become sicker when diseased, and die sooner than their EH counterparts. ILAR treats such signs of impaired adaptive capacity as evidence of ‘distress’ [53]. CH thus causes distress. In terms of regulation, using CH should logically therefore be treated as a stressful procedure (e.g. a ‘D’ in Canada, defined as ‘caus[ing] moderate to severe distress or discomfort’ [102]; an ‘E’ in the USA [‘stressful procedures that are not relieved with anaesthetics, analgesics and/or tranquilizers’ : [103], and ‘moderate’ in the EU [‘procedures that have caused moderate impairment of the well-being or general condition of the animals’: [35]).

Correspondingly, these results challenge two common assumptions in research projects that manipulate housing. The first is that the term ‘enriched’ is appropriate for housing that is not barren. As others have argued before us [104, 105], ‘enriched’—with its implications of ‘richness’—is probably not the best term for housing that is merely less poor. The second is that CH conditions represent a ‘control’, while improving them represents a ‘treatment’: a ubiquitous assumption made in the studies in this meta-analysis. If CH induces chronic stress, while adding key resources to CH helps animals meet their natural behavioural and thermoregulatory needs, it seems more logical to consider CH a deprivation treatment rather than a normal baseline (such that CH should be implemented only when a model of chronic stress is needed). Reassuringly, we also found no evidence that a move away from this poor housing would increase data variability (see also [106] and [107]). EH is thus unlikely to reduce statistical power.

Furthermore, this view of CH suggests another potential advantage to using rodent housing that is less poor: not just improved animal welfare, but perhaps also increased external validity. Currently, translatability rates are low: 86–91% of drugs that appear to work in animals fail in human clinical trials [108–112]. Some argue that a contributory factor is that data from stressed, sedentary, thermoregulatory-challenged animals are not relevant to people leading less-constrained lives (as outlined in the Introduction; e.g. [40–45, 113]). Our results confirm that housing does indeed have biologically significant impacts: a necessary condition for this hypothesis to be supported. However, our results are not *sufficient* evidence that CH contributes to the current translatability crisis. That is because our findings cannot identify whether housing has, not just quantitative, but also *qualitative*, interactive effects on research results (cf. e.g. [114]), such that data from CH or improved housing conditions *generate different conclusions.* (A figure illustrating this distinction is presented in Additional file 21). Investigating this hypothesis formally would take a new meta-analysis designed to do so. Nevertheless, consistent with this concern, it is already known that some results that look therapeutically promising in CH animals are weaker or abolished if subjects are better housed. For example, relevant to research on lifespan, some anti-oxidant effects of resveratrol in CH mice are diminished or even absent in better-housed conspecifics [115]; and likewise, in Alzheimer’s research, certain genetic mutations cause both amyloid plaques and cognitive deficits, but only in CH mice [116]. Conversely (but equally concerning), some null or adverse results in CH animals instead indicate promising therapies if subjects are better housed. For example, the harmful neurological side-effects of some novel anti-cancer agents on CH mice are diminished or even abolished in mice in less poor conditions [117]; in stroke research, epidermal growth factor does not improve recovery in CH rats, but does for rats in improved housing [118]; and flu vaccines which elicit only weak antigen-specific immunity in CH mice, have much greater benefits in better-housed conspecifics [119]. ‘Would conducting experiments under more than one set of conditions improve translation of knowledge to the clinic?’ ask Hylander & Repasky (2016) in *Trends in Cancer* [83]*.* The answer seems likely to be ‘yes’: a topic we visit below.

### What aspects of conventional housing are most impactful?

Housing effects did not seem influenced by the number and type of resources provided. This null result could indicate that CH is so deficient that ‘something, anything’ improves welfare [120]. However, we suspect it is more likely to be a Type II error resulting from the poor reporting of relevant information. Two thirds of studies did not describe their CH, leaving us assuming (perhaps incorrectly) that it merely met minimum standards. Furthermore, because animals were never observed within their home environments, we could not accurately evaluate how cages differed in their abilities to allow exercise, provide warmth and perceived safety, or support other species-typical behaviours. We thus could not assess *how* animals used resources (e.g. was a ‘structure’ used for climbing, or to nest within, or not at all? Was a ‘toy’ played with, despite subjects being adult, or was it gnawed, climbed on, or just ignored?). We could not assess *degrees* of use—important because rodents prefer some types of running wheel, and some types of nest boxes, more than others [19, 121]. We could not assess adverse reactions (for example, if grouped male mice were used, whether resources inadvertently triggered aggression [58, 59] was never reported). Finally, we could not evaluate how deprivation affected behavioural phenotypes: important because CH can promote either inactivity and weight gain [77, 78], or instead highly active stereotypic behaviour [25, 122]). Such knowledge gaps make it hard to assess which resources most reduce distress. We urge that as at least a *minimum* response to these problems, the reporting of animals’ housing conditions is moved to ARRIVE’s ‘essential’ list [86].

From a translatability perspective, such research and reporting gaps arguably also represent missed opportunities to strategically design housing in ways that model specific lived experiences, since by manipulating the types and extents of ‘enrichment’, researchers could differentially enhance specific aspects of animal environments. For example, such manipulations could parse out effects of exercise opportunities, being able to thermoregulate, being able to explore and become familiar with novelty and change, and/or having many other behavioural needs met. Furthermore, by systematically varying EH conditions, this approach could also reveal how robust effects are across a range of situations, thus potentially enhancing reproducibility as well as translatability [123, 124].

### Other incidental findings

The three viral infection studies found were not included in the meta-analysis, but two cautiously suggest an interesting exception to our pattern. For Dengue fever, infections were *less severe* in CH conditions [125, 126]. However, this disease has an unusual pathogenesis involving inflammatory hyperplasia; if confirmed, such effects are thus still consistent with high stress-suppressing immune responses [127, 128]. The third study, of *Vaccina*, found non-significant trends for wheel-running to protect against weight loss [129].

Some final findings warrant comment. Despite growing recognition of sex as a key biological variable, 80.4% of studies came from articles that used only one sex, and more than two thirds of these used males only (even in studies published since 2010; 72.6% [*n* = 90]). That research animal populations are commonly male-biased is a problem, since it under-represents female patients [130, 131], again potentially reducing translatability [132]. 31.8 % (*n* = 68) of studies also socially isolated their animals, including rats and female mice for whom this unambiguously reduces well-being [133–135]. Again this proportion was similar even for studies published since 2010 (29.3% [*n* = 44]). Finally, of the 33.2% of studies (*n* = 71), which described their CH conditions, 80.3% (*n* = 57) did not supply nesting or shelter (even in studies published since 2010; 80.3% [*n* = 61]). Thus if change is happening, it is slow.

## Conclusions

Our findings highlight the inadequacies of conventional cages for research rodents. They indicate that ‘enriched’ housing is not the luxury this term would imply, but instead something that helps meet animals’ basic needs by reducing distress. Furthermore, like relying on ‘WEIRD’ human subjects in psychology [136] and ‘STRANGE’ wild animals in ecology [137], our results, combined with previous work on both sex biases and the neurological and metabolic impacts of CH [25, 74, 77, 78, 80, 82, 130, 131], raise questions about data generalisability. Together, they indicate that typical research rodents should be termed ‘CRAMPED’ (cold, rotund, abnormal, male-biased, poorly surviving, enclosed and distressed). And so we end by asking, are results from CRAMPED rodents relevant to a wide cross-section of humans, including those who are fit and happy? If not, could rectifying their housing improve not only animal well-being, but also the translatability of biomedical research?

## Supplementary Information

**Additional file 1 **Diseases stated to be exacerbated by psychological stress, in the title and/or abstract of all papers cited in and citing [54] and [46] (citations from the main text). Citers catalogued *(April 1 2020, Google Scholar). (PDF 178 kb)*

**Additional file 2.** Outcomes and measures extracted for each disease.

**Additional file 3.** Pre-registered study protocol.

**Additional file 4.** Protocol amendments.

**Additional file 5.** PRISMA checklist.

**Additional file 6.** Database search strategy.

**Additional file 7.** Title/abstract screening and full text eligibility questions.

**Additional file 8.** Study level data collected. An article may contain multiple studies (where each study contains one set of animals under the same conditions described here).

**Additional file 9.** Results from random-effects meta-analyses rerun without study weights (cf. Figs. 3-9 which include study weights), comparing disease outcomes and mortality in conventionally housed and environmentally enriched animals.

**Additional file 10.** Results from a random-effects meta-regression rerun without study weights (cf. Table 1 which includes study weights), investigating potential moderators of housing effects on stress-sensitive disease (standardized mean differences). Bold p values are significant at p < 0.05.

**Additional file 11.** Results from a random-effects meta-regression rerun without study weights (cf. Table 2 which includes study weights), investigating potential moderators of housing effects on hazard ratio.

**Additional file 12.** R syntax used for data analysis.

**Additional file 13.** Studies excluded at full text article screening.

**Additional file 14.** Categorization of each resource.

**Additional file 15.** Study characteristics.

**Additional file 16.** A reference list of studies included in this systematic review.

**Additional file 17.** Risk of Bias assessment.

**Additional file 18.** Funnel plot for all stress-sensitive diseases. (A) Initial funnel plot including all stress-sensitive disease data. Blue dots indicate studies contributing to plot asymmetry (B) Funnel plot of studies included in final analysis with the publication bias removed. Bold line = null result, dotted line = standardized mean estimate calculated from all included studies. A plot with no publication bias should look symmetrical around the dotted line.

**Additional file 19.** A funnel plot of all studies included in the analysis reporting hazard ratios. Bold line = null result, dotted line = hazard ratio estimate calculated from all included studies.

**Additional file 20.** GRADE assessment of confidence of cumulative evidence. SMD = standardized mean difference.

**Additional file 21.** Hypothetical data illustrating how ‘enrichments’ could impact external validity. This figure demonstrates how interactive effects between a treatment (e.g. a drug) and EH could impact experimental conclusions. Note that in each graph the error bars do not change, as EH does not change data variability. Also note that EH is not one unitary thing, but something that can vary in kind and degree (for example to deliberately introduce heterogeneity). (A) No interactive effect. No matter the cage condition, the experimental conclusion is the same: the drug reduces disease but does not cure it. EH does not affect external validity. (B) The drug effect is absent with EH. This drug could be useful for specific populations (e.g. chronically stressed and/or overweight subjects) but not others (e.g. physically fit content subjects). Testing the drug only under CH conditions will generate false positives, unless the target population is specifically one which is stressed and/or overweight etc. (C) The drug effect is only detectable in EH. This suggests the drug could be useful for some populations (e.g. ones which are physically fit and content) but not others (e.g. chronically stressed and/or overweight) Testing the drug only under CH conditions will generate false negatives, unless the target population is specifically one which is stressed and/or overweight.

## Data Availability

All data supporting the conclusions of this article are included within the article and supplemental materials. Additional raw data is available upon request.
