# Supplementary material for: Conventional laboratory housing increases morbidity and mortality in research rodents: results of a meta-analysis
Source: BMC Biol. 2022 Jan 13;20:15. doi: 10.1186/s12915-021-01184-0 (PMC8756709; doi:10.1186/s12915-021-01184-0)
Supplement: Supplementary file 1 — Additional file 1 Diseases stated to be exacerbated by psychological stress, in the title and/or abstract of all papers cited in and citing [54] and [46] (citations from the main text). Citers catalogued (April 1 2020, Google Scholar). (PDF 178 kb) [file 12915_2021_1184_MOESM1_ESM.pdf]

**disease**	**# papers**
cardiovascular disease	23
depression	15
cancer	10
viral infection	8
asthma	4
anxiety	3
stroke	2
