# Supplementary material for: Conventional laboratory housing increases morbidity and mortality in research rodents: results of a meta-analysis
Source: BMC Biol. 2022 Jan 13;20:15. doi: 10.1186/s12915-021-01184-0 (PMC8756709; doi:10.1186/s12915-021-01184-0)
Supplement: Supplementary file 2 — Additional file 2. Outcomes and measures extracted for each disease. [file 12915_2021_1184_MOESM2_ESM.pdf]

Disease	Key references on stress exacerbation of disease	Disease Induction	Outcomes	Measures	Experimental readouts
anxiety disorders	humans: [1] [2]<br><br>rodents: [3] [4] [5] [6] [7]	uncontrollable/unpredictable stress, acute or chronic restraint stress, maternal separation, early weaning/social isolation stress, social defeat stress, olfactory bulbectomy, chronic corticosterone administration or genetic susceptibility/transgenic	behavioural measures of anxiety	Elevated Plus Maze	% time spent in open arm
				Open Field Test	% time spent in center, total freezing time, rearing, defecation
				Light/Dark Box	% time in light side
				Social Interaction Test	% time spent interacting
asthma	humans: [8] [9] [10] [11]<br><br>rodents: [12] [13] [14]	induced allergic airway inflammation: OVA/alum, House Dust Mite or papain	total cell number	total cell number in lung tissue or airspaces	cell count
			eosinophils	total cell number in lung tissue or airspaces	cell count
			cytokine levels	serum levels of IL-4, IL-5 and IFN-γ	protein levels (pg/mL, ug/mL) or mRNA expression (ΔΔCT values)
cancer	humans: [15] [16] [17-19]<br><br>rodents: [20] [21] [22]	injected tumor cells “spontaneous” tumor development: transgenic animals, drug induced	tumor burden	tumor volume	mm^3
				tumor weight	in milligrams
				number of animals with tumors	cross tabulated tables or percentages
				metastasis	number of metastasis or cell count
				number of tumors	tumor count
cardiovascular disease	humans: [23] [24] [25]<br><br>rodents: [26] [27] [28]	transgenic animals (apoE knockout) fed high fat or high cholesterol diets	intimal thickening	histological assessment	mm^2 or % of lumen filled
			myocardial infarction incidence	sudden death	cross tabulated tables or percentages
major depression	humans: [29] [30] [31]<br><br>rodents: [32] [33] [34] [35]	uncontrollable/unpredictable stress, acute or chronic restraint stress, maternal separation, early weaning/social isolation	anhedonia	sucrose preference	% sucrose/ total drinking volume
			helplessness	Forced-Swim Tests	total time immobile

		stress, social defeat stress, olfactory bulbectomy, chronic corticosterone administration or genetic susceptibility/transgenic		Tail Suspension Tests	total time immobile
			hippocampal volume	volume measurements by MRI or histological sections	% of brain volume or total volume in mm^3
stroke	humans: [36] [37] [38]<br><br>rodents: [39] [40] [41]	surgical: middle cerebral artery occlusion (MCAO) model, photothrombotic stroke (Rose Bengal), endothelin-1	infarct volume	histological assessment (e.g. TTC, H&E)	mm^3 or % of hemisphere
			motor and sensorimotor tests	composite scores (Benderson scale, Modified Neurological Severity Score, other scoring systems specified by the authors)	ordinal score data
				Accelerated Rotarod	mean fall score (RPM) or score relative to baseline or latency to fall
				Ledge Tapered Beam Test	foot faults or distance traveled
				Pasta Test	number of paw adjustments
				Limb Placement Test	ordinal score data
			cognitive impairment	Morris Water Maze	% time spent in target quadrant or path length
				Morris Water Maze (reversal learning)	% time spent in target quadrant or path length
viral infections	humans: (44) [42] [43, 44] [45] [46]	infection with known viral quantities	viral load	viral load in relevant organ or blood (e.g. RT-PCR)	viral copy number

	rodents: [47] [48] [49]		cytokine levels	serum or relevant organ levels of IL-1b, IL-6 and TNFα	protein levels (pg/mL, ug/mL) or mRNA expression (ΔΔCT values)
			weight loss	rodent weight	% change from baseline or total weight lost

References

1. Heim C, Nemeroff CB. The role of childhood trauma in the neurobiology of mood and anxiety disorders: preclinical and clinical studies. Biological Psychiatry. 2001;49(12):1023-39.
2. Platt R, Williams SR, Ginsburg GS. Stressful life events and child anxiety: Examining parent and child mediators. Child Psychiatry & Human Development. 2016;47(1):23-34.
3. Chiba S, Numakawa T, Ninomiya M, Richards MC, Wakabayashi C, Kunugi H. Chronic restraint stress causes anxiety-and depression-like behaviors, downregulates glucocorticoid receptor expression, and attenuates glutamate release induced by brain-derived neurotrophic factor in the prefrontal cortex. Progress in Neuro-Psychopharmacology and Biological Psychiatry. 2012;39(1):112-9.
4. Hammack SE, Cheung J, Rhodes KM, Schutz KC, Falls WA, Braas KM, et al. Chronic stress increases pituitary adenylate cyclase-activating peptide (PACAP) and brain-derived neurotrophic factor (BDNF) mRNA expression in the bed nucleus of the stria terminalis (BNST): roles for PACAP in anxiety-like behavior. Psychoneuroendocrinology. 2009;34(6):833-43.
5. Daniels W, Pietersen C, Carstens M, Stein D. Maternal separation in rats leads to anxiety-like behavior and a blunted ACTH response and altered neurotransmitter levels in response to a subsequent stressor. Metabolic Brain Disease. 2004;19(1):3-14.
6. Golden SA, Covington III HE, Berton O, Russo SJ. A standardized protocol for repeated social defeat stress in mice. Nature protocols. 2011;6(8):1183.
7. Kinsey SG, Bailey MT, Sheridan JF, Padgett DA, Avitsur R. Repeated social defeat causes increased anxiety-like behavior and alters splenocyte function in C57BL/6 and CD-1 mice. Brain, behavior, and immunity. 2007;21(4):458-66.
8. Sandberg S, Paton JY, Ahola S, McCann DC, McGuinness D, Hillary CR, et al. The role of acute and chronic stress in asthma attacks in children. The Lancet. 2000;356(9234):982-7.
9. Wright RJ, Rodriguez M, Cohen S. Review of psychosocial stress and asthma: an integrated biopsychosocial approach. Thorax. 1998;53(12):1066-74.
10. Marin TJ, Chen E, Munch JA, Miller GE. Double-exposure to acute stress and chronic family stress is associated with immune changes in children with asthma. Psychosomatic medicine. 2009;71(4):378-84.
11. Höglund CO, Axen J, Kemi C, Jernelöv S, Grunewald J, Müller-Suur C, et al. Changes in immune regulation in response to examination stress in atopic and healthy individuals. Clinical & Experimental Allergy. 2006;36(8):982-92.
12. Chida Y, Sudo N, Sonoda J, Hiramoto T, Kubo C. Early-life psychological stress exacerbates adult mouse asthma via the hypothalamus–pituitary–adrenal axis. American journal of respiratory and critical care medicine. 2007;175(4):316-22.
13. Bei L, Duan X-h, Wu J-F, Liu B-j, Luo Q-L, Jin H-l, et al. Impact of psychosocial stress on airway inflammation and its mechanism in a murine model of allergic asthma. Chinese medical journal. 2013;126(2):325-34.
14. Qian L, Lu L, Huang L, Wen Q, Xie J, Jin W, et al. The effect of neonatal maternal separation on short-chain fatty acids and airway inflammation in adult asthma mice. Allergologia et immunopathologia. 2019;47(1):2-11.
15. Soung NK, Kim BY. Psychological stress and cancer. Journal of Analytical Science and Technology. 2015;6(1):30.
16. Andersen BL, Kiecolt-Glaser JK, Glaser R. A biobehavioral model of cancer stress and disease course. American psychologist. 1994;49(5):389.
17. Bovbjerg DH. Psychoneuroimmunology. Implications for oncology? Cancer. 1991;67(S3):828-32.
18. Levy S, Herberman R, Lippman M, d'Angelo T. Correlation of stress factors with sustained depression of natural killer cell activity and predicted prognosis in patients with breast cancer. Journal of Clinical Oncology. 1987;5(3):348-53.

19. Lillberg K, Verkasalo PK, Kaprio J, Teppo L, Helenius H, Koskenvuo M. Stressful life events and risk of breast cancer in 10,808 women: a cohort study. American journal of epidemiology. 2003;157(5):415-23.
20. Saul AN, Oberyszyn TM, Daugherty C, Kusewitt D, Jones S, Jewell S, et al. Chronic stress and susceptibility to skin cancer. Journal of the National Cancer Institute. 2005;97(23):1760-7.
21. Steplewski Z, Vogel WH, Ehya H, Poropatich C, Smith JM. Effects of restraint stress on inoculated tumor growth and immune response in rats. Cancer research. 1985;45(10):5128-33.
22. Nakamura T, Walker A, Sominsky L, Allen T, Rosengren S, Hodgson DM. Maternal separation in early life impairs tumor immunity in adulthood in the F344 rat. Stress (Amsterdam, Netherlands). 2011;14(3):335-43.
23. Steptoe A, Kivimaki M. Stress and cardiovascular disease. Nat Rev Cardiol. 2012;9(6):360-70.
24. Rozanski A, Blumenthal JA, Kaplan J. Impact of psychological factors on the pathogenesis of cardiovascular disease and implications for therapy. Circulation. 1999;99(16):2192-217.
25. Dimsdale JE. Psychological stress and cardiovascular disease. Journal of the American College of Cardiology. 2008;51(13):1237-46.
26. Kumari M, Grahame-Clarke C, Shanks N, Marmot M, Lightman S, Vallance P. Chronic stress accelerates atherosclerosis in the apolipoprotein E deficient mouse. Stress (Amsterdam, Netherlands). 2003;6(4):297-9.
27. Gu H, Tang C, Peng K, Sun H, Yang Y. Effects of chronic mild stress on the development of atherosclerosis and expression of toll-like receptor 4 signaling pathway in adolescent apolipoprotein E knockout mice. Journal of biomedicine and biotechnology. 2009;2009.
28. Zhang T, Zhai Y, Chen Y, Zhou Z, Yang J, Liu H. Effects of emotional and physiological stress on plaque instability in apolipoprotein E knockout mice. Journal of Physiology and Biochemistry. 2011;67(3):401.
29. Psychiatric history and stress: Predictors of severity of unipolar depression [press release]. US: American Psychological Association1992.
30. McGonagle KA, Kessler RC. Chronic stress, acute stress, and depressive symptoms. American journal of community psychology. 1990;18(5):681-706.
31. Kim EJ, Pellman B, Kim JJ. Stress effects on the hippocampus: a critical review. Learning & memory (Cold Spring Harbor, NY). 2015;22(9):411-6.
32. Vetulani J. Early maternal separation: a rodent model of depression and a prevailing human condition. Pharmacological reports. 2013;65(6):1451-61.
33. Forbes NF, Stewart CA, Matthews K, Reid IC. Chronic mild stress and sucrose consumption: validity as a model of depression. Physiology & Behavior. 1996;60(6):1481-4.
34. Willner P. Validity, reliability and utility of the chronic mild stress model of depression: a 10-year review and evaluation. Psychopharmacology. 1997;134(4):319-29.
35. Tsankova NM, Berton O, Renthal W, Kumar A, Neve RL, Nestler EJ. Sustained hippocampal chromatin regulation in a mouse model of depression and antidepressant action. Nature neuroscience. 2006;9(4):519-25.
36. Palmer S, Glass TA. Family function and stroke recovery: a review. Rehabilitation Psychology. 2003;48(4):255-65.
37. Payabvash S, Taleb S, Benson JC, McKinney AM. Acute Ischemic Stroke Infarct Topology: Association with Lesion Volume and Severity of Symptoms at Admission and Discharge. American Journal of Neuroradiology. 2017;38(1):58-63.
38. Aron AW, Staff I, Fortunato G, McCullough LD. Prestroke living situation and depression contribute to initial stroke severity and stroke recovery. Journal of Stroke and Cerebrovascular Diseases. 2015;24(2):492-9.
39. Faraji J, Ejaredar M, Metz GA, Sutherland RJ. Chronic stress prior to hippocampal stroke enhances post-stroke spatial deficits in the ziggurat task. Neurobiology of Learning and Memory. 2011;95(3):335-45.

40. Merrett DL, Kirkland SW, Metz GA. Synergistic effects of age and stress in a rodent model of stroke. Behavioural brain research. 2010;214(1):55-9.
41. Kirkland SW, Coma AK, Colwell KL, Metz GA. Delayed recovery and exaggerated infarct size by post-lesion stress in a rat model of focal cerebral stroke. Brain research. 2008;1201:151-60.
42. Ironson G, O'Cleirigh C, Fletcher MA, Laurenceau JP, Balbin E, Klimas N, et al. Psychosocial factors predict CD4 and viral load change in men and women with human immunodeficiency virus in the era of highly active antiretroviral treatment. Psychosom Med. 2005;67(6):1013-21.
43. Zimmerman G, Shaltiel G, Barbash S, Cohen J, Gasho CJ, Shenhar-Tsarfaty S, et al. Post-traumatic anxiety associates with failure of the innate immune receptor TLR9 to evade the pro-inflammatory NFκB pathway. Translational Psychiatry. 2012;2(2):e78-e.
44. Cohen S, Doyle WJ, Skoner DP. Psychological Stress, cytokine production, and severity of upper respiratory illness. Psychosom Med. 1999;61(2):175-80.
45. Brydon L, Walker C, Wawrzyniak A, Whitehead D, Okamura H, Yajima J, et al. Synergistic effects of psychological and immune stressors on inflammatory cytokine and sickness responses in humans. Brain, behavior, and immunity. 2009;23(2):217-24.
46. Wolf JM, Rohleder N, Bierhaus A, Nawroth PP, Kirschbaum C. Determinants of the NF-κB response to acute psychosocial stress in humans. Brain, behavior, and immunity. 2009;23(6):742-9.
47. Zhang Y, Zhang Y, Miao J, Hanley G, Stuart C, Sun X, et al. Chronic restraint stress promotes immune suppression through toll-like receptor 4-mediated phosphoinositide 3-kinase signaling. Journal of Neuroimmunology. 2008;204(1-2):13-9.
48. Hunzeker J, Padgett DA, Sheridan PA, Dhabhar FS, Sheridan JF. Modulation of natural killer cell activity by restraint stress during an influenza A/PR8 infection in mice. Brain, behavior, and immunity. 2004;18(6):526-35.
49. Luo Z, Liu L-F, Jiang Y-N, Tang L-P, Li W, Ouyang S-H, et al. Novel insights into stress-induced susceptibility to influenza: corticosterone impacts interferon-β responses by Mfn2-mediated ubiquitin degradation of MAVS. Signal Transduction and Targeted Therapy. 2020;5(1):202.
