# Supplementary material for: Conventional laboratory housing increases morbidity and mortality in research rodents: results of a meta-analysis
Source: BMC Biol. 2022 Jan 13;20:15. doi: 10.1186/s12915-021-01184-0 (PMC8756709; doi:10.1186/s12915-021-01184-0)
Supplement: Supplementary file 3 — Additional file 3. Pre-registered study protocol. [file 12915_2021_1184_MOESM3_ESM.pdf]

Title: The impact of environmental enrichment on laboratory rodent health: a protocol for a systematic review.

Authors: Jessica Cait[1], Alissa Cait[2], Charlotte B Winder[3] and Georgia J Mason[4*]

[1] Department of Animal Biosciences, Ontario Agricultural College, University of Guelph, Guelph, ON, Canada, N1G 2W1. caitj@uoguelph.ca
[2] alissacait@gmail.com
[3] Department of Population Medicine, Ontario Veterinary College, University of Guelph, Guelph, ON, Canada, N1G 2W1. winderc@uoguelph.ca
[4] Department of Integrative Biology, College of Biological Science, University of Guelph, Guelph, ON, Canada, N1G 2W1. gmason@uoguelph.ca
* corresponding author

Author contributions: GJM is the guarantor. JC will be the review leader. JC will perform all literature screening, data extraction and risk of bias assessments. AC and an undergraduate student will act as the secondary reviewers and will each perform half of the literature screening, data extraction and risk of bias assessments. JC will write and prepare all manuscript drafts (for the protocol and manuscript), and content expertise will be provided by GJM when needed. CBW will be consulted as methodological expert and aid in statistical analyses. All authors will review and approve the final version of the manuscript.

Registration: This protocol is archived in the University of Guelph's repository (The Atrium: https://atrium.lib.uoguelph.ca/xmlui/handle/10214/10046) and published online with Systematic Reviews for Animals and Food (SYREAF) (http://www.syreaf.org/contact/). This protocol was developing using the Preferred Reporting Items for Systematic Reviews and Meta-Analyses - Protocol (PRISMA-P) guidelines (Moher et al., 2015).

Amendments: Protocol deviations made following the protocol registration will be documented in the final systematic review accompanied by the date of the change, a description of the change and the rationale.

Support: This work is supported by the Natural Sciences and Engineering Council of Canada (NSERC) discovery grant awarded to GJM. Stipend funding for JC was provided by Alexander Graham Bell Canada Graduate Scholarships – Doctoral (NSERC CGD-D).

INTRODUCTION.

Rationale: The relationship between psychological distress and health is complex, and the mechanisms underlying the interactions of immune, behavioural and neuroendocrine systems are not fully elucidated. In general terms, however, neuroendocrine hormones triggered by psychological stress can lead to dysregulation of immune function. This can paradoxically result in both the inappropriate suppression and excessive activation of the immune system, both of which then lead to the progression of disease (Segerstrom & Miller, 2004). In humans, subjective assessment of a stressful life as well as negative life events can therefore increase the risk of developing as well as exacerbating the symptoms of diseases such as cancer (Chida, Hamer, Wardle, & Steptoe, 2008), major depression (Horesh, Klomek, & Apter, 2008), stroke (Li, Zhang, Hou, & Tang, 2015) and cardiovascular disease (Steptoe & Kivimaki, 2012). Stress can even increase all-cause mortality (Rutters et al., 2014) and thus reduce lifespans (Stringhini et al., 2012). Conversely, positive social relationships and self-reported happiness can reduce

the risk of disease, attenuate disease severity as well as increase life expectancy (Lawrence, Rogers, & Wadsworth, 2015). My aim here is to ascertain whether similar effects occur in laboratory rodents.

Laboratory guidelines state that "all *animals* should be housed under conditions that provide sufficient space as well as supplementary structures and resources required to meet physical, physiologic, and behavioral needs." (National Research Council, 2011). However, the minimum housing standards in current practice do not sufficiently fulfill this definition. The standard laboratory rodent cage is small: smaller than the size of a shoebox (330cm^2) for mice, and < 600cm^2 for rats (Canadian Council on Animal Care, 2019) (National Research Council, 2011), with a granular flooring substrate (e.g. corncob or wood chips) and nesting material (which is not always included in standard housing but should be, as nests are essential for thermoregulation (Gaskill, Gordon, et al., 2013) (Gaskill, Pritchett-Corning, et al., 2013) (Hankenson, Marx, Gordon, & David, 2018)). In these small barren cages, rodents are unable to perform many motivated natural behaviours such as climbing, burrowing and standing upright (Makowska & Weary, 2016). To tackle the inadequacies of standard housing, researchers can increase cage complexity by adding environmental enrichments (Canadian Council on Animal Care, 2019) (National Research Council, 2011). Conversely, large enriched cages allow rodents to perform these behaviours and as a result these cages are motivating: rats show levels of anticipatory behaviour equivalent to that of rats anticipating sexual contact when awaiting transfer to an enriched cage (van der Harst, Fermont, Bilstra, & Spruijt, 2003) and mice show equal motivation to access enrichment as they do to access food and will even traverse shallow water multiple times a day to access enrichment items (Sherwin, 1996). Abnormal behaviours, widely used welfare indicators which are common in captive laboratory rodents are also reduced or even completely absent in animals housed in enriched cages (G. J. Mason, Latham, N, 2004) (Bailoo et al., 2018; Bechard, Meagher, & Mason, 2011; Fureix et al., 2016). Taken together, the inability to perform natural behaviours as well as the emergence of abnormal behaviours highlight some of the inadequacies of captive environments. More than this, they may also indicate resultant changes to rodent wellbeing. Standard housed rodents show signs of psychological distress (Olsson & Dahlborn, 2002) (Novaes et al., 2017), signs of depression (Fureix et al., 2016) (Chourbaji et al., 2005) (Seong, Park, & Kim, 2018) and signs of chronic stress (Chourbaji et al., 2005) that are all reduced in enriched cages.

Throughout the biomedical literature, enrichment has been used as an experimental manipulation to study how complex environments can stimulate the brain and downstream physiological processes. The purpose of this review is to use those studies to understand how the environmental conditions for caged laboratory animals impact their ability to cope with health challenges. Disease models serve as predictable insults with clearly anticipated and well defined outcomes. By challenging the physiological system, disease models can magnify sub-clinical health problems and abnormalities that may be difficult to define or measure under naïve conditions. These properties allow us to use disease models to assess whether standard rodent housing has similar deleterious effects on health as seen in stressed humans experiencing exacerbated disease. However, although some specific diseases are more frequently associated with the experience of stressful events (e.g. cardiovascular diseases or autoimmune disorders) (Miller, Chen, & Parker, 2011) psychological stress can reduce lifespans non-specifically, that is without a specified disease cause (Morris, Cook, & Shaper, 1994). Rodent models can model disease progression and recovery once the animal is already ill, but they cannot model the spontaneous loss of health we see in humans. For this reason, we add a second type of study, ones assessing all-cause mortality. This allows us to ask two research questions: 1) do rodents in standard cages have curtailed lives? and 2) are they more vulnerable to stress-sensitive diseases in modelling research?

We thus hypothesize that standard housing conditions have deleterious effects on animal health. This is based on assumptions that enrichments do indeed improve welfare and reduce stress: an assumption that does not always hold. For instance, enrichments changed too frequently can elicit neophobic

responses reducing enrichment benefits or potentially causing harm (Walker & Mason, 2011). A prerequisite for this study is thus selecting enrichments that *a priori* should be appropriate and stress-reducing. Then, if this hypothesis is true, the prediction follows that the provision of appropriately enriched environments will improve rodent health such that they experience attenuated disease morbidity and increased lifespans. To perform this review, we limit our analysis to all-cause mortality and biomedical diseases recapitulating aspects of human diseases shown to be worsened by psychological stress, predicting that mice and rats in enriched environments will fare better in a wide array of disease models. We generated a list of seven diseases: cardiovascular disease, major depression, cancer, viral infection, asthma, anxiety disorders and stroke. From our hypothesis the prediction arises that the same disease outcomes observed to be exacerbated by stress in humans will also be exacerbated by standard housing conditions: increased atherosclerotic lesions in models of cardiovascular disease; increased anhedonia in models of depression; increased tumor burden in cancer models; increased viral load during viral infections; increased inflammation in models of asthma; increased anxiety behaviours in models of anxiety disorders; and reduced motor control and impaired learning and memory post-stroke. We suggest that disease severity and all-cause mortality are excellent indicators of overall animal health and wellbeing; thus, if enrichment attenuates disease outcomes and reduces mortality this suggests that current housing practices do not meet the standard of meeting the "physical, psychologic and behavioural needs" of the animal.

Defining key terms: Three important terms are defined here to clarify the screening and data extraction criteria below.

Disease: "An impairment of the normal state of the living animal or plant body or one of its parts that interrupts or modifies the performance of the vital functions, is typically manifested by distinguishing signs and symptoms, and is a response to environmental factors (as malnutrition, industrial hazards, or climate), to specific infective agents (as worms, bacteria, or viruses), to inherent defects of the organism (as genetic anomalies), or to combinations of these factors." (Merriam-Webster Dictionary)

Model: A model needs to be defined separately from a test. "Defining a protocol as a "test" or a "model" is not a mere matter of terms, but it capitalizes on the difference between measuring a response *versus* evoking a pathology." (Lampis, Maziade, & Battaglia, 2011). Based on this description, we define a disease model as one evoking a pathology.

Environmental enrichment: Environmental enrichment will be defined as any structural item added to the cage determined by the publishing authors to be enrichment beyond nesting material alone. This will not include other rodents, olfactory, gustatory or auditory enrichment.

Objectives: The objective of this protocol is to define the methods for a systematic review to assess the impact of environmental enrichment on rodent health in laboratory research. The specific review question to be addressed in this protocol and following systematic review is as follows:

For experimental mice and rats, does environmental enrichment impact health compared to standard housing?

i. *Population:* laboratory mice and rats.
ii. *Intervention:* environmentally enriched housing
iii. *Comparator:* standard laboratory housing
iv. *Outcomes:* rodent health (mortality data or disease specific morbidity data. See below.)

Further defining the scope of the research question: The term *rodent health* (the outcome measure in the PICO question) needs further definition. We determined *rodent health* based on disease specific morbidity as well as all-cause mortality data from laboratory rodent research. We determined a list of relevant diseases by focusing on ones found to be exacerbated by stress in humans. A plethora of research is available on this topic (e.g. a Medline search of “psychological stress” and “disease” generated 1,927 hits on May 18, 2020). We therefore used two previous reviews as the foci for our searches. One was a review previously completed by GJM (G. Mason, Walker, Duggan, Roulston, & Van Slack, 2012); and a more recent review (Cohen, Murphy, & Prather, 2019). We hand searched the title and abstracts from all references from these two reviews, as well as papers citing these reviews (**Table 1**). From these citations we listed all diseases mentioned as being exacerbated by psychological stress in humans and came up with a list of seven diseases: cardiovascular disease, major depression, cancer, viral infection, asthma, anxiety disorders and stroke.

Table 1. A list of diseases mentioned to be exacerbated by psychological stress in the title and/or abstract of (G. Mason et al., 2012) or (Cohen et al., 2019) and total disease mentions.

disease	mentions
cardiovascular disease	23
depression	15
cancer	10
viral infection	8
asthma	4
anxiety	3
stroke	2

Outcomes: Each outcome measure extracted will be specific to the disease model specified. Due to the breadth of the research question and the numerous potential outcomes used in each study, we generated a short list of outcomes that are most relevant to addressing our research question. To do this, we chose relevant outcomes by defining which human outcomes have been negatively impacted by stress, and cross-referencing those outcomes with ones commonly reported in biomedical rodent literature (not specific to enrichment literature). If the human literature mentioned specific disease outcomes exacerbated by stress and if rodent models had comparable outcome measures, we included these in our study. Unfortunately, not all of our rodent models use the same outcome measures as human disease. In that case, we took the rodent outcomes most similar to (or representative of) outcomes measured in humans. Rationales for these outcomes are discussed below. We chose to limit our focus to a maximum of three outcomes per disease to maintain the focus and feasibility of the study.

METHODS.

Eligibility criteria:

Intervention: Enrichment must be a factor of the home environment and interventions with access to enriched environments outside the home cage will be excluded. A description or clear image of the enriched environment must be available in the article or supplementary material. A statement that a cage is enriched will not suffice as a description. Any study without a description or clear image of the enriched intervention will be excluded.

Comparator: The study must contain a comparator group with the same type of social housing as the intervention (e.g. singly-housed, pair or group-housed animals). Since it is common for authors to inadequately report details of the standard housing conditions, we will include all studies regardless of the detail provided in the description of standard conditions (please see data extraction below for more details).

Report Characteristics: The study must be published in English and the article must report primary data.

Study Designs: Only *in vivo* studies will be included. We assume that all relevant studies will be controlled trials therefore no observational studies will be included.

Information sources:

Table 2. Databases and information sources to be searched.

**Database/Information Source**	**Interface/URL**
MEDLINE	Ovid
CAB abstracts	CAB interface
Science Citation Index	Web of Science
Proquest Disertations and Theses A&I (grey literature)	Proquest
Elsevier	SCOPUS

Search strategy: No limits on study design, date or language limits will be imposed on the search beyond that of the databases themselves, although only studies in English will be included in the review due to resource limits. The specific search strategy was created in MEDLINE (OVID interface, 1948 onwards) by JC with expertise and input from a health sciences librarian with expertise in systematic review searching. After generation of the MEDLINE strategy, the search will be adapted to the syntax and subject headings of the other databases listed above.

Table 3. An example search from May 21, 2020. The literature search strategy was developed using medical subject headings (MeSH) and text words related to our population (mouse and rat), intervention (environmental enrichment) and outcome (disease) topics. This search strategy was designed in MEDLINE (Ovid) and adapted for other databases. ti= title, kw= author keywords, ab= abstract exp= explode.

	**Search**	**Results**
#1	exp Murinae/	
#2	(mice OR mouse OR Mus OR rodent* OR murine OR rat OR rats).ti,kw,ab.	
#3	#1 OR #2	3355059
#4	Housing, Animal/	
#5	((cage OR caging OR caged OR cages OR environment*) adj3 (enrich* OR naturalistic)).ti,kw,ab.	
#6	(“voluntary wheel running" or "running wheel" or "wheel running" or "running disk" or “physical activity”).ti,kw,ab.	
#7	#4 OR #5 OR #6	123290
#8	Cardiovascular Diseases/	

#9	("cardiovascular disease*" or "coronary artery disease" or "myocardial infarct*" or "coronary heart disease" or "atherosclero*" or "arteriosclero* myocardial ischemia" or "ischemic heart disease" or "coronary heart disease" or "APOE" or "Apolipoprotein E" or "intimal thickening" or "lumen stenosis" or "lumen occlusion*" or "atherogenic diet*" or "coronary lesion*").ti,kw,ab.	
#10	#8 OR #9	655148
#11	Depressive Disorder/ or Depressive Disorder, Major/	
#12	("model of depression" or "major depression" or "major depressive disorder" or "depressive disorder" or "forced-swim* test" or "forced swim* test" or "anhedonia" or "sucrose preference" or "social defeat stress" or "tail suspension test" or "chronic mild stress" or "learned helplessness" or "olfactory bulbectomy" or "maternal separation" or "chronic restraint stress").ti,ab,kw.	
#13	#11 OR #12	131738
#14	exp Neoplasms/	
#15	(carcino* or cancer or malignant or tumor or tumour).ti,kw,ab.	
#16	#14 OR #15	4149681
#17	exp Viruses/ or exp Virus/ or Virus Diseases/	
#18	("viral infection" or virus or "immunodeficiency virus" or HIV or "infectious disease" or "respiratory disease" or "upper respiratory disease" or influenza).ti,kw,ab.	
#19	#17 OR #18	1238126
#20	Asthma/	
#21	(OVA or asthma or asthmatic or "house dust mite" or "papain" or "atopic" or "allergic lung inflammation").ti,ab,kw.	
#22	#20 OR #21	221300
#23	Anxiety/ or Anxiety Disorders/	
#24	("models of anxiety" or "anxiety disorder*" or "anxiety" or "anxious" or "general anxiety" or "material separation" or anxiogenic).ti,kw,ab.	
#25	#23 OR #24	218493
#26	Stroke/	
#27	("stroke" or "cerebrovascular disease" or "cerebrovascular disorders" or "cerebral infarct" or "ischemic stroke" or "intracranial hemorrhage" or "intracranial artery disease" or "middle cerebral artery occlusion" or "MCAO").ti,kw,ab.	
#28	#26 OR #27	274786
#29	exp Aging/	
#30	(longevity or mortality or survivorship or "survival rate" or survival).ti,kw,ab.	
#31	#29 OR #30	1774382
#32	#10 OR #13 OR #16 OR #19 OR #22 OR #25 OR #28 OR #31	7452116
#33	#3 AND #7 AND #32	2692

STUDY RECORDS.

Data management: The results from the searches will be downloaded into a bibliographic software EndNote X7™ (Clairvate Analytics, Philadelphia, USA) and de-duplicated using EndNote's™ algorithms. De-duplicated references will be loaded into the online reference software DistillerSR® (Evidence

Partners, Ottawa, Canada). Prior to screening, a pilot test on the first 100 records for title/abstract, and first 25 records for full text, will be conducted to ensure consistent data collection between reviewers.

Selection process: Two independent reviewers will perform the selection process and risk of bias assessment. This review will have two selection stages. The first, scanning the title and abstract for eligibility and the second, reading whole text articles for eligibility.

Stage 1, scanning title and abstract:

i. Is the title and/or abstract available in English?
ii. Does the title and/or abstract describe a primary *in vivo* research trial?
iii. Does the title and/or abstract use laboratory mice or rats for their study?
iv. Does the title and/or abstract use environmental enrichment as an intervention?
v. Does the title and/or abstract report the use of one of the disease models of interest and/or study survival/mortality?

All questions will have a response YES, NO and UNCLEAR. Any study receiving a NO for any question will be excluded.

Stage 2, whole text articles:

i. Is the study available in English?
ii. Is it a primary *in vivo* research trial?
iii. Do the researchers use laboratory mice or rats in the study?
iv. Does the study use appropriate environmental enrichment (as described in the exclusion criteria) as an intervention?
v. Does the study use appropriate (as described in the exclusion criteria) standard housing as a comparator?
vi. Does the study include one of the disease models of interest (i.e. cardiovascular disease, major depression, cancer, viral infection, asthma, anxiety disorders, stroke and/or a mortality study)?
vii. Does the study measure any of the outcomes listed in **Table 4** or report survival/mortality data?

All questions will have a response YES or NO. Answers to question vi. will be YES or NO, but an answer of YES will generate a list of possible biomedical disease models for data extraction. A reference will only be excluded if both reviewers answer NO to any screening question. Any conflicts will be resolved by consensus. If consensus cannot be reached, a third person on the review team will be consulted. Any study receiving a NO for any question will be excluded.

Data extraction process: Two reviewers will extract data from eligible studies independently using a form created in DistillerSR®. This form will be pilot tested using 10 references by all reviewers in order to ensure consistency in data extraction. Authors will not be contacted to request missing data or to clarify published results unless there is an obvious or apparent typo (e.g. flipped axis labels are contradictory to the author's conclusion and text results). In this case, authors will be contacted once and no follow up contact will be made.

Data items:

The following information will be extracted:

i. *Study ID:* authors, title, year and journal
ii. *Study design characteristics:* sample size for control and treatment groups
iii. *Animal model characteristics:* species, strain, genotype (if applicable) sex, age
iv. *Intervention and comparator characteristics:* cage size, whether open top or individually ventilated (IVC), length of time in caging before disease induction (if applicable), age of introduction, objects included in cage, exercise opportunities, number of animals per cage, nesting materials, frequency of object rotation and/or cage cleaning rates.
v. *Disease model characteristics:* name of model and model description, time from disease induction to outcome measure.
vi. *Outcome measures:* described below

Information will be extracted from all articles determined to be relevant from the screening process.

For papers with no/limited description of the comparator (standard housed), we will make the assumption that the housing condition is equivalent to the minimum guidelines set for that country at that time. We will also assume that cage cleaning rates and housing density are the same for both control and enriched conditions unless otherwise specified.

OUTCOMES AND PRIORITIZATION.

For all outcomes listed below we will extract raw data. Since adjusted data is relatively rare, adjusted data will be extracted only when raw data is not reported.

Cardiovascular disease. In humans, psychological stress can impact cardiovascular disease at several stages and impact both the long-term development and acute incidence of cardiac events (Steptoe & Kivimaki, 2012). Chronic stress early in life can initiate the production of atherosclerotic lesions decades before the myocardial infarction (MI) (Steptoe & Kivimaki, 2012). Acute stress can also increase the likelihood of having a cardiac event via increases in acute inflammation, coagulation and vasoconstriction (Naghavi et al., 2003; Yeung et al., 1991). Both mice and rats are naturally resistant to the development of atherosclerosis and coronary heart disease (Liao, Huang, & Liu, 2015). Therefore, rodent studies of cardiovascular disease often have a different etiology to human disease (e.g. surgical models such as ligation of the left anterior descending coronary artery [LAD]) which are not relevant to our research question. Therefore, we will use rodent models of atherosclerosis and/or arteriosclerosis (genetic and diet-based manipulations to induce susceptibility) and assess intimal thickening as our first outcome measure. Intimal thickening will be extracted as a continuous measure with the mean for each treatment group and standard deviation. Secondly, we will extract information on spontaneous MI, which can occur in some genetic and diet-based rodent models of atherosclerosis (Braun et al., 2002; Van der Donckt et al., 2015). To assess spontaneous MI we will extract information on sudden death. For sudden death data we will extract odds ratios from cross tabulated counts of deaths if available (priority 1). If not, death percentages will be extracted (priority 2).

Major depression. In human studies, psychological stress is linked with more severe symptoms of major depression (Hammen, Davila, Brown, Ellicott, & Gitlin, 1992) (McGonagle & Kessler, 1990). The two cardinal symptoms of major depression are depressed mood and anhedonia (loss of interest or pleasure). Anhedonia is a commonly reported outcome in rodent depression models (via sucrose

preference tests). Therefore, we chose to include anhedonia as our first outcome measure of major depression. Depressed mood is described in the Diagnostic and Statistical Manual of Mental Disorders (DSM-5) "as indicated by… subjective report (e.g., feels sad, empty, hopeless)". Although depressed mood is more difficult to assess in rodents, hopelessness or despair is commonly measured in the form of Forced-Swim and Tail Suspension Tests. We will include measures of despair in our outcome data. Since is it not possible to use the entire DMS-5 clinical diagnostic criteria to determine depression in rodents, we will also include a common biomarker and reliable correlate of depression: hippocampal volume (Sheline, Sanghavi, Mintun, & Gado, 1999; Videbech & Ravnkilde, 2004). Traumatic and/or prolonged stress causes reductions in hippocampal volume, thus this outcome is highly relevant for this review (Kim, Pellman, & Kim, 2015). For each of these outcomes, we will extract information as continuous measures with the mean for each treatment group and standard deviation. Only if two or more studies report the same outcome test will it be compared in a meta-analysis. Examples for different tests of these outcomes can be found in **Table 4**.

Cancer. In humans, psychological stress impacts cancer progression via increasing tumor burden (Soung & Kim, 2015). Cancer outcomes will be extracted for any measurement of tumor burden. The primary outcome measure will be primary tumor volume at endpoint (priority 1). If this is not available, tumor length and width measurements will be extracted to calculate tumor volume (priority 2). We will also extract data on total number of tumors at endpoint or the number of animals which developed tumors (for spontaneous tumor development models). If available, we will also extract data on metastasis (total number and/or size of metastasis). All tumor burden outcomes will be extracted as continuous measures with the mean for each treatment group and standard deviation except for studies of spontaneous tumor development with data on tumor incidence. Tumor incidence outcomes will be extracted at end point as odds ratio from cross-tabulated tables of tumor incidence (priority 1) or as a percentage (priority 2) either as reported in the text or by digitizing graphical information. Only if two or more studies report the same type of tumor measurement will it be compared in a meta-analysis.

Viral infections. In humans, psychological stress increases the severity of viral infections by increasing viral load (Ironson et al., 2005), inflammatory cytokine levels and clinical symptoms (Cohen, Doyle, & Skoner, 1999; Cohen et al., 2012). Similarly, these outcomes are common in rodent models of viral infection and we will extract these three as our outcome measures (viral load, inflammatory cytokines and clinical symptoms). For pro-inflammatory cytokine levels, we will extract data on three cytokines: IL-1b, IL-6 and TNFα. Instead of the classical measures of clinical symptoms in humans, a robust way to determine viral disease progress in rodents is via weight loss (Matsuoka, Lamirande, & Subbarao, 2009). Although not a clinical symptom in humans, weight loss is a common symptom of disease severity in rodent infection models and an important indicator of disease progression. All three measures (viral load, inflammatory cytokine levels and weight loss) will be extracted as continuous measures with the mean for each treatment group and standard deviation.

Anxiety disorders. In humans, psychological stress increases the severity of anxiety disorder symptoms (Heim & Nemeroff, 2001). The diagnostic criteria for anxiety disorders in humans concerns persistent and excessive feelings of fear or anxiety which are assessed via discussions and questionnaires. Since this type of assessment cannot be performed in rodents, we will use common rodent behavioural tests of anxiety such as the widely used Open Field Test and Elevated Plus Maze (**Table 4**). Results from these behavioural tests will be extracted as continuous measures with the mean for each treatment group and standard deviation.

Asthma. In humans, psychological stress exacerbates asthma symptoms (Sandberg et al., 2000) (Wright, Rodriguez, & Cohen, 1998). To assess asthma severity in rodents, we will extract information on the total number of immune cells infiltrating the lung tissue or airspaces as well as total eosinophils. In humans, stress has also been shown to increase circulating levels of cytokines, most notably IL-4, IL-5 and IFN-γ (Marin, Chen, Munch, & Miller, 2009). We will also extract information on these cytokine levels when available. Data will be extracted as a continuous measure with the mean for each treatment group and standard deviation.

Stroke. In humans, psychological stress increases the severity of neurological and cognitive symptoms post stroke (Palmer & Glass, 2003). As such, we will extract information on functional evaluation post stroke and neurological function post stroke via sensorimotor tests and cognitive tests respectively (**Table 4**). If authors measure sensorimotor and cognitive function at multiple time points, we will extract outcomes at experimental endpoint (unless both the control and experimental groups have reached full recovery). We will also extract data on lesion volume which tends to correlate with symptom severity (Payabvash, Taleb, Benson, & McKinney, 2017). Although this is not a commonly reported outcome in humans, it is a direct measure of stroke severity in rodents so we believe it is important to include in our study. Only if two or more studies report the same stroke outcome measurement will it be compared in a meta-analysis. All stroke outcomes (including ordinal data [see **Table 4**]) will be extracted as continuous measures with the mean for each treatment group and standard deviation.

All-cause mortality. All-cause mortality data will be extracted as raw median lifespan data and hazard ratios as reported by the authors or by extracting the data from Kaplan–Meier curves. Data from Kaplan-Meier curves will be extracted in duplicate from graphical information using Web Plot Digitizer (https://automeris.io/WebPlotDigitizer/).

Outcome data to be extracted:

Table 4. Outcome measures to be extracted for each disease.

disease	outcome measures	tests	Measures
cardiovascular disease	intimal thickening	histological assessment	mm^2 or % of lumen filled
	myocardial infarction incidence	sudden death	cross tabulated tables or percentages
major depression	anhedonia	sucrose preference	% sucrose/ total drinking volume
	helplessness	Forced-Swim Tests	total time immobile
		Tail Suspension Tests	total time immobile
	hippocampal volume	volume measurements by MRI or histological sections	% of brain volume or total volume in mm^3
cancer	tumor burden	tumor volume	mm^3
		tumor weight	in milligrams
		number of animals with tumors	cross tabulated tables or percentages
		metastasis	number of metastasis or cell count

		number of tumors	tumor count
viral infections	viral load	viral load in relevant organ or blood (e.g. RT-PCR)	viral copy number
	cytokine levels	serum or relevant organ levels of IL-1b, IL-6 and TNFα	protein levels (pg/mL, ug/mL) or mRNA expression (ΔΔCT values)
	weight loss	rodent weight	% change from baseline or total weight lost
anxiety disorders	behavioural measures of anxiety	Elevated Plus Maze	% time spent in open arm
		Open Field Test	% time spent in center, total freezing time, rearing, defecation
		Light/Dark Box	% time in light side
		Social Interaction Test	% time spent interacting
asthma	total cell number	total cell number in lung tissue or airspaces	cell count
	eosinophils	total cell number in lung tissue or airspaces	cell count
	cytokine levels	serum levels of IL-4, IL-5 and IFN-γ	protein levels (pg/mL, ug/mL) or mRNA expression (ΔΔCT values)
stroke	infarct volume	histological assessment (e.g. TTC, H&E)	mm^3 or % of hemisphere
	motor and sensorimotor tests	composite scores (Benderson scale, Modified Neurological Severity Score, other scoring systems specified by the authors)	ordinal score data
		Accelerated Rotarod	mean fall score (RPM) or score relative to baseline or latency to fall
		Ledge Tapered Beam Test	foot faults or distance traveled
		Pasta Test	number of paw adjustments
		Limb Placement Test	ordinal score data
	cognitive impairment	Morris Water Maze	% time spent in target quadrant or path length
		Morris Water Maze (reversal learning)	% time spent in target quadrant or path length

Continuous measures: For continuous outcome measures, mean, standard deviation (or standard error) and sample size will be extracted to calculate and report standardized mean difference in the meta-analysis. For studies which do not report specific sample size (but give a range of sample sizes) the smallest possible sample size will be used to give the most conservative estimate of the 95% confidence interval. For studies that do not report the mean and standard deviation in the text (main text or supplemental), we will extract data from graphical information using Web Plot Digitizer (https://automeris.io/WebPlotDigitizer/). Studies that do not report how error bars were generated (standard deviation or standard error) will be excluded from the meta-analysis.

Extracting outcomes from studies with multiple experimental groups: It is common for biomedical rodent studies to have multiple experimental groups included in their studies, it is important to specify which groups will be extracted and included *a priori*. For this study, we are interested in comparing [diseased] control housing vs. [diseased] enriched housing. Accordingly, we will:

a) Exclude any rescue experiments (e.g. loss of function or gain of function).
b) Extract data on all genotypes or strains (other than those mentioned above).
c) Extract data on both sexes if sex is reported separately and include both in the meta-analysis separately unless: 1) the results are sufficiently similar and 2) enough studies report pooled sex data to warrant pooling results.
d) Flag studies reporting more than one time point (unless data extraction at end point is specified above). After extracting data from all studies with one time point, we will determine the time points most similar between studies and extract those for the meta-analysis.
e) Exclude control groups in which the intended disease model fails to generate disease in control animals.
f) Exclude data on drug treated groups or other intervention groups.

Risk of bias in individual studies: The risk of bias will be assessed using Systematic Review Protocols for Animal Intervention Studies (SYRCLE)'s risk of bias tool. Risk of bias will be conducted independently in duplicate by two reviewers, and the form will be pre-tested on the first 10 citations to ensure clarity and consistency between reviewers. This tool and checklist can be found here: https://bmcmedresmethodol.biomedcentral.com/articles/10.1186/1471-2288-14-43#Tab3

DATA.

Data synthesis. To summarize the findings, we will conduct a meta-analysis using R Studio. A meta-analysis will be conducted for each disease and each outcome individually using a random effect model. We will conduct a separate meta-analysis at the outcome level as described in **Table 4**. For each disease, a meta-analysis will only be performed if enough studies report similar outcome data (more than two studies). Heterogeneity will be assessed using I^2 and a value of >50% will be considered as being substantial heterogeneity (Higgins & Thompson, 2002).

Subgroup analysis. Subgroup analysis will be performed to assess potential sources of data heterogeneity based off the following: species (rat or mouse), sex, disease model, tests performed (as described in **Table 4**), time enrichment is introduced relative to disease induction or individual enrichment quality characteristics (e.g. wheel running studies alone).

Since there is considerable variation in the quality and consistency of rodent enrichment protocols, subgroup analyses will be performed to determine if these factors may account for some of the between study variance, if observed. Details of housing conditions will be extracted from each study included in the review and scored blind to author information and results. Enrichment will be classified based on nine parameters:

1) social opportunities
2) space
3) foraging opportunities
4) shelter/hiding opportunities
5) climbing/3-D movement
6) extra nesting material/sleeping places (e.g. hammock)
7) wheel(s)
8) exploration/novelty provision
9) chewing/gnawing opportunities

If 0-3 of these parameters are met enrichment will be classified as minimal enrichment; 3-6 as modest enrichment; and 6-9 as excellent enrichment. Next, we will assess if there are potential red flags that could reduce or abolish the effectiveness of the enrichment. Studies with red flags will be removed from the analysis to determine if they impact the effectiveness of the enrichment. The potential red flags are:

a) Enrichment that is supplied for less time than the disease can develop/remit in.
b) Novelty that is rotated very often (e.g. daily) that may induce a neophobic response.
c) Animals that are old (> middle aged) and are likely to be anhedonic or timid when faced with change.
d) Animals that are paired or group housed male mice, especially from strains known or reported to be aggressive.

Meta-bias. The potential for publication bias will be explored using funnel plots if ≥10 studies are available for any individual meta-analysis.

Confidence in Cumulative evidence. The strength of the body of evidence will be assessed using The Grading of Recommendations Assessment, Development and Evaluation (GRADE) guidelines (Guyatt et al., 2011) in all studies included in the meta-analysis.

DISCUSSION. This systematic review will synthesize current evidence surrounding the impact of housing conditions on biomedical disease outcomes and rodent health in biomedical research. The results of this review will aid in understanding the level of impact that standard housing conditions pose to the wellbeing of laboratory rodents.

Bailoo, J. D., Murphy, E., Boada-Saña, M., Varholick, J. A., Hintze, S., Baussière, C., . . . Würbel, H. (2018). Effects of Cage Enrichment on Behavior, Welfare and Outcome Variability in Female Mice. *Frontiers in Behavioral Neuroscience, 12*(232). doi:10.3389/fnbeh.2018.00232

Bechard, A., Meagher, R., & Mason, G. (2011). Environmental enrichment reduces the likelihood of alopecia in adult C57BL/6J mice. *Journal of the American Association for Laboratory Animal Science : JAALAS, 50*(2), 171-174.

Braun, A., Trigatti, B. L., Post, M. J., Sato, K., Simons, M., Edelberg, J. M., . . . Krieger, M. (2002). Loss of SR-BI expression leads to the early onset of occlusive atherosclerotic coronary artery disease, spontaneous myocardial infarctions, severe cardiac dysfunction, and premature death in apolipoprotein E-deficient mice. *Circ Res, 90*(3), 270-276. doi:10.1161/hh0302.104462

Canadian Council on Animal Care. (2019). CCAC guidelines: mice.

Chida, Y., Hamer, M., Wardle, J., & Steptoe, A. (2008). Do stress-related psychosocial factors contribute to cancer incidence and survival? *Nat Clin Pract Oncol, 5*(8), 466-475. doi:10.1038/ncponc1134

Chourbaji, S., Zacher, C., Sanchis-Segura, C., Dormann, C., Vollmayr, B., & Gass, P. (2005). Learned helplessness: validity and reliability of depressive-like states in mice. *Brain Res Brain Res Protoc, 16*(1-3), 70-78. doi:10.1016/j.brainresprot.2005.09.002

Cohen, S., Doyle, W. J., & Skoner, D. P. (1999). Psychological Stress, Cytokine Production, and Severity of Upper Respiratory Illness. *Psychosomatic Medicine, 61*(2), 175-180.

Cohen, S., Janicki-Deverts, D., Doyle, W. J., Miller, G. E., Frank, E., Rabin, B. S., & Turner, R. B. (2012). Chronic stress, glucocorticoid receptor resistance, inflammation, and disease risk. *Proceedings of the National Academy of Sciences, 109*(16), 5995-5999. doi:10.1073/pnas.1118355109

Cohen, S., Murphy, M. L. M., & Prather, A. A. (2019). Ten Surprising Facts About Stressful Life Events and Disease Risk. *Annual Review of Psychology, 70*(1), 577-597. doi:10.1146/annurev-psych-010418-102857

DSM-V. Anxiety Disorders *Diagnostic and Statistical Manual of Mental Disorders*.

DSM-V. Depressive Disorders *Diagnostic and Statistical Manual of Mental Disorders*.

Fureix, C., Walker, M., Harper, L., Reynolds, K., Saldivia-Woo, A., & Mason, G. (2016). Stereotypic behaviour in standard non-enriched cages is an alternative to depression-like responses in C57BL/6 mice. *Behavioural Brain Research, 305*, 186-190. doi:https://doi.org/10.1016/j.bbr.2016.02.005

Gaskill, B. N., Gordon, C. J., Pajor, E. A., Lucas, J. R., Davis, J. K., & Garner, J. P. (2013). Impact of nesting material on mouse body temperature and physiology. *Physiol Behav, 110-111*, 87-95. doi:10.1016/j.physbeh.2012.12.018

Gaskill, B. N., Pritchett-Corning, K. R., Gordon, C. J., Pajor, E. A., Lucas, J. R., Davis, J. K., & Garner, J. P. (2013). Energy reallocation to breeding performance through improved nest building in laboratory mice. *PLoS One, 8*(9), e74153. doi:10.1371/journal.pone.0074153

Guyatt, G., Oxman, A. D., Akl, E. A., Kunz, R., Vist, G., Brozek, J., . . . Schunemann, H. J. (2011). GRADE guidelines: 1. Introduction-GRADE evidence profiles and summary of findings tables. *J Clin Epidemiol, 64*(4), 383-394. doi:10.1016/j.jclinepi.2010.04.026

Hammen, C., Davila, J., Brown, G., Ellicott, A., & Gitlin, M. (1992). Psychiatric history and stress: Predictors of severity of unipolar depression [Press release]

Hankenson, F. C., Marx, J. O., Gordon, C. J., & David, J. M. (2018). Effects of Rodent Thermoregulation on Animal Models in the Research Environment. *Comp Med, 68*(6), 425-438. doi:10.30802/AALAS-CM-18-000049

Heim, C., & Nemeroff, C. B. (2001). The role of childhood trauma in the neurobiology of mood and anxiety disorders: preclinical and clinical studies. *Biological Psychiatry, 49*(12), 1023-1039. doi:https://doi.org/10.1016/S0006-3223(01)01157-X

Higgins, J. P. T., & Thompson, S. G. (2002). Quantifying heterogeneity in a meta-analysis. *Statistics in Medicine, 21*(11), 1539-1558. doi:10.1002/sim.1186

Horesh, N., Klomek, A. B., & Apter, A. (2008). Stressful life events and major depressive disorders. *Psychiatry Research, 160*(2), 192-199. doi:https://doi.org/10.1016/j.psychres.2007.06.008

Ironson, G., O'Cleirigh, C., Fletcher, M. A., Laurenceau, J. P., Balbin, E., Klimas, N., . . . Solomon, G. (2005). Psychosocial factors predict CD4 and viral load change in men and women with human immunodeficiency virus in the era of highly active antiretroviral treatment. *Psychosomatic Medicine, 67*(6), 1013-1021. doi:10.1097/01.psy.0000188569.58998.c8

Kim, E. J., Pellman, B., & Kim, J. J. (2015). Stress effects on the hippocampus: a critical review. *Learning & memory (Cold Spring Harbor, N.Y.), 22*(9), 411-416. doi:10.1101/lm.037291.114

Lampis, V., Maziade, M., & Battaglia, M. (2011). Animal Models of Human Anxiety Disorders: Reappraisal From a Developmental Psychopathology Vantage Point. *Pediatric Research, 69*(8), 77-84. doi:10.1203/PDR.0b013e318212b42e

Lawrence, E. M., Rogers, R. G., & Wadsworth, T. (2015). Happiness and longevity in the United States. *Social Science & Medicine, 145*, 115-119. doi:https://doi.org/10.1016/j.socscimed.2015.09.020

Li, M., Zhang, X. W., Hou, W. S., & Tang, Z. Y. (2015). Impact of depression on incident stroke: a meta-analysis. *Int J Cardiol, 180*, 103-110. doi:10.1016/j.ijcard.2014.11.198

Liao, J., Huang, W., & Liu, G. (2015). Animal models of coronary heart disease. *Journal of biomedical research, 30*(1), 3-10. doi:10.7555/JBR.30.20150051

Makowska, I. J., & Weary, D. M. (2016). The importance of burrowing, climbing and standing upright for laboratory rats. *R Soc Open Sci, 3*(6), 160136. doi:10.1098/rsos.160136

Marin, T. J., Chen, E., Munch, J. A., & Miller, G. E. (2009). Double-exposure to acute stress and chronic family stress is associated with immune changes in children with asthma. *Psychosomatic Medicine, 71*(4), 378-384. doi:10.1097/PSY.0b013e318199dbc3

Mason, G., Walker, M., Duggan, G., Roulston, N., & Van Slack, A. (2012). Negative affective states and their effects on morbidity, mortality and longevity.

Mason, G. J., Latham, N. (2004). Can't stop, won't stop: is stereotypy a reliable animal welfare indicator? *Animal Welfare*(13), S57-69.

Matsuoka, Y., Lamirande, E. W., & Subbarao, K. (2009). The mouse model for influenza. *Current protocols in microbiology, 13*(1), 15G. 13.11-15G. 13.30.

McGonagle, K. A., & Kessler, R. C. (1990). Chronic stress, acute stress, and depressive symptoms. *Am J Community Psychol, 18*(5), 681-706. doi:10.1007/bf00931237

Merriam-Webster Dictionary. (Ed.) Merriam-Webster. Merriam-Webster.com Dictionary: Merriam-Webster.

Miller, G. E., Chen, E., & Parker, K. J. (2011). Psychological stress in childhood and susceptibility to the chronic diseases of aging: moving toward a model of behavioral and biological mechanisms. *Psychol Bull, 137*(6), 959-997. doi:10.1037/a0024768

Moher, D., Shamseer, L., Clarke, M., Ghersi, D., Liberati, A., Petticrew, M., . . . Group, P.-P. (2015). Preferred reporting items for systematic review and meta-analysis protocols (PRISMA-P) 2015 statement. *Systematic Reviews, 4*(1), 1. doi:10.1186/2046-4053-4-1

Morris, J. K., Cook, D. G., & Shaper, A. G. (1994). Loss of employment and mortality. *BMJ (Clinical research ed.), 308*(6937), 1135-1139. doi:10.1136/bmj.308.6937.1135

Naghavi, M., Libby, P., Falk, E., Casscells, S. W., Litovsky, S., Rumberger, J., . . . Willerson, J. T. (2003). From vulnerable plaque to vulnerable patient: a call for new definitions and risk assessment strategies: Part I. *Circulation, 108*(14), 1664-1672. doi:10.1161/01.cir.0000087480.94275.97

National Research Council. (2011). In th (Ed.), *Guide for the Care and Use of Laboratory Animals*. Washington (DC).

Novaes, L. S., Dos Santos, N. B., Batalhote, R. F. P., Malta, M. B., Camarini, R., Scavone, C., & Munhoz, C. D. (2017). Environmental enrichment protects against stress-induced anxiety: Role of glucocorticoid receptor, ERK, and CREB signaling in the basolateral amygdala. *Neuropharmacology, 113*(Pt A), 457-466. doi:10.1016/j.neuropharm.2016.10.026

Olsson, I. A., & Dahlborn, K. (2002). Improving housing conditions for laboratory mice: a review of "environmental enrichment". *Lab Anim, 36*(3), 243-270. doi:10.1258/002367702320162379

Palmer, S., & Glass, T. A. (2003). Family Function and Stroke Recovery: A Review. *Rehabilitation Psychology, 48*(4), 255-265. doi:10.1037/0090-5550.48.4.255

Payabvash, S., Taleb, S., Benson, J. C., & McKinney, A. M. (2017). Acute Ischemic Stroke Infarct Topology: Association with Lesion Volume and Severity of Symptoms at Admission and Discharge. *American Journal of Neuroradiology, 38*(1), 58-63. doi:10.3174/ajnr.A4970

Rutters, F., Pilz, S., Koopman, A. D., Rauh, S. P., Te Velde, S. J., Stehouwer, C. D., . . . Dekker, J. M. (2014). The association between psychosocial stress and mortality is mediated by lifestyle and chronic diseases: the Hoorn Study. *Soc Sci Med, 118*, 166-172. doi:10.1016/j.socscimed.2014.08.009

Sandberg, S., Paton, J. Y., Ahola, S., McCann, D. C., McGuinness, D., Hillary, C. R., & Oja, H. (2000). The role of acute and chronic stress in asthma attacks in children. *The Lancet, 356*(9234), 982-987. doi:https://doi.org/10.1016/S0140-6736(00)02715-X

Segerstrom, S. C., & Miller, G. E. (2004). Psychological stress and the human immune system: a meta-analytic study of 30 years of inquiry. *Psychol Bull, 130*(4), 601-630. doi:10.1037/0033-2909.130.4.601

Seong, H. H., Park, J. M., & Kim, Y. J. (2018). Antidepressive Effects of Environmental Enrichment in Chronic Stress-Induced Depression in Rats. *Biol Res Nurs, 20*(1), 40-48. doi:10.1177/1099800417730400

Sheline, Y. I., Sanghavi, M., Mintun, M. A., & Gado, M. H. (1999). Depression duration but not age predicts hippocampal volume loss in medically healthy women with recurrent major depression. *J Neurosci, 19*(12), 5034-5043.

Sherwin, C. M. (1996). Laboratory mice persist in gaining access to resources: a method of assessing the importance of environmental features. *Applied Animal Behaviour Science, 48*(3), 203-213. doi:https://doi.org/10.1016/0168-1591(96)01027-1

Soung, N. K., & Kim, B. Y. (2015). Psychological stress and cancer. *Journal of Analytical Science and Technology, 6*(1), 30. doi:10.1186/s40543-015-0070-5

Steptoe, A., & Kivimaki, M. (2012). Stress and cardiovascular disease. *Nat Rev Cardiol, 9*(6), 360-370. doi:10.1038/nrcardio.2012.45

Stringhini, S., Berkman, L., Dugravot, A., Ferrie, J. E., Marmot, M., Kivimaki, M., & Singh-Manoux, A. (2012). Socioeconomic status, structural and functional measures of social support, and mortality: The British Whitehall II Cohort Study, 1985-2009. *Am J Epidemiol, 175*(12), 1275-1283. doi:10.1093/aje/kwr461

Van der Donckt, C., Van Herck, J. L., Schrijvers, D. M., Vanhoutte, G., Verhoye, M., Blockx, I., . . . De Meyer, G. R. (2015). Elastin fragmentation in atherosclerotic mice leads to intraplaque neovascularization, plaque rupture, myocardial infarction, stroke, and sudden death. *Eur Heart J, 36*(17), 1049-1058. doi:10.1093/eurheartj/ehu041

van der Harst, J. E., Fermont, P. C. J., Bilstra, A. E., & Spruijt, B. M. (2003). Access to enriched housing is rewarding to rats as reflected by their anticipatory behaviour. *Animal Behaviour, 66*(3), 493-504. doi:https://doi.org/10.1006/anbe.2003.2201

Videbech, P., & Ravnkilde, B. (2004). Hippocampal volume and depression: a meta-analysis of MRI studies. *Am J Psychiatry, 161*(11), 1957-1966. doi:10.1176/appi.ajp.161.11.1957

Walker, M. D., & Mason, G. (2011). Female C57BL/6 mice show consistent individual differences in spontaneous interaction with environmental enrichment that are predicted by neophobia. *Behavioural Brain Research, 224*(1), 207-212. doi:https://doi.org/10.1016/j.bbr.2011.06.003

Wright, R. J., Rodriguez, M., & Cohen, S. (1998). Review of psychosocial stress and asthma: an integrated biopsychosocial approach. *Thorax, 53*(12), 1066-1074. doi:10.1136/thx.53.12.1066

Yeung, A. C., Vekshtein, V. I., Krantz, D. S., Vita, J. A., Ryan, T. J., Jr., Ganz, P., & Selwyn, A. P. (1991). The effect of atherosclerosis on the vasomotor response of coronary arteries to mental stress. *N Engl J Med, 325*(22), 1551-1556. doi:10.1056/nejm199111283252205
