# Supplementary material for: Conventional laboratory housing increases morbidity and mortality in research rodents: results of a meta-analysis
Source: BMC Biol. 2022 Jan 13;20:15. doi: 10.1186/s12915-021-01184-0 (PMC8756709; doi:10.1186/s12915-021-01184-0)
Supplement: Supplementary file 4 — Additional file 4. Protocol amendments. [file 12915_2021_1184_MOESM4_ESM.pdf]

| Change | Reason | Date |
| --- | --- | --- |
| The protocol was only deposited at one site (University of Guelph Atrium) | One open access site seemed sufficient for *a priori* documentation. | May 22 2020 |
| An additional question was added to the title/abstract screening process: "could this article be potentially relevant to the review?" | The literature search generated > 10,000 references. This necessitated another data screening stage: rapid title screening for potentially relevance. | May 26 2020 |
| We did not extract data on whether cages were open top or individually ventilated (IVC) | These data were very rarely reported so were not extracted. | June 5 2020 |
| Multiple subgroup analysis was exchanged for meta-regression analysis | We realised we could successfully perform a meta-regression, rather than analyze each subgroup individually, so increasing power and allowing analysis of some interactions. | January 16 2021 |
| Analysis was categorized by 'resource type' rather than tallying all resources | 'Resource' category was modified based on a recognition that resources have qualitatively different effects (e.g. exercise is qualitatively different from nesting). These categories were also pragmatic: based on available data on how EH actually differed from CH. Thirdly, precision was also an issue (e.g. was a 'structure' used for climbing, or to nest within, or not at all?) making some resources difficult to assess. | February 6 2021 |
| Studies reporting tumor incidence in 2x2 tables were not included in the analysis | Odds ratios were not compatible with other (standardized mean difference) results. | February 7 2021 |
| Added analysis of coefficient of variation | Further reading suggested the value of assessing whether coefficients of variation differed between conventional and enriched conditions. | May 22 2021 |
