# Supplementary material for: Conventional laboratory housing increases morbidity and mortality in research rodents: results of a meta-analysis
Source: BMC Biol. 2022 Jan 13;20:15. doi: 10.1186/s12915-021-01184-0 (PMC8756709; doi:10.1186/s12915-021-01184-0)
Supplement: Supplementary file 6 — Additional file 6. Database search strategy. [file 12915_2021_1184_MOESM6_ESM.pdf]

Database search (May 24, 2020) developed in MEDLINE (Ovid).

	Search	Results
#1	exp Murinae/	
#2	(mice OR mouse OR Mus OR rodent* OR murine OR rat OR rats).ti,kw,ab.	
#3	#1 OR #2	3355230
#4	Housing, Animal/	
#5	((cage OR caging OR caged OR cages OR environment*) adj3 (enrich* OR naturalistic)).ti,kw,ab.	
#6	(“voluntary wheel running" or "running wheel" or "wheel running" or "running disk" or “physical activity”).ti,kw,ab.	
#7	#4 OR #5 OR #6	123309
#8	Cardiovascular Diseases/	
#9	("cardiovascular disease*" or "coronary artery disease" or "myocardial infarct*" or "coronary heart disease" or "atherosclero*" or "arteriosclero* myocardial ischemia" or "ischemic heart disease" or "coronary heart disease" or "APOE" or "Apolipoprotein E" or "intimal thickening" or "lumen stenosis" or "lumen occlusion*" or "atherogenic diet*" or "coronary lesion*").ti,kw,ab.	
#10	#8 OR #9	655346
#11	Depressive Disorder/ or Depressive Disorder, Major/	
#12	("model of depression" or "major depression" or "major depressive disorder" or "depressive disorder" or "forced-swim* test" or "forced swim* test" or "anhedonia" or "sucrose preference" or "social defeat stress" or "tail suspension test" or "chronic mild stress" or "learned helplessness" or "olfactory bulbectomy" or "maternal separation" or "chronic restraint stress").ti,ab,kw.	
#13	#11 OR #12	131770
#14	exp Neoplasms/	
#15	(carcino* or cancer or malignant or tumor or tumour).ti,kw,ab.	
#16	#14 OR #15	4150555
#17	exp Viruses/ or exp Virus/ or Virus Diseases/	
#18	("viral infection" or virus or "immunodeficiency virus" or HIV or "infectious disease" or "respiratory disease" or "upper respiratory disease" or influenza).ti,kw,ab.	
#19	#17 OR #18	1238351
#20	Asthma/	
#21	(OVA or asthma or asthmatic or "house dust mite" or "papain" or "atopic" or "allergic lung inflammation").ti,ab,kw.	
#22	#20 OR #21	221340
#23	Anxiety/ or Anxiety Disorders/	
#24	("models of anxiety" or "anxiety disorder*" or "anxiety" or "anxious" or "general anxiety" or "material separation" or anxiogenic).ti,kw,ab.	
#25	#23 OR #24	218570
#26	Stroke/	

#27	("stroke" or "cerebrovascular disease" or "cerebrovascular disorders" or "cerebral infarct" or "ischemic stroke" or "intracranial hemorrhage" or "intracranial artery disease" or "middle cerebral artery occlusion" or "MCAO").ti,kw,ab.	
#28	#26 OR #27	274870
#29	exp Aging/	
#30	(longevity or mortality or survivorship or "survival rate" or survival).ti,kw,ab.	
#31	#29 OR #30	1774970
#32	#10 OR #13 OR #16 OR #19 OR #22 OR #25 OR #28 OR #31	7453822
#33	#3 AND #7 AND #32	2692

The search strategy was developed using medical subject headings (MeSH) and text words relating to our population (mouse and rat), intervention (environmental enrichment) and outcomes (diseases); and designed in MEDLINE (Ovid) before being adapted for other databases. ti= title, kw= author keywords, ab= abstract exp= explode.

Searches adapted for other databases.

<u>CAB direct (results: 1,686), Web of Science (results: 5,879) & SCOPUS (results: 5,857)</u>

murinae OR mice OR mouse OR Mus OR rodent* OR murine OR rat OR rats

AND

cage OR caging OR caged OR cages OR "enrich* environment*" OR "environmental enrichment" OR "naturalistic environment" OR “voluntary wheel running" or "running wheel" or "wheel running" or "running disk" or “physical activity”

AND

"cardiovascular disease*" or "coronary artery disease" or "myocardial infarct*" or "coronary heart disease" or "atherosclero*" or "arteriosclero* myocardial ischemia" or "ischemic heart disease" or "coronary heart disease" or "APOE" or "Apolipoprotein E" or "intimal thickening" or "lumen stenosis" or "lumen occlusion*" or "atherogenic diet*" or "coronary lesion*" or "model of depression" or "major depression" or "major depressive disorder" or "depressive disorder" or "forced-swim* test" or "forced swim* test" or "anhedonia" or "sucrose preference" or "social defeat stress" or "tail suspension test" or "chronic mild stress" or "learned helplessness" or "olfactory bulbectomy" or "maternal separation" or "chronic restraint stress" OR carcino* or cancer or malignant or tumor or tumour OR "viral infection" or virus or "immunodeficiency virus" or HIV or "infectious disease" or "respiratory disease" or "upper respiratory disease" or influenza OR OVA or asthma or asthmatic or "house dust mite" or "papain" or "atopic" or "allergic lung inflammation" OR "models of anxiety" or "anxiety disorder*" or "anxiety" or "anxious" or "general anxiety" or "material separation" or anxiogenic OR stroke or "cerebrovascular disease" or "cerebrovascular disorders" or "cerebral infarct" or "ischemic

stroke" or "intracranial hemorrhage" or "intracranial artery disease" or "middle cerebral artery occlusion" or "MCAO" OR longevity or mortality or survivorship or "survival rate" or survival

ProQuest (limit thesis and dissertations) (results: 276)

ab(murinae OR mice OR mouse OR Mus OR rodent* OR murine OR rat OR rats) AND ab("environmental enrichment" or "enriched environment" or "voluntary wheel running")
