# Supplementary material for: Conventional laboratory housing increases morbidity and mortality in research rodents: results of a meta-analysis
Source: BMC Biol. 2022 Jan 13;20:15. doi: 10.1186/s12915-021-01184-0 (PMC8756709; doi:10.1186/s12915-021-01184-0)
Supplement: Supplementary file 7 — Additional file 7. Title/abstract screening and full text eligibility questions. [file 12915_2021_1184_MOESM7_ESM.pdf]

Title/abstract screening. The following questions were used for title/abstract screening:

1. Could this article be potentially relevant to this review?
2. Is the title and/or abstract available in English?
3. Does the title and/or abstract describe a primary *in vivo* research trial?
4. Does the title and/or abstract use laboratory mice or rats for their study?
5. Does the title and/or abstract use environmental 'enrichment' as an intervention?
6. Does the title and/or abstract report the use of one of the disease models of interest and/or study survival/mortality?

All articles receiving a 'no' (from both reviewers) for any of the above questions were excluded. Conflicts were resolved via consensus. If consensus could not be reached, a third reviewer was consulted.

Full text eligibility. The following questions were used to assess full text eligibility:

1. Is the full text available?
2. Is the article available in English and over 500 words?
3. Is it a primary *in vivo* research trial?
4. Do the researchers use laboratory mice or rats in the study?
5. Does the study use appropriate environmental 'enrichment' (as described in the exclusion criteria) as an intervention?
6. Does the study use appropriate (as described in the exclusion criteria) conventional housing as a comparator?
7. Does the study include one of the disease models of interest (i.e. cardiovascular disease, major depression, cancer, viral infection, asthma, anxiety disorders, stroke and/or a mortality study)?
8. Does the study measure any of the relevant outcomes (measures) or report survival/mortality data?

All articles receiving a 'no' (from both reviewers) for any of the above questions were excluded. Conflicts were resolved via consensus. If consensus could not be reached, a third reviewer was consulted.
