# Supplementary material for: Conventional laboratory housing increases morbidity and mortality in research rodents: results of a meta-analysis
Source: BMC Biol. 2022 Jan 13;20:15. doi: 10.1186/s12915-021-01184-0 (PMC8756709; doi:10.1186/s12915-021-01184-0)
Supplement: Supplementary file 8 — Additional file 8. Study level data collected. An article may contain multiple studies (where each study contains one set of animals under the same conditions described here). [file 12915_2021_1184_MOESM8_ESM.pdf]

Animal Characteristics	Housing Characteristics	Disease Characteristics
- species<br>- sex<br>- strain<br>- genotype (if transgenic)	- description of conventional housing<br>- description of enriched housing<br>- social status<br>- age animals were differentially housed<br>- length of housing exposure<br>- object rotation/replacement frequency<br>- if enrichment was removed prior to disease induction and for how long<br>- “red flags” (see methods)	- disease model (e.g. transgenic, surgical, dietary etc.)<br>- measures reported<br>- unit of measure
