# Supplementary material for: Conventional laboratory housing increases morbidity and mortality in research rodents: results of a meta-analysis
Source: BMC Biol. 2022 Jan 13;20:15. doi: 10.1186/s12915-021-01184-0 (PMC8756709; doi:10.1186/s12915-021-01184-0)
Supplement: Supplementary file 9 — Additional file 9. Results from random-effects meta-analyses rerun without study weights (cf. Figs. 3-9 which include study weights), comparing disease outcomes and mortality in conventionally housed and environmentally enriched animals. [file 12915_2021_1184_MOESM9_ESM.pdf]

**cancer**	SMD = 0.79, 95%CI = 0.62-0.97, z = 8.87, p < 0.0001
**cardiovascular disease**	SMD = 0.91, 95%CI = 0.56-1.27 , z = 5.04, p < 0.0001
**stroke**	SMD = 0.92, 95%CI = 0.64-1.20, z = 6.39, p < 0.0001
**anxiety**	SMD = 1.06, 95%CI = 0.71-1.42, z = 5.89, p < 0.0001
**depression**	SMD = 1.67, 95%CI = 1.36-1.98, z = 10.70, p < 0.0001
**hazard ratios**	hazard ratio = 1.61, 95%CI = 1.31-1.97, z = 4.59, p < 0.0001
**median survival**	ROM = 0.92, 95%CI = 0.89-0.96, z = -4.36, p < 0.0001
