# Supplementary material for: Conventional laboratory housing increases morbidity and mortality in research rodents: results of a meta-analysis
Source: BMC Biol. 2022 Jan 13;20:15. doi: 10.1186/s12915-021-01184-0 (PMC8756709; doi:10.1186/s12915-021-01184-0)
Supplement: Supplementary file 10 — Additional file 10. Results from a random-effects meta-regression rerun without study weights (cf. Table 1 which includes study weights), investigating potential moderators of housing effects on stress-sensitive disease (standardized mean differences). Bold p values are significant at p < 0.05. [file 12915_2021_1184_MOESM10_ESM.pdf]

	**Test Statistic**	**p**
**species**	$F_{1,154}$ = 0.0991	0.7534
**sex**	$F_{2,154}$ = 0.0190	0.9812
**social status**	$F_{2,154}$ = 0.5127	0.5999
**disease**	$F_{4,154}$ = 1.6360	0.1680
**measure: infarct volume versus all others**	$F_{1,154}$ = 21.9755	< 0.0001
**species*sex**	$F_{2,154}$ = 1.3099	0.2728
**species*social status**	$F_{2,154}$ = 0.2547	0.7755
**sex*social status**	$F_{4,154}$ = 0.5026	0.7339
**resource category**	$F_{3,139}$ = 1.2245	0.3032
**resource category*species**	$F_{3,139}$ = 1.1497	0.3314
**after removal of ‘red flags’**		
**species**	$F_{1,91}$ = 0.0465	0.8297
**sex**	$F_{2,91}$ = 0.0891	0.9148
**social status**	$F_{2,91}$ = 0.2655	0.7674
**disease**	$F_{4,91}$ = 2.4741	**0.0498**
**measure: infarct volume versus all others**	$F_{1,91}$ = 16.3696	**0.0001**
**species*sex**	$F_{2,91}$ = 0.6379	0.5307
**species*social status**	$F_{2,91}$ = 0.6279	0.5360
**sex*social status**	$F_{4,91}$ = 0.1280	0.9719
**resource category**	$F_{3,83}$ = 2.8122	**0.0443**
**resource category*species**	$F_{3,83}$ = 1.0025	0.3960
