# Supplementary material for: Conventional laboratory housing increases morbidity and mortality in research rodents: results of a meta-analysis
Source: BMC Biol. 2022 Jan 13;20:15. doi: 10.1186/s12915-021-01184-0 (PMC8756709; doi:10.1186/s12915-021-01184-0)
Supplement: Supplementary file 11 — Additional file 11. Results from a random-effects meta-regression rerun without study weights (cf. Table 2 which includes study weights), investigating potential moderators of housing effects on hazard ratio. [file 12915_2021_1184_MOESM11_ESM.pdf]

	Test Statistic	p
**species**	$F_{1,27} = 0.8281$	0.3709
**sex**	$F_{2,27} = 0.3501$	0.7078
**social status**	$F_{2,27} = 1.4566$	0.2508
**spontaneous or induced disease**	$F_{1,27} = 0.0454$	0.8329
**species*sex**	$F_{1,27} = 0.3153$	0.5791
**species*social status**	$F_{1,27} = 0.2354$	0.6315
**sex*social status**	$F_{2,27} = 0.5335$	0.5926
**resource category**	$F_{1,26} = 0.0101$	0.9208
**after removal of ‘red flags’**		
**species**	$F_{1,18} = 0.8237$	0.3761
**sex**	$F_{2,18} = 0.2132$	0.8100
**social status**	$F_{2,18} = 0.1408$	0.8696
**spontaneous or induced disease**	$F_{1,18} = 0.7598$	0.3949
**species*sex**	$F_{1,18} = 0.5018$	0.4878
**species*social status**	$F_{1,18} = 0.3303$	0.5726
**sex*social status**	$F_{2,18} = 0.5802$	0.5699
**resource category**	$F_{1,17} = 0.1804$	0.6763
