# Supplementary material for: Conventional laboratory housing increases morbidity and mortality in research rodents: results of a meta-analysis
Source: BMC Biol. 2022 Jan 13;20:15. doi: 10.1186/s12915-021-01184-0 (PMC8756709; doi:10.1186/s12915-021-01184-0)
Supplement: Supplementary file 12 — Additional file 12. R syntax used for data analysis. [file 12915_2021_1184_MOESM12_ESM.pdf]

```
### random-effects meta-analysis per disease and subgroup

# load libraries

library(metafor)
library(svglite)

# load CSV file of dataset

dataset <- read.csv(file.choose(),header=T)

# create a random-effects model

diseasemodel <- rma(y, v, data=dataset)

# extract relevant summary statistics

summary(diseasemodel)

# create a random-effects model for each subset

subset.a <- rma(y, v, subset=(outcome.reported=="outcome measurement a"),
data=dataset)
subset.b <- rma(y, v, subset=(outcome.reported=="outcome measurement b"),
data=dataset)
subset.c <- rma(y, v, subset=(outcome.reported=="outcome measurement c"),
data=dataset)

# extract relevant summary statistics for each subset

summary(subset.a)
summary(subset.b)
summary(subset.c)


# create a forest plot

forest(diseasemodel)

# add summary polygon for each subset

addpoly(subset.b, row=25)
addpoly(subset.c, row=26)

# export plot as svg

svglite(file="figurex.svg")
forest(diseasemodel)
addpoly(subset.a, row=30)
addpoly(subset.b, row=25)
```

```
addpoly(subset.c, row=26)
dev.off()

# test for differences in subgroup effect estimate

sub <- rma(y, v, mods = ~ measure, data=dataset)


### run random-effects model without study weights

diseasemodel <- rma(y, v, data=dataset, weighted=FALSE)


### random-effects meta-analysis of merged disease dataset

# load CSV file of dataset

dataset <- read.csv(file.choose(),header=T)

# create a random-effects model

overall <- rma(y, v, data=dataset)

# generate a funnel plot to visualize publication bias

funnel(overall, col=ifelse(dataset$y > 3, "blue", "black"))

# Rank Correlation Test for Funnel Plot Asymmetry

ranktest(overall$y, overall$v, digits=3, exact=FALSE)

# load new dataset ommitting publication bias

dataset <- read.csv(file.choose(),header=T)

# create a random-effects model

overall <- rma(y, v, data=dataset)

# generate a new funnel plot to visualize absence of publication bias

funnel(overall)

# add moderators to random-effects meta-analysis

overall <- rma(y, v, mods = ~ sex + species + social + disease + infarct +
sex*social + species*social + species*sex + eetype + eetype*species, data=dataset,
test="knha")
```

```
# test moderators using ANOVA

sex <- anova(overall, btt = 2:3)
species <- anova(overall, btt = 4)

# test coefficient of variation ratio

datvar <- escalc(measure = "CVR", m1i=mean.c, m2i=mean.e, sd1i=sd.c, sd2i=sd.e,
n1i=n.c, n2i=n.e, add.measure=TRUE, var.names= c("covar", "covarv"), data=dataset)
datvarmod <- rma(covar, covarv, data=datvar)



### random-effects meta-analysis of hazard ratios

# load CSV file of dataset

dataset <- read.csv(file.choose(),header=T)

# create a random-effects model

hr <- rma.mv(ylog, sei^2, data=dataset)

# create a random-effects model for each subset

subset.a <- rma(ylog, sei^2, subset=(outcome.reported=="outcome measurement a"),
data=dataset)
subset.b <- rma(ylog, sei^2, subset=(outcome.reported=="outcome measurement a"),
data=dataset)

# create a forest plot

forest(hr, atransf=exp, at=log(c(.05, .25, 1, 4, 20)))

# add summary polygon for each subset

addpoly(subset.a, atransf=exp, row=30)
addpoly(subset.b, atransf=exp, row=25)

# export plot as svg

svglite(file="figurex.svg")
forest(hr, atransf=exp, at=log(c(.05, .25, 1, 4, 20)))
addpoly(subset.a, row=30)
addpoly(subset.b, row=25)
addpoly(subset.c, row=26)
dev.off()

# assess HR studies for publication bias
```

```
funnel(hr, atransf=exp, at=log(c(.05, .25, 1, 4, 20)))
ranktest(overall$ylog, overall$sei^2, digits=3, exact=FALSE)


# add moderators to hazard ratio random-effects meta-analysis

hr <- rma(ylog, sei^2, mods = ~ sex + species + social + treatment + sex*social +
species*social + species*sex + eetype, data=dataset, test="knha")

# test moderators using ANOVA

sex <- anova(hr, btt = 2:3)
species <- anova(hr, btt = 4)



### random-effects meta-analysis of median survival

# calculate log ratio of means

medi <- escalc(measure = "ROM", m1i=median.e, m2i=median.c, n1i=n.e, n2i=n.c,
sd1i=sd.e, sd2i=sd.c, data = dataset)

medimod <- rma(y, v, data=medi)
```
