# Supplementary material for: Conventional laboratory housing increases morbidity and mortality in research rodents: results of a meta-analysis
Source: BMC Biol. 2022 Jan 13;20:15. doi: 10.1186/s12915-021-01184-0 (PMC8756709; doi:10.1186/s12915-021-01184-0)
Supplement: Supplementary file 13 — Additional file 13. Studies excluded at full text article screening. [file 12915_2021_1184_MOESM13_ESM.pdf]

Full text articles excluded

Full text not available.

Antonelli, J.,Jones, L.,Thomas, J. A.,Masko, E.,Lloyd, J.,Poulton, S.,Phillips, T.,Tewari, A.,Febbo, P.,Pollak, M.,Dewhirst, M.,Freedland, S. J.. EFFECT OF VOLUNTARY WHEEL RUNNING ON GROWTH OF PROSTATE CANCER IN IMMUNOCOMPROMISED AND IMMUNOCOMPETENT MOUSE MODELS. *Journal of Urology.* 2010. 183:E37-E37:

Asoh, T.,Takeuchi, Y.,Tsuji, H.. Effect of voluntary exercise on resistance to trauma in rats. *Circulatory Shock.* 1986. 20:259-267:

Basu, S.,Combe, K.,Nachat-Kappes, R.,Farges, M. C.,Rossary, A.,Goncalves-Mendes, N.,Vasson, M. P.. Impact of enriched environment on tumor growth and adipokines secretion in a mouse model of mammary cancer. *Journal of Clinical Oncology.* 2013. 31:#pages#:

Borre, Y.,Jongbloets, B. C.,Mendes, J. M.,Olivier, B.,Oosting, R. S.. Size does matter: environmental enrichment rescues cognitive and synaptic deficits following olfactory bulbectomy in mice. *European Neuropsychopharmacology.* 2012. 22:S46-S46:

Cohen, L. A.,Kendall, M. E.,Meschter, C.,Epstein, M. A.,Reinhardt, J.,Zang, E.. Inhibition of rat mammary tumorigenesis by voluntary exercise. *In Vivo.* 1993. 7:151-158:

Colbert, L. H.,Westerlind, K. C.,Perkins, S. N.,Wimbrow, H.,Hursting, S. D.. Treadmill and running wheel exercise are not beneficial to survival in a p53-deficient Wnt-I transgenic mouse model of breast cancer. *Cancer Epidemiology Biomarkers & Prevention.* 2003. 12:1302S-1302S:

Colon-Echevarria, C.,Cruz, M. L.,Doreste, R.,Torres-Reveron, A.,Appleyard, C. B.. The Effect of Maternal Separation and Early Environmental Enrichment on the Rat Colon. *Faseb Journal.* 2016. 30:#pages#:

Comassio, P.,Merlo, S.,Fonseca, C.,Nakayama, A. B.,Lemos, J. I.,Moreira, J.. Enriched environment recovers modifications of the glutamatergic system in the hippocampus of stressed young rats. *Glia.* 2017. 65:E247-E248:

Fuentes, I.,Pierce, A.,Eller-Smith, O.,Christianson, J.. Voluntary wheel running can improve increased urogenital sensitivity and function resulting from neonatal maternal separation in male mice. *Journal of Pain.* 2017. 18:S10-S10:

Hao, C.,Wang, J.,Liao, W.. The influence of "preservation from both physique and spirit" on cognitive function, SYN, MAP- 2 and pyramidal cells in rats. *Chinese Journal of Rehabilitation Medicine.* 2017. 32:384-390:

Huai, Y.,Yang, Y.,Jia, Z.. The effects of learning on nestin and nerve growth factor expressions in peri-ischemic cortex of rats after unilateral local cerebral infarction. *Chinese Journal of Rehabilitation Medicine.* 2009. 24:977-980:

Ikuyama, T.,Arao, T.,Osanai, H.. Repressive effect of physical exercise on the incidence of hepatoma due to benzidine administration (Japanese). *Bulletin of the Physical Fitness Research Institute.* 1976. No. 35:1-15:

Irie, M.,Nagata, S.. Effects of the lesions of the suprachiasmatic nuclei and light-dark phase inversed exercise on asthmatic responses in rats. *Advances in the Prevention of Occupational Respiratory Diseases.* 1998. 1153:764-768:

Johansson, B. B.,Komitova, M.,Perfilieva, E.,Mattsson, B.,Eriksson, P.. Postischemic housing in an enriched environment influences hippocampal progenitor cell differentiation after focal cortical ischemia. *Maturation Phenomenon in Cerebral Ischemia V.* 2004. #volume#:297-308:

Komitova, M.,Perfilieva, E.,Mattsson, B.,Eriksson, P. S.,Johansson, B. B.. Effects of cortical ischemia and postischemic environmental enrichment on hippocampal cell genesis and differentiation in the adult rat. *Journal of Cerebral Blood Flow and Metabolism.* 2002. 22:852-860:

Komitova, M.,Perfilieva, E.,Mattsson, B.,Eriksson, P. S.,Johansson, B. B.. Enriched environment after focal cortical ischemia enhances the generation of astroglia and NG2 positive polydendrocytes in adult rat neocortex. *Experimental Neurology.* 2006. 199:113-121:

Komitova, Mila. Neurogenesis and gliogenesis after focal brain ischemia: Modulation by enriched environment and exercise. *#journal#.* 2005. C821570:103:English

Lang, J.,Santaolalla, R.,Dheer, R.,Davies, J.,Phillips, M.,Grant, J.,Zaias, J.,Abreu, M.. The Effects of Environmental Enrichment, Social Housing and Gender on Intestinal Tumorigenesis in a Murine Model of Colon Cancer. *Inflammatory Bowel Diseases.* 2016. 22:S53-S53:

Laufs, U.,Gertz, K.,Lindauer, U.,Schrock, H.,Kuschinsky, W.,Dirnagl, U.,Bohm, M.,Endres, M.. Physical activity protects from stroke in mice via eNOS-dependent mechanisms. *Circulation.* 2003. 108:603-603:

Li, L.,Hu, X.,Zhang, L.. Effects of exercise training on neural function and cell proliferation and apoptosis in rats with acute cerebral infarction. *Chinese Journal of Rehabilitation Medicine.* 2013. 28:528-532:

Luo, J.,Hu, X.,Zhang, L.. Effects of stromal-derived factor-1α/chemokine receptor 4 pathways in physical exercise on migration of endogenous neural stem cells from subventricular zone in rats with focal cerebral infarction. *Chinese Journal of Rehabilitation Medicine.* 2014. 29:299-305:

Markt, P.,Nussbaumer, M.,Touma, C.,Landgraf, R.. Effects of enriched environment on a mouse model of extremes in trait anxiety. *Pharmacopsychiatry.* 2009. 42:230-230:

Meissner, M.,Lombardo, E.,van Dijk, T. H.,Havinga, R.,Tietge, U. J. F.,Boverhof, R.,Boer, T.,Bijsterveld, K.,Kuipers, F.,Groen, A. K.. VOLUNTARY WHEEL RUNNING BENEFICIALLY AFFECTS CHOLESTEROL TURNOVER AND ATHEROSCLEROSIS IN HYPERCHOLESTEROLEMIC MICE. *Atherosclerosis Supplements.* 2010. 11:114-114:

Nomura, M.,Okamura, K.. CHANGES OF CATECHOLAMINE CONTENT AND WHEEL RUNNING OF SPONTANEOUSLY HYPERTENSIVE RAT WITH OR WITHOUT STROKE. *Neurochemical Research.* 1986. 11:1744-1745:

Nonaka, M.,Ueno, S.,Uezono, Y.. Cardio-oncology — elucidation of the mechanism of cardiac dysfunction caused by cancer therapy and cancer cachexia. *Folia Pharmacologica Japonica.* 2020. 155:165-170:

O'Neal, H. A.,Van Hoomissen, J. D.,Holmes, P. V.,Bunnell, B. N.,Dishman, R. K.. EFFECT OF CHRONIC ACTIVITY WHEEL RUNNING ON MALE COPULATORY BEHAVIOR AND NEUROPEPTIDE Y GENE EXPRESSION IN RAT LOCUS COERULEUS AFTER OLFACTORY BULBECTOMY. *Medicine and Science in Sports and Exercise.* 2001. 33:S178-S178:

Osanai, H.,Ikuyama, T.. An experimental study on the suppression of hepatoma in mice. *Bulletin of the Physical Fitness Research Institute.* 1974. No. 29:1-22:

Pence, B. D.,Ryerson, M. R.,Bravo-Cruz, A. G.,Woods, J. A.,Shisler, J. L.. Voluntary Wheel Running and Response to Vaccinia Virus Infection and Inoculation in Mice. *Medicine and Science in Sports and Exercise.* 2017. 49:196-197:

Peng, Y. J.,Jian, X. H.,Liu, L. H.,Tong, J. B.,Lei, D. L.. Influence of environmental enrichment on hippocampal synapses in adolescent offspring of mothers exposed to prenatal stress. *Neural Regeneration Research.* 2011. 6:378-382:

Pierce, A.,Wang, R.,Ryals, J.,Christianson, J.. Voluntary wheel running reverses bladder hypersensitivity and dysfunction resulting from neonatal maternal separation in female mice. *Journal of Pain.* 2015. 16:S65-S65:

Saez, M. C.,Barriga, C.,Rodriguez, A. B.,Garcia, J. J.,Malpica, I.,Ortega, E.. Study of how physical activity and/or melatonin treatment affect the survival of female rats with induced mammary tumours. *Journal of Physiology-London.* 2002. 543:11P-11P:

Schafer, M. J.,Mazula, D. L.,Brown, A. K.,White, T. A.,Atkinson, E.,Pearsall, V. M.,Aversa, Z.,Verzosa, G. C.,Smith, L. A.,Matveyenko, A.,Miller, J. D.,LeBrasseur, N. K.. Late-life time-restricted feeding and exercise differentially alter healthspan in obesity. *Aging Cell.* 2019. 18:e12966:

Straub, O. C.. Experiments to prove a possible influence on the growth of a murine tumour. *Tierärztliche Umschau.* 2005. 60:690-692:German

Sun, Hui Min. The influence of environmental enrichment on cognitive impairment in chronic cerebral hypoperfused rats. *#journal#.* 2010. 10371865:#pages#:Chinese

Sun, R.,Wen, H.,Zeng, Y.. Effects of running wheel exercise and skilled reaching training on the cognitive function in rats with transient middle cerebral artery occlusion at the recovery stage. *Chinese Journal of Rehabilitation Medicine.* 2018. 33:392-396 and 418:

Wang, Y.,Bontempi, B.,Leinekugel, X.,Weinstein, P.,Liu, J.. Environmental enrichment promotes the resolution of post stroke mild cognitive impairment in rats. *Journal of Cerebral Blood Flow and Metabolism.* 2009. 29:S177-S178:

Zhang, C.,Wen, H.,Hu, X.,Li, C.,Zeng, J.. Effects of physical training on pyramidal tract regeneration in hypertensive rats with focal cerebral infarction. *National Medical Journal of China.* 2014. 94:1488-1493:

Zhang, L.,Hu, X.,Zheng, H.. Physical exercise mitigates autophagy and apoptosis after MCAO in rats. *Chinese Journal of Rehabilitation Medicine.* 2017. 32:863-868:

Zhang, L.,Zhang, J.,Sun, H.,Liu, H.,Yang, Y.,Yao, Z.. Exposure to enriched environment restores the

mRNA expression of mineralocorticoid and glucocorticoid receptors in the hippocampus and ameliorates depressive-like symptoms in chronically stressed rats. *Current Neurovascular Research.* 2011. 8:286-93:

Zhang, Q.,Zhang, Q.,Wang, L.. Effects of enriched environment intervention of different intensities on limb functional recovery and neuron growth associated protein expression following cerebral ischemia in rats. *Chinese Journal of Rehabilitation Medicine.* 2012. 27:813-817:

Zheng, H. Q.,Hu, X. Q.,Fang, J.,Pan, S. Q.,Li, L. L.,Zhang, L. Y.. Effects of exercise training on synaptic plasticity in rats with focal cerebral infarction. *National Medical Journal of China.* 2012. 92:628-633:

The article is not available in English or not over 500 words.

Gao, Q.,Wu, Z. Y.,Yao, Z. B.,Yuan, Q. F.. Effects of physical exercise on the acetylcholinesterase-positive fiber density after middle cerebral artery occlusion in mice. *Chinese Journal of Clinical Rehabilitation.* 2003. 7:2274-2275:

Travassos, P. B.,Esteves, J. V. del C.,Moraes, S. M. F. de,Gomes, C. R. de G.. Effects of supplementation by soluble fibers, cafeteria diet and physical exercise on rats' thoracic aorta. *Saúde e Pesquisa.* 2016. 9:111-118:Portuguese

The article is not a primary in vivo research trial.

Hursting, S. D.,Lavigne, J. A.,Berrigan, D.,Donehower, L. A.,Davis, B. J.,Phang, J. M.,Barrett, J. C.,Perkins, S. N.. Diet-gene interactions in p53-deficient mice: insulin-like growth factor-1 as a mechanistic target. *Journal of Nutrition.* 2004. 134:2482S-2486S:English

Zhang, H.,Lee, J. Y.,Borlongan, C. V.,Tajiri, N.. A brief physical activity protects against ischemic stroke. *Brain Circulation.* 2019. 5:112-118:

The article doesn’t use appropriate environmental enrichment.

Alessio, H. M.,Schweitzer, N. B.,Snedden, A. M.,Callahan, P.,Hagerman, A. E.. Revisiting influences on tumor development focusing on laboratory housing. *Journal of the American Association for Laboratory Animal Science.* 2009. 48:258-262:English

Andrianopoulos, G.,Nelson, R. L.,Bombeck, C. T.,Souza, G.. The influence of physical activity in 1,2 dimethylhydrazine induced colon carcinogenesis in the rat. *Anticancer Research.* 1987. 7:849-852:

Aoi, W.,Naito, Y.,Takagi, T.,Kokura, S.,Mizushima, K.,Takanami, Y.,Kawai, Y.,Tanimura, Y.,Hung, L. P.,Koyama, R.,Ichikawa, H.,Yoshikawa, T.. Regular exercise reduces colon tumorigenesis associated with suppression of iNOS. *Biochemical and Biophysical Research Communications.* 2010. 399:14-19:

Aoi, W.,Naito, Y.,Takagi, T.,Tanimura, Y.,Takanami, Y.,Kawai, Y.,Sakuma, K.,Hang, L. P.,Mizushima, K.,Hirai, Y.,Koyama, R.,Wada, S.,Higashi, A.,Kokura, S.,Ichikawa, H.,Yoshikawa, T.. A novel myokine, secreted protein acidic and rich in cysteine (SPARC), suppresses colon tumorigenesis via regular exercise. *Gut.* 2013. 62:882-889:

Arrick, D. M.,Sun, H.,Mayhan, W. G.. Influence of exercise training on ischemic brain injury in type 1 diabetic rats. *Journal of Applied Physiology.* 2012. 113:1121-1127:

Assi, M.,Derbré, F.,Lefeuvre-Orfila, L.,Saligaut, D.,Stock, N.,Ropars, M.,Rébillard, A.. Maintaining a regular physical activity aggravates intramuscular tumor growth in an orthotopic liposarcoma model. *American Journal of Cancer Research.* 2017. 7:1037-1053:

Auriat, A. M.,Colbourne, F.. Influence of amphetamine on recovery after intracerebral hemorrhage in rats. *Behavioural Brain Research.* 2008. 186:222-229:

Auriat, A. M.,Colbourne, F.. Delayed rehabilitation lessens brain injury and improves recovery after intracerebral hemorrhage in rats. *Brain Research.* 2009. 1251:262-268:

Auriat, A. M.,Wowk, S.,Colbourne, F.. Rehabilitation after intracerebral hemorrhage in rats improves recovery with enhanced dendritic complexity but no effect on cell proliferation. *Behavioural Brain Research.* 2010. 214:42-47:

Aveseh, M.,Nikooie, R.,Aminaie, M.. Exercise-induced changes in tumour LDH-B and MCT1 expression are modulated by oestrogen-related receptor alpha in breast cancer-bearing BALB/c mice. *Journal of Physiology-London.* 2015. 593:2635-2648:

Bhagya, V. R.,Srikumar, B. N.,Veena, J.,Shankaranarayana Rao, B. S.. Short-term exposure to enriched environment rescues chronic stress-induced impaired hippocampal synaptic plasticity, anxiety, and memory deficits. *Journal of Neuroscience Research.* 2017. 95:1602-1610:

Biernaskie, J.,Chernenko, G.,Corbett, D.. Efficacy of rehabilitative experience declines with time after focal ischemic brain injury. *Journal of Neuroscience.* 2004. 24:1245-1254:

Caliaperumal, J.,Colbourne, F.. Rehabilitation improves behavioral recovery and lessens cell death without affecting iron, ferritin, transferrin, or inflammation after intracerebral hemorrhage in rats. *Neurorehabilitation and Neural Repair.* 2014. 28:395-404:

Caliaperumal, Jayalakshmi. Mechanisms of injury and recovery after an intracerebral hemorrhage. *#journal#.* 2014. 10099701:207:English

Chabry, J.,Nicolas, S.,Cazareth, J.,Murris, E.,Guyon, A.,Glaichenhaus, N.,Heurteaux, C.,Petit-Paitel, A.. Enriched environment decreases microglia and brain macrophages inflammatory phenotypes through adiponectin-dependent mechanisms: Relevance to depressive-like behavior. *Brain, Behavior, and Immunity.* 2015. 50:275-287:

Chen, C. C.,Chang, M. W.,Chang, C. P.,Chang, W. Y.,Chang, S. C.,Lin, M. T.,Yang, C. L.. Improved infrared-sensing running wheel systems with an effective exercise activity indicator. *PLoS ONE.* 2015. 10:#pages#:

Chen, C. C.,Yang, C. L.,Chang, C. P.. An innovative running wheel-based mechanism for improved rat

training performance. *Journal of Visualized Experiments.* 2016. 2016:#pages#:

Corbianco, S.,Dini, M.,Bongioanni, P.,Carboncini, M. C.,Cavallini, G.. Exercise training in ad libitum and food-restricted old rats: effects on metabolic and physiological parameters. *Biogerontology.* 2020. 21:69-82:

Cymerblit-Sabba, A.,Lasri, T.,Gruper, M.,Aga-Mizrachi, S.,Zubedat, S.,Avital, A.. Prenatal Enriched Environment improves emotional and attentional reactivity to adulthood stress. *Behavioural Brain Research.* 2013. 241:185-190:

Daniels, W. M. U.,Marais, L.,Stein, D. J.,Russell, V. A.. Exercise normalizes altered expression of proteins in the ventral hippocampus of rats subjected to maternal separation. *Experimental Physiology.* 2012. 97:239-247:

Dufresne, S.,Guéritat, J.,Chiavassa, S.,Noblet, C.,Assi, M.,Rioux-Leclercq, N.,Rannou-Bekono, F.,Lefeuvre-Orfila, L.,Paris, F.,Rébillard, A.. Exercise training improves radiotherapy efficiency in a murine model of prostate cancer. *FASEB Journal.* 2020. 34:4984-4996:

Dugger, K. J.,Chrisman, T.,Jones, B.,Chastain, P.,Watson, K.,Estell, K.,Zinn, K.,Schwiebert, L.. Moderate aerobic exercise alters migration patterns of antigen specific T helper cells within an asthmatic lung. *Brain Behavior and Immunity.* 2013. 34:67-78:

Emmons, R.,Xu, G. Y.,Hernandez-Saavedra, D.,Kriska, A.,Pan, Y. X.,Chen, H.,De Lisio, M.. Effects of obesity and exercise on colon cancer induction and hematopoiesis in mice. *American Journal of Physiology-Endocrinology and Metabolism.* 2019. 316:E210-E220:

Figueira, A. C. C.,Figueira, M. C.,Silva, C.,Padrao, A.,Oliveira, P. A.,Ferreira, R. P.,Duarte, J. A.. Exercise Training-induced Modulation in Microenvironment of Rat Mammary Neoplasms. *International Journal of Sports Medicine.* 2018. 39:885-892:

Greenwood, B. N.,Spence, K. G.,Crevling, D. M.,Clark, P. J.,Craig, W. C.,Fleshner, M.. Exercise-induced stress resistance is independent of exercise controllability and the medial prefrontal cortex. *European Journal of Neuroscience.* 2013. 37:469-478:

Halliwell, Celeste I.. Treatment Interventions Following Prenatal Stress and Neonatal Cortical Injury. *#journal#.* 2011. NS00505:350:English

Hayes, K.,Sprague, S.,Guo, M.,Davis, W.,Friedman, A.,Kumar, A.,Jimenez, D. F.,Ding, Y.. Forced, not voluntary, exercise effectively induces neuroprotection in stroke. *Acta Neuropathologica.* 2008. 115:289-296:

Hein, Sarah J.. Anti-Nogo-A immunotherapy facilitation of environmental enrichment's effects on recovery from stroke in the aged rat. *#journal#.* 2010. 1481039:97:English

Hu, X.,Zheng, H.,Yan, T.,Pan, S.,Fang, J.,Jiang, R.,Ma, S.. Physical exercise induces expression of CD31 and facilitates neural function recovery in rats with focal cerebral infarction. *Neurological Research.* 2010. 32:397-402:

Huang, P.,Dong, Z.,Huang, W.,Zhou, C.,Zhong, W.,Hu, P.,Wen, G.,Sun, X.,Hua, H.,Cao, H.,Gao, L.,Lv, Z.. Voluntary wheel running ameliorates depression-like behaviors and brain blood oxygen level-dependent signals in chronic unpredictable mild stress mice. *Behavioural Brain Research.* 2017. 330:17-

24:

Inacio, A. R.,Ruscher, K.,Wieloch, T.. Enriched environment downregulates macrophage migration inhibitory factor and increases parvalbumin in the brain following experimental stroke. *Neurobiology of Disease.* 2011. 41:270-8:

Islas-Preciado, D.,López-Rubalcava, C.,González-Olvera, J.,Gallardo-Tenorio, A.,Estrada-Camarena, E.. Environmental enrichment prevents anxiety-like behavior induced by progesterone withdrawal in two strains of rats. *Neuroscience.* 2016. 336:123-132:

Khamoui, A. V.,Park, B. S.,Kim, D. H.,Yeh, M. C.,Oh, S. L.,Elam, M. L.,Jo, E.,Arjmandi, B. H.,Salazar, G.,Grant, S. C.,Contreras, R. J.,Lee, W. J.,Kim, J. S.. Aerobic and resistance training dependent skeletal muscle plasticity in the colon-26 murine model of cancer cachexia. *Metabolism: Clinical and Experimental.* 2016. 65:685-698:

Kimura, T.,Kubota, M.,Watanabe, H.. Significant improvement in survival of Tabby jimpy mutant mice by providing folded-paper nest boxes. *Scandinavian Journal of Laboratory Animal Science.* 2009. 36:243-249:English

Kloke, V.,Heiming, R. S.,Bolting, S.,Kaiser, S.,Lewejohann, L.,Lesch, K. P.,Sachser, N.. Unexpected effects of early-life adversity and social enrichment on the anxiety profile of mice varying in serotonin transporter genotype. *Behavioural Brain Research.* 2013. 247:248-58:

Leasure, J. L.,Grider, M.. The effect of mild post-stroke exercise on reactive neurogenesis and recovery of somatosensation in aged rats. *Experimental Neurology.* 2010. 226:58-67:

Li, C.,Zhang, B.,Tian, S.,Hu, J.,Gao, B.,Liu, P.,Hua, Y.,Bao, W.,Guan, Y.,Bai, Y.. Early wheel-running promotes functional recovery by improving mitochondria metabolism in olfactory ensheathing cells after ischemic stroke in rats. *Behavioural Brain Research.* 2019. 361:32-38:

Li, M.,Dong, F.,Zhang, F.. Pre-ischemic exercise mitigates brain injury via MEK1/2 and PI3K after ischemic stroke in rats. *International Journal of Clinical and Experimental Medicine.* 2016. 9:10558-10564:

Li, Y.,Zhu, X.,Ju, S.,Yan, J.,Wang, D.,Zhu, Y.,Zang, F.. Detection of volume alterations in hippocampal subfields of rats under chronic unpredictable mild stress using 7T MRI: A follow-up study. *Journal of Magnetic Resonance Imaging.* 2017. 46:1456-1463:

Linecker, M.,Frick, L.,Kron, P.,Limani, P.,Kambakamba, P.,Tschuor, C.,Langiewicz, M.,Kachaylo, E.,Tian, Y.,Schneider, M. A.,Ungethüm, U.,Calo, N.,Foti, M.,Dufour, J. F.,Graf, R.,Humar, B.,Clavien, P. A.. Exercise improves outcomes of surgery on fatty liver in mice: A novel effect mediated by the ampk pathway. *Annals of Surgery.* 2020. 271:347-355:

Liu, C.,Gu, J. Y.,Han, J. H.,Yan, F. L.,Li, Y.,Lv, T. T.,Zhao, L. Q.,Shao, Q. J.,Feng, Y. Y.,Zhang, X. Y.,Wang, C. H.. Enriched environment combined with fluoxetine ameliorates depression-like behaviors and hippocampal SYP expression in a rat CUS model. *Brain Research Bulletin.* 2017. 135:33-39:

Liu, P. F.,Wang, H. Z.,Zhang, J.,Yu, H.,Li, Y. G.,Duan, Y. R.. Effects of Exercise on Antitumor Efficacy of DHAQ-loaded PLA-PLL-RGD Nanoparticles in Hepatocellular Carcinoma. *Materials Research, Pts 1 and 2.* 2009. 610-613:1244-1247:

Liu, W.,Zhou, C.. Corticosterone reduces brain mitochondrial function and expression of mitofusin, BDNF in depression-like rodents regardless of exercise preconditioning. *Psychoneuroendocrinology.* 2012. 37:1057-1070:

Luo, J.,Tang, C.,Chen, X.,Ren, Z.,Qu, H.,Chen, R.,Tong, Z.. Impacts of aerobic exercise on depression-like behaviors in chronic unpredictable mild stress mice and related factors in the AMPK/PGC-1α pathway. *International Journal of Environmental Research and Public Health.* 2020. 17:#pages#:

MacLellan, C. L.,Keough, M. B.,Granter-Button, S.,Chernenko, G. A.,Butt, S.,Corbett, D.. A critical threshold of rehabilitation involving brain-derived neurotrophic factor is required for poststroke recovery. *Neurorehabilitation and Neural Repair.* 2011. 25:740-748:

MacLellan, C. L.,Plummer, N.,Silasi, G.,Auriat, A. M.,Colbourne, F.. Rehabilitation promotes recovery after whole blood-induced intracerebral hemorrhage in rats. *Neurorehabilitation and Neural Repair.* 2011. 25:477-483:

Mahati, K.,Bhagya, V.,Christofer, T.,Sneha, A.,Shankaranarayana Rao, B. S.. Enriched environment ameliorates depression-induced cognitive deficits and restores abnormal hippocampal synaptic plasticity. *Neurobiology of Learning and Memory.* 2016. 134:379-391:

Malicka, I.,Siewierska, K.,Pula, B.,Kobierzycki, C.,Haus, D.,Paslawska, U.,Cegielski, M.,Dziegiel, P.,Podhorska-Okolow, M.,Wozniewski, M.. The effect of physical training on the N-methyl-N-nitrosourea-induced mammary carcinogenesis of Sprague–Dawley rats. *Experimental Biology and Medicine.* 2015. 240:1408-1415:

Mann, P. B.,Jiang, W.,Zhu, Z.,Wolfe, P.,McTiernan, A.,Thompson, H. J.. Wheel running, skeletal muscle aerobic capacity and 1-methyl-1-nitrosourea induced mammary carcinogenesis in the rat. *Carcinogenesis.* 2010. 31:1279-1283:

Marais, L.,Stein, D. J.,Daniels, W. M. U.. Exercise increases BDNF levels in the striatum and decreases depressive-like behavior in chronically stressed rats. *Metabolic Brain Disease.* 2009. 24:587-597:

Marin, R.,Williams, A.,Hale, S.,Burge, B.,Mense, M.,Bauman, R.,Tortella, F.. The effect of voluntary exercise exposure on histological and neurobehavioral outcomes after ischemic brain injury in the rat. *Physiology and Behavior.* 2003. 80:167-175:

Matsumoto, Y.,Adams, V.,Jacob, S.,Mangner, N.,Schuler, G.,Linke, A.. Regular Exercise Training Prevents Aortic Valve Disease in Low-Density Lipoprotein-Receptor-Deficient Mice. *Circulation.* 2010. 121:759-U59:

Mehta, Neha Shailesh. Effects of Sex Differences and Environment on Behavioral and Hippocampal Transcriptomic Endophenotypes in a Genetic Rat Model of Depression. *#journal#.* 2015. 3741334:201:English

Mehta-Raghavan, N. S.,Wert, S. L.,Morley, C.,Graf, E. N.,Redei, E. E.. Nature and nurture: Environmental influences on a genetic rat model of depression. *Translational Psychiatry.* 2016. 6:#pages#:

Mizutani, K.,Sonoda, S.,Karasawa, N.,Yamada, K.,Shimpo, K.,Chihara, T.,Takeuchi, T.,Hasegawa, Y.,Kubo, K. Y.. Effects of exercise after focal cerebral cortex infarction on basal ganglion. *Neurological*

Sciences. 2013. 34:861-867:

Mizutani, K.,Sonoda, S.,Wakita, H.,Katoh, Y.,Shimpo, K.. Functional recovery and alterations in the expression and localization of protein kinase C following voluntary exercise in rat with cerebral infarction. *Neurological Sciences.* 2014. 35:53-59:

Mizutani, K.,Sonoda, S.,Yamada, K.,Beppu, H.,Shimpo, K.. Alteration of protein expression profile following voluntary exercise in the perilesional cortex of rats with focal cerebral infarction. *Brain Research.* 2011. 1416:61-68:

Moreira-Gonçalves, D.,Ferreira, R.,Fonseca, H.,Padrão, A. I.,Moreno, N.,Silva, A. F.,Vasques-Nóvoa, F.,Gonçalves, N.,Vieira, S.,Santos, M.,Amado, F.,Duarte, J. A.,Leite-Moreira, A. F.,Henriques-Coelho, T.. Cardioprotective effects of early and late aerobic exercise training in experimental pulmonary arterial hypertension. *Basic Research in Cardiology.* 2015. 110:#pages#:

Morita, H.,Shindo, M.,Ikeda, S. I.,Yanagisawa, N.. Environmental stimulation increases survival in mice transgenic for exon 1 of the Huntington's disease gene. *Movement Disorders.* 2000. 15:925-937:

Mul, J. D.,Zheng, J.,Goodyear, L. J.. Validity assessment of 5 day repeated forced-swim stress to model human depression in young-adult C57BL/6J and BALB/CJ mice. *eNeuro.* 2016. 3:#pages#:

Nygren, J.,Kokaia, M.,Wieloch, T.. Decreased expression of brain-derived neurotrophic factor in BDNF(+/-) mice is associated with enhanced recovery of motor performance and increased neuroblast number following experimental stroke. *Journal of Neuroscience Research.* 2006. 84:626-31:

Nygren, J.,Wieloch, T.,Pesic, J.,Brundin, P.,Deierborg, T.. Enriched environment attenuates cell genesis in subventricular zone after focal ischemia in mice and decreases migration of newborn cells to the striatum. *Stroke.* 2006. 37:2824-9:

Pan, R. H.,Cai, J.,Zhan, L. C.,Guo, Y. H.,Huang, R. Y.,Li, X.,Zhou, M. C.,Xu, D. D.,Zhan, J.,Chen, H. X.. Buyang Huanwu decoction facilitates neurorehabilitation through an improvement of synaptic plasticity in cerebral ischemic rats. *Bmc Complementary and Alternative Medicine.* 2017. 17:#pages#:

Paré, W. P.,Vincent, G. P.,Isom, K. E.,Reeves, J. M.. Restricted feeding and incidence of activity-stress ulcers in the rat. *Bulletin of the Psychonomic Society.* 1978. 12:143-146:

Pasalic, I.,Bosnjak, B.,Tkalcevic, V. I.,Jaran, D. S.,Javorscak, Z.,Markovic, D.,Hrvacic, B.. Cage enrichment with paper tissue, but not plastic tunnels, increases variability in mouse model of asthma. *Laboratory Animals.* 2011. 45:121-123:English

Plane, J. M.,Whitney, J. T.,Schallert, T.,Parent, J. M.. Retinoic acid and environmental enrichment alter subventricular zone and striatal neurogenesis after stroke. *Experimental Neurology.* 2008. 214:125-134:

Plane, Jennifer Michele. The neurogenic niche and injury-induced neurogenesis. *#journal#.* 2008. 3328931:262:English

Ploughman, M.,Granter-Button, S.,Chernenko, G.,Tucker, B. A.,Mearow, K. M.,Corbett, D.. Endurance exercise regimens induce differential effects on brain-derived neurotrophic factor, synapsin-I and insulin-like growth factor I after focal ischemia. *Neuroscience.* 2005. 136:991-1001:

Re Cecconi, A. D.,Forti, M.,Chiappa, M.,Zhu, Z.,Zingman, L. V.,Cervo, L.,Beltrame, L.,Marchini, S.,Piccirillo, R.. Musclin, a myokine induced by aerobic exercise, retards muscle atrophy during cancer cachexia in mice. *Cancers.* 2019. 11:#pages#:

Rickhag, M.,Deierborg, T.,Patel, S.,Ruscher, K.,Wieloch, T.. Apolipoprotein D is elevated in oligodendrocytes in the peri-infarct region after experimental stroke: influence of enriched environment. *Journal of Cerebral Blood Flow & Metabolism.* 2008. 28:551-62:

Risedal, A.,Zeng, J.,Johansson, B. B.. Early training may exacerbate brain damage after focal brain ischemia in the rat. *Journal of Cerebral Blood Flow and Metabolism.* 1999. 19:997-1003:

Robinson, S.,Christ, C. C.,Cahill, M. M.,Aldrich, S. J.,Taylor-Yeremeeva, E.. Voluntary exercise or systemic propranolol ameliorates stress-related maladaptive behaviors in female rats. *Physiology and Behavior.* 2019. 198:120-133:

Rodriguez Echandia, E. L.,Gonzalez, A. S.,Cabrera, R.,Fracchia, L. N.. A further analysis of behavioral and endocrine effects of unpredictable chronic stress. *Physiology & Behavior.* 1988. 43:789-95:

Russo-Neustadt, A.,Ha, T.,Ramirez, R.,Kesslak, J. P.. Physical activity-antidepressant treatment combination: Impact on brain-derived neurotrophic factor and behavior in an animal model. *Behavioural Brain Research.* 2001. 120:87-95:

Samorajski, T.,Delaney, C.,Durham, L.,Ordy, J. M.,Johnson, J. A.,Dunlap, W. P.. Effect of exercise on longevity, body weight, locomotor performance, and passive-avoidance memory of C57BL/6J mice. *Neurobiology of Aging.* 1985. 6:17-24:

Samorajski, T.,Rolsten, C.,Przykorska, A.,Davis, C. M.. Voluntary wheel running exercise and monoamine levels in brain, heart and adrenal glands of aging mice. *Experimental Gerontology.* 1987. 22:421-431:

Scarola, S. J.,Perdomo Trejo, J. R.,Granger, M. E.,Gerecke, K. M.,Bardi, M.. Immunomodulatory Effects of Stress and Environmental Enrichment in Long-Evans Rats (Rattus norvegicus). *Comparative medicine.* 2019. 69:35-47:

Schuch, C. P.,Balbinot, G.,Jeffers, M. S.,McDonald, M. W.,Dykes, A.,Kuhl, L. M.,Corbett, D.. An RFID-based activity tracking system to monitor individual rodent behavior in environmental enrichment: Implications for post-stroke cognitive recovery. *Journal of Neuroscience Methods.* 2019. 324:#pages#:

Shamsi, M. M.,Chekachak, S.,Soudi, S.,Gharakhanlou, R.,Quinn, L. S.,Ranjbar, K.,Rezaei, S.,Shirazi, F. J.,Allahmoradi, B.,Yazdi, M. H.,Mahdavi, M.,Voltarelli, F. A.. Effects of exercise training and supplementation with selenium nanoparticle on T-helper 1 and 2 and cytokine levels in tumor tissue of mice bearing the 4 T1 mammary carcinoma. *Nutrition.* 2019. 57:141-147:English

Shamsi, M. M.,Chekachak, S.,Soudi, S.,Quinn, L. S.,Ranjbar, K.,Chenari, J.,Yazdi, M. H.,Mahdavi, M.. Combined effect of aerobic interval training and selenium nanoparticles on expression of IL-15 and IL-10/TNF-a ratio in skeletal muscle of 4T1 breast cancer mice with cachexia. *Cytokine.* 2017. 90:100-108:

Shen, J.,Li, Y.,Qu, C.,Xu, L.,Sun, H.,Zhang, J.. The enriched environment ameliorates chronic unpredictable mild stress-induced depressive-like behaviors and cognitive impairment by activating the

SIRT1/miR-134 signaling pathway in hippocampus. *Journal of Affective Disorders.* 2019. 248:81-90:

Sheu, J. R.,Hsieh, C. Y.,Jayakumar, T.,Lin, G. Y.,Lee, H. N.,Huang, S. W.,Yang, C. H.. HDAC6 dysfunction contributes to impaired maturation of adult neurogenesis in vivo: Vital role on functional recovery after ischemic stroke. *Journal of Biomedical Science.* 2019. 26:#pages#:

Shilpa, B. M.,Bhagya, V.,Harish, G.,Srinivas Bharath, M. M.,Shankaranarayana Rao, B. S.. Environmental enrichment ameliorates chronic immobilisation stress-induced spatial learning deficits and restores the expression of BDNF, VEGF, GFAP and glucocorticoid receptors. *Progress in Neuro-Psychopharmacology and Biological Psychiatry.* 2017. 76:88-100:

Siewierska, K.,Malicka, I.,Kobierzycki, C.,Paslawska, U.,Cegielski, M.,Grzegrzolka, J.,Piotrowska, A.,Podhorska-Okolow, M.,Dziegiel, P.,Wozniewski, M.. The Impact of Exercise Training on Breast Cancer. *In Vivo.* 2018. 32:249-254:

Sossdorf, M.,Fischer, J.,Meyer, S.,Dahlke, K.,Wissuwa, B.,Seidel, C.,Schrepper, A.,Bockmeyer, C. L.,Lupp, A.,Neugebauer, S.,Schmerler, D.,Rödel, J.,Claus, R. A.,Otto, G. P.. Physical exercise induces specific adaptations resulting in reduced organ injury and mortality during severe polymicrobial sepsis. *Critical Care Medicine.* 2013. 41:e246-e255:

Sotnikov, S. V.,Markt, P. O.,Malik, V.,Chekmareva, N. Y.,Naik, R. R.,Sah, A.,Singewald, N.,Holsboer, F.,Czibere, L.,Landgraf, R.. Bidirectional rescue of extreme genetic predispositions to anxiety: impact of CRH receptor 1 as epigenetic plasticity gene in the amygdala. *Transl Psychiatry Psychiatry.* 2014. 4:e359:

Spigelman, M. N.,McLeod, W. S.,Rockman, G. E.. Caloric vs. pharmacologic effects of ethanol consumption on activity anorexia in rats. *Pharmacology, Biochemistry and Behavior.* 1991. 39:85-90:

Takai, D.,Abe, A.,Miura, H.,Tanaka, S.,Komura, J.. Minimum environmental enrichment is effective in activating antitumor immunity to transplanted tumor cells in mice. *Experimental Animals.* 2019. 68:569-576:English

Thamizhoviya, G.,Vanisree, A. J.. Enriched environment modulates behavior, myelination and augments molecules governing the plasticity in the forebrain region of rats exposed to chronic immobilization stress. *Metabolic Brain Disease.* 2019. 34:875-887:

Tyler, Christina Rene. Aberrant Neurogenic and Epigenetic Processes Are Associated with Depression Induced by Developmental Arsenic Exposure. *#journal#.* 2014. 3682104:296:English

Veena, J.,Srikumar, B. N.,Mahati, K.,Bhagya, V.,Raju, T. R.,Shankaranarayana Rao, B. S.. Enriched environment restores hippocampal cell proliferation and ameliorates cognitive deficits in chronically stressed rats. *Journal of Neuroscience Research.* 2009. 87:831-843:

Veena, J.,Srikumar, B. N.,Raju, T. R.,Shankaranarayana Rao, B. S.. Exposure to enriched environment restores the survival and differentiation of new born cells in the hippocampus and ameliorates depressive symptoms in chronically stressed rats. *Neuroscience Letters.* 2009. 455:178-182:

Veldink, J. H.,Bär, P. R.,Joosten, E. A. J.,Otten, M.,Wokke, J. H. J.,Van Den Berg, L. H.. Sexual differences in onset of disease and response to exercise in a transgenic model of ALS. *Neuromuscular Disorders.* 2003. 13:737-743:

Wang, D.,Wang, Y.,Ma, J.,Wang, W.,Sun, B.,Zheng, T.,Wei, M.,Sun, Y.. MicroRNA-20a participates in the aerobic exercise-based prevention of coronary artery disease by targeting PTEN. *Biomedicine and Pharmacotherapy.* 2017. 95:756-763:

Warren, K. J.,Olson, M. M.,Thompson, N. J.,Cahill, M. L.,Wyatt, T. A.,Yoon, K. J.,Loiacono, C. M.,Kohut, M. L.. Exercise Improves Host Response to Influenza Viral Infection in Obese and Non-Obese Mice through Different Mechanisms. *Plos One.* 2015. 10:#pages#:

Watson, Lori A.. Social separation in the rat: The investigation of a proposed model of depression. *#journal#.* 2012. 3515374:74:English

Wennerberg, E.,Lhuillier, C.,Rybstein, M. D.,Dannenberg, K.,Rudqvist, N. P.,Koelwyn, G. J.,Jones, L. W.,Demaria, S.. Exercise reduces immune suppression and breast cancer progression in a preclinical model. *Oncotarget.* 2020. 11:452-461:

Wintink, Amanda J.. The role of the hippocampus in amygdala-kindled fear in male and female rats. *#journal#.* 2005. NR00961:137:English

Wood, N. I.,Carta, V.,Milde, S.,Skillings, E. A.,McAllister, C. J.,Mabel Ang, Y. L.,Duguid, A.,Wijesuriya, N.,Afzal, S. M.,Fernandes, J. X.,Leong, T. W.,Morton, J.. Responses to environmental enrichment differ with sex and genotype in a transgenic mouse model of huntington's disease. *PLoS ONE.* 2010. 5:#pages#:

Wood, N. I.,Glynn, D.,Morton, A. J.. "Brain training" improves cognitive performance and survival in a transgenic mouse model of Huntington's disease. *Neurobiology of Disease.* 2011. 42:427-437:

Xiao, Q.,Wang, F. F.,Luo, Y. M.,Chen, L. M.,Chao, F. L.,Tan, C. X.,Gao, Y.,Huang, C. X.,Zhang, L.,Liang, X.,Tang, J.,Qi, Y. Q.,Jiang, L.,Zhang, Y.,Zhou, C. N.,Tang, Y.. Exercise protects myelinated fibers of white matter in a rat model of depression. *Journal of Comparative Neurology.* 2018. 526:537-549:

Xie, H. Y.,Wu, Y.,Jia, J.,Liu, G.,Zhang, F.,Zhang, Q.,Yu, K. W.,Hu, Y. S.,Bai, Y. L.,Hu, R. P.. Enriched environment preconditioning induced brain ischemic tolerance without reducing infarct volume and edema: The possible role of enrichment-related physical activity increase. *Brain Research.* 2013. 1508:63-72:

Xie, H.,Yu, K.,Zhou, N.,Shen, X.,Tian, S.,Zhang, B.,Wang, Y.,Wu, J.,Liu, G.,Jiang, C.,Hu, R.,Ayata, C.,Wu, Y.. Enriched Environment Elicits Proangiogenic Mechanisms After Focal Cerebral Ischemia. *Translational Stroke Research.* 2019. 10:150-159:

Xie, H.,Zhang, Q.,Zhou, N.,Li, C.,Yu, K.,Liu, G.,Wu, J.,Jiang, C.,Hu, R.,Wu, Y.. Environmental enrichment enhances post-ischemic cerebral blood flow and functional hyperemia in the ipsilesional somatosensory cortex. *Brain Research Bulletin.* 2020. 160:91-97:

Yang, L. Q.,Zhang, J.,Deng, Y. H.,Zhang, P. Y.. The Effects of Early Exercise on Motor, Sense, and Memory Recovery in Rats With Stroke. *American Journal of Physical Medicine & Rehabilitation.* 2017. 96:E36-E43:

Zarobkiewicz, M. K.,Sławiński, M. A.,Wawryk-Gawda, E.,Woźniakowski, M. M.,Kulak-Janczy, E.,Korzeniowska, S.,Jodłowska-Jędrych, B.. Changes in histological structure and nitric oxide synthase expression in aorta of rats supplemented with bee pollen or whey protein. *Applied Physiology, Nutrition*

and Metabolism. 2019. 44:1150-1158:English

Zhan, Y.,Li, M. Z.,Yang, L.,Feng, X. F.,Zhang, Q. X.,Zhang, N.,Zhao, Y. Y.,Zhao, H.. An MRI study of neurovascular restorative after combination treatment with xiaoshuanenteric-coated capsule and enriched environment in rats after stroke. *Frontiers in Neuroscience.* 2019. 13:#pages#:

Zhou, Z. W.,Yang, Q. D.,Tang, Q. P.,Yang, J.,Guo, R. J.,Jiang, W.. Effect of willed movement training on neurorehabilitation after focal cerebral ischemia and on the neural plasticity-associated signaling pathway. *Molecular Medicine Reports.* 2018. 17:1173-1181:

Zhu, Z. J.,Jiang, W. Q.,Zacher, J. H.,Neil, E. S.,McGinley, J. N.,Thompson, H. J.. Effects of energy restriction and wheel running on mammary carcinogenesis and host systemic factors in a rat model. *Cancer Prevention Research.* 2012. 5:414-422:English

Zou, J.,Yuan, J.,Lv, S.,Tu, J.. Effects of exercise on behavior and peripheral blood lymphocyte apoptosis in a rat model of chronic fatigue syndrome. *Journal of Huazhong University of Science and Technology - Medical Science.* 2010. 30:258-264:

The article does not use appropriate conventional housing.

Ashokan, A.,Hegde, A.,Balasingham, A.,Mitra, R.. Housing environment influences stress-related hippocampal substrates and depression-like behavior. *Brain Research.* 2018. 1683:78-85:

Ashokan, A.,Hegde, A.,Mitra, R.. Short-term environmental enrichment is sufficient to counter stress-induced anxiety and associated structural and molecular plasticity in basolateral amygdala. *Psychoneuroendocrinology.* 2016. 69:189-196:

Badowska, D. M.,Brzozka, M. M.,Chowdhury, A.,Malzahn, D.,Rossner, M. J.. Data calibration and reduction allows to visualize behavioural profiles of psychosocial influences in mice towards clinical domains. *European Archives of Psychiatry & Clinical Neuroscience.* 2015. 265:483-96:

Basterfield, L.,Mathers, J. C.. Intestinal tumours, colonic butyrate and sleep in exercised Min mice. *British Journal of Nutrition.* 2010. 104:355-363:English

Belayev, A.,Saul, I.,Liu, Y.,Zhao, W.,Ginsberg, M. D.,Valdes, M. A.,Busto, R.,Belayev, L.. Enriched environment delays the onset of hippocampal damage after global cerebral ischemia in rats. *Brain Research.* 2003. 964:121-127:

Beltran, E. J.,Papadopoulos, C. M.,Tsai, S. Y.,Kartje, G. L.,Wolf, W. A.. Long-term motor improvement after stroke is enhanced by short-term treatment with the alpha-2 antagonist, atipamezole. *Brain Research.* 2010. 1346:174-82:

Biernaskie, J.,Corbett, D.. Enriched rehabilitative training promotes improved forelimb motor function and enhanced dendritic growth after focal ischemic injury. *Journal of Neuroscience.* 2001. 21:5272-5280:

Branchi, I.,Santarelli, S.,Capoccia, S.,Poggini, S.,Cirulli, F.,Alleva, E.. Antidepressant Treatment Outcome Depends on the Quality of the Living Environment: A Pre-Clinical Investigation in Mice. *PLoS ONE.* 2013. 8:#pages#:

Buchhold, B.,Mogoanta, L.,Suofu, Y.,Hamm, A.,Walker, L.,Kessler, Ch,Popa-Wagner, A.. Environmental enrichment improves functional and neuropathological indices following stroke in young and aged rats. *Restorative Neurology and Neuroscience.* 2007. 25:467-484:

Castelhano-Carlos, M.,Costa, P. S.,Russig, H.,Sousa, N.. Pheno World: A new paradigm to screen rodent behavior. *Translational Psychiatry.* 2014. 4:#pages#:

Clarke, J.,Langdon, K. D.,Corbett, D.. Early poststroke experience differentially alters periinfarct layer II and III cortex. *Journal of Cerebral Blood Flow & Metabolism.* 2014. 34:630-7:

Clarke, J.,Mala, H.,Windle, V.,Chernenko, G.,Corbett, D.. The effects of repeated rehabilitation "tune-ups" on functional recovery after focal ischemia in rats. *Neurorehabilitation & Neural Repair.* 2009. 23:886-94:

Clarke, Jared. An exploration of possible mechanisms underlying the beneficial effects of enriched rehabilitation on post -stroke recovery of function. *#journal#.* 2010. NR64754:185:English

Cordner, Z. A.,Tamashiro, K. L. K.. Effects of chronic variable stress on cognition and Bace1 expression among wild-type mice. *Translational Psychiatry.* 2016. 6:#pages#:

Dahlqvist, P.,Rönnbäck, A.,Bergström, S. A.,Söderström, I.,Olsson, T.. Environmental enrichment reverses learning impairment in the Morris water maze after focal cerebral ischemia in rats. *European Journal of Neuroscience.* 2004. 19:2288-2298:

Dandi, E,Kalamari, A.,Touloumi, O.,Lagoudaki, R.,Nousiopoulou, E.,Simeonidou, C.,Spandou, E.,Tata, D. A.. Beneficial effects of environmental enrichment on behavior, stress reactivity and synaptophysin/BDNF expression in hippocampus following early life stress. *International Journal of Developmental Neuroscience.* 2018. 67:19-32:

Di Cataldo, V.,Géloën, A.,Langlois, J. B.,Chauveau, F.,Thézé, B.,Hubert, V.,Wiart, M.,Chirico, E. N.,Rieusset, J.,Vidal, H.,Pialoux, V.,Canet-Soulas, E.. Exercise does not protect against peripheral and central effects of a high cholesterol diet given ad libitum in old ApoE-/- mice. *Frontiers in Physiology.* 2016. 7:#pages#:

do Prado, C. H.,Narahari, T.,Holland, F. H.,Lee, H. N.,Murthy, S. K.,Brenhouse, H. C.. Effects of early adolescent environmental enrichment on cognitive dysfunction, prefrontal cortex development, and inflammatory cytokines after early life stress. *Developmental Psychobiology.* 2016. 58:482-491:

Doreste-Mendez, R.,Ríos-Ruiz, E. J.,Rivera-López, L. L.,Gutierrez, A.,Torres-Reveron, A.. Effects of Environmental Enrichment in Maternally Separated Rats: Age and Sex-Specific Outcomes. *Frontiers in Behavioral Neuroscience.* 2019. 13:#pages#:

Fan, X.,Li, D.,Lichti, C. F.,Green, T. A.. Dynamic Proteomics of Nucleus Accumbens in Response to Acute Psychological Stress in Environmentally Enriched and Isolated Rats. *PLoS ONE.* 2013. 8:#pages#:

Francis, D. D.,Diorio, J.,Plotsky, P. M.,Meaney, M. J.. Environmental enrichment reverses the effects of maternal separation on stress reactivity. *Journal of Neuroscience.* 2002. 22:7840-7843:

Fukao, K.,Shimada, K.,Naito, H.,Sumiyoshi, K.,Inoue, N.,Lesaki, T.,Kume, A.,Kiyanagi, T.,Hiki, M.,Hirose, K.,Matsumori, R.,Ohsaka, H.,Takahashi, Y.,Toyoda, S.,Itoh, S.,Miyazaki, T.,Tada,

N.,Daida, H.. Voluntary exercise ameliorates the progression of atherosclerotic lesion formation via anti-inflammatory effects in apolipoprotein e-deficient mice. *Journal of Atherosclerosis and Thrombosis.* 2010. 17:1226-1236:

Garbugino, L.,Golini, E.,Giuliani, A.,Mandillo, S.. Prolonged voluntary running negatively affects survival and disease prognosis of male SOD1G93A low-copy transgenic mice. *Frontiers in Behavioral Neuroscience.* 2018. 12:#pages#:

Garofalo, S.,D'Alessandro, G.,Chece, G.,Brau, F.,Maggi, L.,Rosa, A.,Porzia, A.,Mainiero, F.,Esposito, V.,Lauro, C.,Benigni, G.,Bernardini, G.,Santoni, A.,Limatola, C.. Enriched environment reduces glioma growth through immune and non-immune mechanisms in mice. *Nature Communications.* 2015. 6:#pages#:

Garofalo, S.,Porzia, A.,Mainiero, F.,Di Angelantonio, S.,Cortese, B.,Basilico, B.,Pagani, F.,Cignitti, G.,Chece, G.,Maggio, R.,Tremblay, M. E.,Savage, J.,Bisht, K.,Esposito, V.,Bernardini, G.,Seyfried, T.,Mieczkowski, J.,Stepniak, K.,Kaminska, B.,Santoni, A.,Limatola, C.. Environmental stimuli shape microglial plasticity in glioma. *eLife.* 2017. 6:#pages#:

Grabowski, M.,Sørensen, J. C.,Mattsson, B.,Zimmer, J.,Johansson, B. B.. Influence of an Enriched Environment and Cortical Grafting on Functional Outcome in Brain Infarcts of Adult Rats. *Experimental Neurology.* 1995. 133:96-102:

Grady, D. L.,Thanos, P. K.,Corrada, M. M.,Barnett Jr, J. C.,Ciobanu, V.,Shustarovich, D.,Napoli, A.,Moyzis, A. G.,Grandy, D.,Rubinstein, M.,Wang, G. J.,Kawas, C. H.,Chen, C.,Dong, Q.,Wang, E.,Volkow, N. D.,Moyzis, R. K.. DRD4 genotype predicts longevity in mouse and human. *Journal of Neuroscience.* 2013. 33:286-291:

Hakon, J.,Quattromani, M. J.,Sjölund, C.,Tomasevic, G.,Carey, L.,Lee, J. M.,Ruscher, K.,Wieloch, T.,Bauer, A. Q.. Multisensory stimulation improves functional recovery and resting-state functional connectivity in the mouse brain after stroke. *NeuroImage: Clinical.* 2018. 17:717-730:

Hicks, A. U.,Hewlett, K.,Windle, V.,Chernenko, G.,Ploughman, M.,Jolkkonen, J.,Weiss, S.,Corbett, D.. Enriched environment enhances transplanted subventricular zone stem cell migration and functional recovery after stroke. *Neuroscience.* 2007. 146:31-40:

Hicks, A. U.,Lappalainen, R. S.,Narkilahti, S.,Suuronen, R.,Corbett, D.,Sivenius, J.,Hovatta, O.,Jolkkonen, J.. Transplantation of human embryonic stem cell-derived neural precursor cells and enriched environment after cortical stroke in rats: Cell survival and functional recovery. *European Journal of Neuroscience.* 2009. 29:562-574:

Hicks, A. U.,MacLellan, C. L.,Chernenko, G. A.,Corbett, D.. Long-term assessment of enriched housing and subventricular zone derived cell transplantation after focal ischemia in rats. *Brain Research.* 2008. 1231:103-112:

Hirata, K.,Kuge, Y.,Yokota, C.,Harada, A.,Kokame, K.,Inoue, H.,Kawashima, H.,Hanzawa, H.,Shono, Y.,Saji, H.,Minematsu, K.,Tamaki, N.. Gene and protein analysis of brain derived neurotrophic factor expression in relation to neurological recovery induced by an enriched environment in a rat stroke model. *Neuroscience Letters.* 2011. 495:210-215:

Hiroux, C.,Vandoorne, T.,Koppo, K.,De Smet, S.,Hespel, P.,Berardi, E.. Physical Activity Counteracts Tumor Cell Growth in Colon Carcinoma C26-Injected Muscles: An Interim Report.

European Journal of Translational Myology. 2016. 26:5958:

Hutchinson, K. M.,McLaughlin, K. J.,Wright, R. L.,Ortiz, J. B.,Anouti, D. P.,Mika, A.,Diamond, D. M.,Conrad, C. D.. Environmental enrichment protects against the effects of chronic stress on cognitive and morphological measures of hippocampal integrity. *Neurobiology of Learning and Memory*. 2012. 97:250-260:

Huzard, D.,Mumby, D. G.,Sandi, C.,Poirier, G. L.,Kooij, M. A. van der. The effects of extrinsic stress on somatic markers and behavior are dependent on animal housing conditions. *Physiology & Behavior*. 2015. 151:238-245:English

Jeffers, M. S.,Corbett, D.. Synergistic effects of enriched environment and task-specific reach training on poststroke recovery of motor function. *Stroke*. 2018. 49:1496-1503:

Jin, J.,Kang, H. M.,Park, C.. Voluntary exercise enhances survival and migration of neural progenitor cells after intracerebral haemorrhage in mice. *Brain Injury*. 2010. 24:533-540:

Jolkkonen, J.,Gallagher, N. P.,Zilles, K.,Sivenius, J.. Behavioral deficits and recovery following transient focal cerebral ischemia in rats: Glutamatergic and GABAergic receptor densities. *Behavioural Brain Research*. 2003. 138:187-200:

Jurgens, H. A.,Johnson, R. W.. Environmental enrichment attenuates hippocampal neuroinflammation and improves cognitive function during influenza infection. *Brain, Behavior, and Immunity*. 2012. 26:1006-1016:

Jurgens, Heidi Ann. Influenza infection induces neuroinflammation and impacts hippocampal structure and function. *#journal#*. 2012. 3570503:150:English

Karhunen, H.,Virtanen, T.,Schallert, T.,Sivenius, J.,Jolkkonen, J.. Forelimb use after focal cerebral ischemia in rats treated with an alpha(2)-adrenoceptor antagonist. *Pharmacology Biochemistry and Behavior*. 2003. 74:663-669:

Kazl, C.,Foote, L. T.,Kim, M. J.,Koh, S.. Early-life experience alters response of developing brain to seizures. *Brain Research*. 2009. 1285:174-181:

Kelly, S. A.,Zhao, L.,Jung, K. C.,Hua, K.,Threadgill, D. W.,Kim, Y.,De Villena, F. P. M.,Pomp, D.. Prevention of tumorigenesis in mice by exercise is dependent on strain background and timing relative to carcinogen exposure. *Scientific Reports*. 2017. 7:#pages#:

Koe, A. S.,Ashokan, A.,Mitra, R.. Short environmental enrichment in adulthood reverses anxiety and basolateral amygdala hypertrophy induced by maternal separation. *Translational Psychiatry*. 2016. 6:#pages#:

Kuptsova, K.,Kvist, E.,Nitzsche, F.,Jolkkonen, J.. Combined enriched environment/atipamezole treatment transiently improves sensory functions in stroke rats independent from neurogenesis and angiogenesis. *Romanian Journal of Morphology and Embryology*. 2015. 56:41-47:

Lehmann, M. L.,Brachman, R. A.,Martinowich, K.,Schloesser, R. J.,Herkenham, M.. Glucocorticoids orchestrate divergent effects on mood through adult neurogenesis. *Journal of Neuroscience*. 2013. 33:2961-2972:

Madinier, A.,Quattromani, M. J.,Sjolund, C.,Ruscher, K.,Wieloch, T.. Enriched housing enhances recovery of limb placement ability and reduces aggrecan-containing perineuronal nets in the rat somatosensory cortex after experimental stroke. *PLoS ONE [Electronic Resource].* 2014. 9:e93121:

Matsumori, Y.,Hong, S. M.,Fan, Y.,Kayama, T.,Hsu, C. Y.,Weinstein, P. R.,Liu, J.. Enriched environment and spatial learning enhance hippocampal neurogenesis and salvages ischemic penumbra after focal cerebral ischemia. *Neurobiology of Disease.* 2006. 22:187-98:

Mayeda, A. R.,Hofstetter, J. R.,Possidente, B.. Aging lengthens TauDD in C57BL/6J, DBA/2J, and outbred SWR male mice (Mus musculus). *Chronobiology International.* 1997. 14:19-23:

McCreary, J. K.,Erickson, Z. T.,Hao, Y.,Ilnytskyy, Y.,Kovalchuk, I.,Metz, G. A. S.. Environmental intervention as a therapy for adverse programming by ancestral stress. *Scientific Reports.* 2016. 6:#pages#:

Ohlsson, A. L.,Johansson, B. B.. Environment influences functional outcome of cerebral infarction in rats. *Stroke.* 1995. 26:644-649:

Papadopoulos, C. M.,Tsai, S. Y.,Guillen, V.,Ortega, J.,Kartje, G. L.,Wolf, W. A.. Motor recovery and axonal plasticity with short-term amphetamine after stroke. *Stroke.* 2009. 40:294-302:

Pritchard, L. M.,Van Kempen, T. A.,Zimmerberg, B.. Behavioral effects of repeated handling differ in rats reared in social isolation and environmental enrichment. *Neuroscience Letters.* 2013. 536:47-51:

Quattromani, M. J.,Cordeau, P.,Ruscher, K.,Kriz, J.,Wieloch, T.. Enriched housing down-regulates the Toll-like receptor 2 response in the mouse brain after experimental stroke. *Neurobiology of Disease.* 2014. 66:66-73:

Quattromani, M. J.,Pruvost, M.,Guerreiro, C.,Backlund, F.,Englund, E.,Aspberg, A.,Jaworski, T.,Hakon, J.,Ruscher, K.,Kaczmarek, L.,Vivien, D.,Wieloch, T.. Extracellular Matrix Modulation Is Driven by Experience-Dependent Plasticity During Stroke Recovery. *Molecular Neurobiology.* 2018. 55:2196-2213:

Rojo, M. L.,Söderström, I.,Fowler, C. J.. Residual effects of focal brain ischaemia upon cannabinoid CB1 receptor density and functionality in female rats. *Brain Research.* 2011. 1373:195-201:

Rönnbäck, A.,Dahlqvist, P.,Svensson, P. A.,Jernås, M.,Carlsson, B.,Carlsson, L. M. S.,Olsson, T.. Gene expression profiling of the rat hippocampus one month after focal cerebral ischemia followed by enriched environment. *Neuroscience Letters.* 2005. 385:173-178:

Ruscher, K.,Johannesson, E.,Brugiere, E.,Erickson, A.,Rickhag, M.,Wieloch, T.. Enriched environment reduces apolipoprotein E (ApoE) in reactive astrocytes and attenuates inflammation of the peri-infarct tissue after experimental stroke. *Journal of Cerebral Blood Flow and Metabolism.* 2009. 29:1796-1805:

Ruscher, K.,Kuric, E.,Liu, Y.,Walter, H. L.,Issazadeh-Navikas, S.,Englund, E.,Wieloch, T.. Inhibition of CXCL12 signaling attenuates the postischemic immune response and improves functional recovery after stroke. *Journal of Cerebral Blood Flow & Metabolism.* 2013. 33:1225-34:

Segovia, G.,Del Arco, A.,de Blas, M.,Garrido, P.,Mora, F.. Effects of an enriched environment on the release of dopamine in the prefrontal cortex produced by stress and on working memory during aging in

the awake rat. *Behavioural Brain Research.* 2008. 187:304-11:

Seong, H. H.,Park, J. M.,Kim, Y. J.. Antidepressive Effects of Environmental Enrichment in Chronic Stress–Induced Depression in Rats. *Biological Research for Nursing.* 2018. 20:40-48:

Shono, Y.,Yokota, C.,Kuge, Y.,Kido, S.,Harada, A.,Kokame, K.,Inoue, H.,Hotta, M.,Hirata, K.,Saji, H.,Tamaki, N.,Minematsu, K.. Gene expression associated with an enriched environment after transient focal ischemia. *Brain Research.* 2011. 1376:60-65:

Smith, B. L.,Morano, R. L.,Ulrich-Lai, Y. M.,Myers, B.,Solomon, M. B.,Herman, J. P.. Adolescent environmental enrichment prevents behavioral and physiological sequelae of adolescent chronic stress in female (but not male) rats. *Stress.* 2018. 21:464-473:

Söderström, I.,Strand, M.,Ingridsson, A. C.,Nasic, S.,Olsson, T.. 17β-estradiol and enriched environment accelerate cognitive recovery after focal brain ischemia. *European Journal of Neuroscience.* 2009. 29:1215-1224:

Sonninen, R.,Virtanen, T.,Sivenius, J.,Jolkkonen, J.. Gene expression profiling in the hippocampus of rats subjected to focal cerebral ischemia and enriched environment housing. *Restorative Neurology and Neuroscience.* 2006. 24:17-23:

Thanos, P. K.,Hamilton, J.,O'Rourke, J. R.,Napoli, A.,Febo, M.,Volkow, N. D.,Blum, K.,Gold, M.. Dopamine D2 gene expression interacts with environmental enrichment to impact lifespan and behavior. *Oncotarget.* 2016. 7:19111-19123:

Vivinetto, A. L.,Suárez, M. M.,Rivarola, M. A.. Neurobiological effects of neonatal maternal separation and post-weaning environmental enrichment. *Behavioural Brain Research.* 2013. 240:110-118:

Wadowska, M.,Woods, J.,Rogozinska, M.,Briones, T. L.. Neuroprotective effects of enriched environment housing after transient global cerebral ischaemia are associated with the upregulation of insulin-like growth factor-1 signalling. *Neuropathology and Applied Neurobiology.* 2015. 41:544-556:

Wahl, A. S.,Erlebach, E.,Brattoli, B.,Büchler, U.,Kaiser, J.,Ineichen, B. V.,Mosberger, A. C.,Schneeberger, S.,Imobersteg, S.,Wieckhorst, M.,Stirn, M.,Schroeter, A.,Ommer, B.,Schwab, M. E.. Early reduced behavioral activity induced by large strokes affects the efficiency of enriched environment in rats. *Journal of Cerebral Blood Flow and Metabolism.* 2019. 39:2022-2034:

Wang, Y.,Bontempi, B.,Hong, S. M.,Mehta, K.,Weinstein, P. R.,Abrams, G. M.,Liu, J.. A comprehensive analysis of gait impairment after experimental stroke and the therapeutic effect of environmental enrichment in rats. *Journal of Cerebral Blood Flow and Metabolism.* 2008. 28:1936-1950:

Woitke, F.,Ceanga, M.,Rudolph, M.,Niv, F.,Witte, O. W.,Redecker, C.,Kunze, A.,Keiner, S.. Adult hippocampal neurogenesis poststroke: More new granule cells but aberrant morphology and impaired spatial memory. *PLoS ONE.* 2017. 12:#pages#:

Wright, Ryan L.. Improving cognitive outcomes following chronic stress. *#journal#.* 2007. 3288034:134:English

Xu, X.,Ye, L.,Ruan, Q.. Environmental enrichment induces synaptic structural modification after transient focal cerebral ischemia in rats. *Experimental Biology and Medicine.* 2009. 234:296-305:

Zai, L.,Ferrari, C.,Dice, C.,Subbaiah, S.,Havton, L. A.,Coppola, G.,Geschwind, D.,Irwin, N.,Huebner, E.,Strittmatter, S. M.,Benowitz, L. I.. Inosine augments the effects of a Nogo receptor blocker and of environmental enrichment to restore skilled forelimb use after stroke. *Journal of Neuroscience.* 2011. 31:5977-5988:

Zanca, R. M.,Braren, S. H.,Maloney, B.,Schrott, L. M.,Luine, V. N.,Serrano, P. A.. Environmental enrichment increases glucocorticoid receptors and decreases GluA2 and protein kinase M Zeta (PKMζ) trafficking during chronic stress: A protective mechanism?. *Frontiers in Behavioral Neuroscience.* 2015. 9:#pages#:

Zhang, J.,He, Z. X.,Wang, L. M.,Yuan, W.,Li, L. F.,Hou, W. J.,Yang, Y.,Guo, Q. Q.,Zhang, X. N.,Cai, W. Q.,An, S. C.,Tai, F. D.. Voluntary wheel running reverses deficits in social behavior induced by chronic social defeat stress in mice: Involvement of the dopamine system. *Frontiers in Neuroscience.* 2019. 13:#pages#:

Zhang, Y.,Crofton, E. J.,Li, D.,Lobo, M. K.,Fan, X.,Nestler, E. J.,Green, T. A.. Overexpression of DeltaFosB in nucleus accumbens mimics the protective addiction phenotype, but not the protective depression phenotype of environmental enrichment. *Frontiers in Behavioral Neuroscience.* 2014. 8:#pages#:

Zhu, L.,Wang, J.,Pettan-Brewer, C.,Ladiges, W.,Goh, J.. Wheel running predicts resilience to tumors in old mice. *Pathobiology of Aging & Age Related Diseases.* 2019. 9:1676104:

The article does not include a disease model of interest.

Assi, M.,Kenawi, M.,Ropars, M.,Rébillard, A.. Interleukin-6, C/EBP-β and PPAR-γ expression correlates with intramuscular liposarcoma growth in mice: The impact of voluntary physical activity levels. *Biochemical and Biophysical Research Communications.* 2017. 490:1026-1032:

Baldini, S.,Restani, L.,Baroncelli, L.,Coltelli, M.,Franco, R.,Cenni, M. C.,Maffei, L.,Berardi, N.. Enriched early life experiences reduce adult anxiety-like behavior in rats: A role for insulin-like growth factor 1. *Journal of Neuroscience.* 2013. 33:11715-11723:

Bell, J. A.,Livesey, P. J.,Meyer, J. F.. Environmental enrichment influences survival rate and enhances exploration and learning but produces variable responses to the radial maze in old rats. *Developmental Psychobiology.* 2009. 51:564-578:

Brenes Sáenz, J. C.,Villagra, O. R.,Fornaguera Trías, J.. Factor analysis of Forced Swimming test, Sucrose Preference test and Open Field test on enriched, social and isolated reared rats. *Behavioural Brain Research.* 2006. 169:57-65:

Buehlmeyer, K.,Doering, F.,Daniel, H.,Petridou, A.,Mougios, V.,Schulz, T.,Michna, H.. IGF-1 gene expression in rat colonic mucosa after different exercise volumes. *Journal of Sports Science and Medicine.* 2007. 6:434-440:

Candemir, E.,Post, A.,Dischinger, U. S.,Palme, R.,Slattery, D. A.,O'Leary, A.,Reif, A.. Limited effects of early life manipulations on sex-specific gene expression and behavior in adulthood. *Behavioural Brain Research.* 2019. 369:#pages#:

Chourbaji, S.,Zacher, C.,Sanchis-Segura, C.,Spanagel, R.,Gass, P.. Social and structural housing conditions influence the development of a depressive-like phenotype in the learned helplessness paradigm in male mice. *Behavioural Brain Research.* 2005. 164:100-6:

Coletti, D.,Aulino, P.,Pigna, E.,Barteri, F.,Moresi, V.,Annibali, D.,Adamo, S.,Berardi, E.. Spontaneous physical activity downregulates pax7 in cancer cachexia. *Stem Cells International.* 2016. 2016:#pages#:

Cook, Marc D.. The effects of treadmill and voluntary wheel training on morbidity and inflammation during ulcerative colitis in mice. *#journal#.* 2013. 3614564:125:English

Counts, B. R.,Hardee, J. P.,Fix, D. K.,Vanderveen, B. N.,Montalvo, R. N.,Carson, J. A.. Cachexia Disrupts Diurnal Regulation of Activity, Feeding, and Muscle mTORC1 in Mice. *Medicine and Science in Sports and Exercise.* 2020. 52:577-587:

Fleenor, B. S.,Marshall, K. D.,Durrant, J. R.,Lesniewski, L. A.,Seals, D. R.. Arterial stiffening with ageing is associated with transforming growth factor-β1-related changes in adventitial collagen: Reversal by aerobic exercise. *Journal of Physiology.* 2010. 588:3971-3982:

Freret, T.,Billard, J. M.,Schumann-Bard, P.,Dutar, P.,Dauphin, F.,Boulouard, M.,Bouet, V.. Rescue of cognitive aging by long-lasting environmental enrichment exposure initiated before median lifespan. *Neurobiology of Aging.* 2012. 33:1005.e1:

Glavin, G. B.. Fat-supplemented diet protects against activity-stress ulcers in rats. *Experientia.* 1983. 39:1097-1099:English

Gordon, C. J.,Phillips, P. M.,Ledbetter, A.,Snow, S. J.,Schladweiler, M. C.,Johnstone, A. F. M.,Kodavanti, U. P.. Active vs. Sedentary lifestyle from weaning to adulthood and susceptibility to ozone in rats. *American Journal of Physiology - Lung Cellular and Molecular Physiology.* 2017. 312:L100-L109:

Green, A.,Esser, M. J.,Perrot, T. S.. Developmental expression of anxiety and depressive behaviours after prenatal predator exposure and early life homecage enhancement. *Behavioural Brain Research.* 2018. 346:122-136:

Greifzu, F.,Pielecka-Fortuna, J.,Kalogeraki, E.,Krempler, K.,Favaro, P. D.,Schlüter, O. M.,Löwel, S.. Environmental enrichment extends ocular dominance plasticity into adulthood and protects from stroke-induced impairments of plasticity. *Proceedings of the National Academy of Sciences of the United States of America.* 2014. 111:1150-1155:

Guan, S. Z.,Ji, W. J.,Jiang, Y.,Ning, L.,Lian, Y. L.,Liu, J. W.. Enriched environment treatment remediated hippocampal monoamine neurotransmitters and emotional deficits in offspring induced by maternal chronic stress rat during pregnancy. *International Journal of Clinical and Experimental Medicine.* 2017. 10:9963-9975:

Hendriksen, H.,Prins, J.,Olivier, B.,Oosting, R. S.. Environmental enrichment induces behavioral recovery and enhanced hippocampal cell proliferation in an antidepressant-resistant animal model for PTSD. *PLoS ONE.* 2010. 5:#pages#:

Jonsdottir, I. H.,Johansson, C.,Asea, A.,Johansson, P.,Hellstrand, K.,Thoren, P.,Hoffmann, P..

Duration and mechanisms of the increased natural cytotoxicity seen after chronic voluntary exercise in rats. *Acta Physiologica Scandinavica.* 1997. 160:333-339:

Klenotich, S. J.,Seiglie, M. P.,McMurray, M. S.,Roitman, J. D.,Grange, D. le,Dugad, P.,Dulawa, S. C.. Olanzapine, but not fluoxetine, treatment increases survival in activity-based anorexia in mice. *Neuropsychopharmacology.* 2012. 37:1620-1631:English

Konhilas, J. P.,Watson, P. A.,Maass, A.,Boucek, D. M.,Horn, T.,Stauffer, B. L.,Luckey, S. W.,Rosenberg, P.,Leinwand, L. A.. Exercise can prevent and reverse the severity of hypertrophic cardiomyopathy. *Circulation Research.* 2006. 98:540-548:

Laufs, U.,Werner, N.,Link, A.,Endres, M.,Wassmann, S.,Jürgens, K.,Miche, E.,Böhm, M.,Nickenig, G.. Physical Training Increases Endothelial Progenitor Cells, Inhibits Neointima Formation, and Enhances Angiogenesis. *Circulation.* 2004. 109:220-226:

Leblond, F.,Nguyen, A.,Bolduc, V.,Lambert, J.,Yu, C.,Duquette, N.,Thorin, E.. Postnatal exposure to voluntary exercise but not the antioxidant catechin protects the vasculature after a switch to an atherogenic environment in middle-age mice. *Pflugers Archiv-European Journal of Physiology.* 2013. 465:197-208:

Lesniewski, L. A.,Durrant, J. R.,Connell, M. L.,Henson, G. D.,Black, A. D.,Donato, A. J.,Seals, D. R.. Aerobic exercise reverses arterial inflammation with aging in mice. *American Journal of Physiology - Heart and Circulatory Physiology.* 2011. 301:H1025-H1032:

Lewis, D. Y.,Brett, R. R.. Activity-based anorexia in C57/BL6 mice: effects of the phytocannabinoid, Δ9-tetrahydrocannabinol (THC) and the anandamide analogue, OMDM-2. *European Neuropsychopharmacology.* 2010. 20:622-631:English

Liebetanz, D.,Gerber, J.,Schiffner, C.,Schütze, S.,Klinker, F.,Jarry, H.,Nau, R.,Tauber, S. C.. Pre-infection physical exercise decreases mortality and stimulates neurogenesis in bacterial meningitis. *Journal of Neuroinflammation.* 2012. 9:#pages#:

McGlone, J. J.,Anderson, D. L.,Norman, R. L.. Floor space needs for laboratory mice: BALB/cJ males or females in solid-bottom cages with bedding. *Contemporary Topics in Laboratory Animal Science.* 2001. 40:21-25:English

McMullan, R. C.,Kelly, S. A.,Hua, K.,Buckley, B. K.,Faber, J. E.,Pardo-Manuel de Villena, F.,Pomp, D.. Long-term exercise in mice has sex-dependent benefits on body composition and metabolism during aging. *Physiological Reports.* 2016. 4:11:

McQuaid, R. J.,Audet, M. C.,Jacobson-Pick, S.,Anisman, H.. The differential impact of social defeat on mice living in isolation or groups in an enriched environment: Plasma corticosterone and monoamine variations. *International Journal of Neuropsychopharmacology.* 2013. 16:351-363:

Mitani, K.. SUPPRESSION OF LEVER-PRESSING BEHAVIOR IN RATS REARED IN DIFFERENTIALLY ENRICHED ENVIRONMENT. *Japanese Psychological Research.* 1993. 35:119-128:

Morrow, N. S.,Schall, M.,Grijalva, C. V.,Geiselman, P. J.,Garrick, T.,Nuccion, S.,Novin, D.. Body temperature and wheel running predict survival times in rats exposed to activity-stress. *Physiology & Behavior.* 1997. 62:815-825:English

Nilsson, M. I.,Bourgeois, J. M.,Nederveen, J. P.,Leite, M. R.,Hettinga, B. P.,Bujak, A. L.,May, L.,Lin, E.,Crozier, M.,Rusiecki, D. R.,Moffatt, C.,Azzopardi, P.,Young, J.,Yang, Y.,Nguyen, J.,Adler, E.,Lan, L.,Tarnopolsky, M. A.. Lifelong aerobic exercise protects against inflammaging and cancer. *PLoS ONE*. 2019. 14:#pages#:

Noguchi, T.,Sasaki, Y.,Seki, J.,Giddings, J. C.,Yamamoto, J.. Effects of voluntary exercise and smallcap˜L-arginine on thrombogenesis and microcirculation in stroke-prone spontaneously hypertensive rats. *Clinical and Experimental Pharmacology and Physiology*. 1999. 26:330-335:English

Padilla, J.,Ramirez-Perez, F. I.,Habibi, J.,Bostick, B.,Aroor, A. R.,Hayden, M. R.,Jia, G.,Garro, M.,Demarco, V. G.,Manrique, C.,Booth, F. W.,Martinez-Lemus, L. A.,Sowers, J. R.. Regular exercise reduces endothelial cortical stiffness in western diet-fed female mice. *Hypertension*. 2016. 68:1236-1244:

Paez-Martinez, N.,Flores-Serrano, Z.,Ortiz-Lopez, L.,Ramirez-Rodriguez, G.. Environmental enrichment increases doublecortin-associated new neurons and decreases neuronal death without modifying anxiety-like behavior in mice chronically exposed to toluene. *Behavioural Brain Research*. 2013. 256:432-440:

Possamai, F.,dos Santos, J.,Walber, T.,Marcon, J. C.,dos Santos, T. S.,Lino de Oliveira, C.. Influence of enrichment on behavioral and neurogenic effects of antidepressants in Wistar rats submitted to repeated forced swim test. *Progress in Neuro-Psychopharmacology & Biological Psychiatry*. 2015. 58:15-21:

Rawat, A.,Guo, J.,Renoir, T.,Pang, T. Y.,Hannan, A. J.. Hypersensitivity to sertraline in the absence of hippocampal 5-HT1AR and 5-HTT gene expression changes following paternal corticosterone treatment. *Environmental Epigenetics*. 2018. 4:#pages#:

Rockman, G. E.,Glavin, G. B.. Activity stress effects on voluntary ethanol consumption, mortality and ulcer development in rats. *Pharmacology, Biochemistry and Behavior*. 1986. 24:869-873:

Rowntree, Sharon Ruth. Basic fibroblast growth factor in the injured brain. *#journal#*. 1996. MM18000:140:English

Thomson, S. L.,Garland, T.,Swallow, J. G.,Carter, P. A.. Response of Sod-2 enzyme activity to selection for high voluntary wheel running. *Heredity*. 2002. 88:52-61:

Trott, D. W.,Henson, G. D.,Ho, M. H. T.,Allison, S. A.,Lesniewski, L. A.,Donato, A. J.. Age-related arterial immune cell infiltration in mice is attenuated by caloric restriction or voluntary exercise. *Experimental Gerontology*. 2018. 109:99-107:English

Valdez, G.,Tapia, J. C.,Kang, H.,Clemenson, G. D., Jr.,Gage, F. H.,Lichtman, J. W.,Sanes, J. R.. Attenuation of age-related changes in mouse neuromuscular synapses by caloric restriction and exercise. *Proceedings of the National Academy of Sciences of the United States of America*. 2010. 107:14863-8:

Watanabe, K.,Hara, C.,Ogawa, N.. Feeding conditions and estrous cycle of female rats under the activity-stress procedure from aspects of anorexia nervosa. *Physiology & Behavior*. 1992. 51:827-832:English

Weaver, S. R.,Cronick, C. M.,Prichard, A. P.,Laporta, J.,Benevenga, N. J.,Hernandez, L. L.. Use of

the RatLoft decreases pup mortality in lactating mice. *Laboratory Animals.* 2016. 50:370-378:English

Witt-Lajeunesse, Alane. Effects of behavioral therapies and pharmacological intervention in brain damage. *#journal#.* 2001. MQ68351:127:English

Xu, Z.,Hou, B.,Zhang, Y.,Gao, Y.,Wu, Y.,Zhao, S.,Zhang, C.. Antidepressive behaviors induced byenriched environment might be modulated by glucocorticoid levels. *European Neuropsychopharmacology.* 2009. 19:868-875:

Yang, J.,Li, W.,Liu, X.,Li, Z.,Li, H.,Yang, G.,Xu, L.,Li, L.. Enriched environment treatment counteracts enhanced addictive and depressive-like behavior induced by prenatal chronic stress. *Brain Research.* 2006. 1125:132-7:

Yildirim, E.,Erol, K.,Ulupinar, E.. Effects of sertraline on behavioral alterations caused by environmental enrichment and social isolation. *Pharmacology Biochemistry and Behavior.* 2012. 101:278-287:

Zubedat, S.,Aga-Mizrachi, S.,Cymerblit-Sabba, A.,Ritter, A.,Nachmani, M.,Avital, A.. Methylphenidate and environmental enrichment ameliorate the deleterious effects of prenatal stress on attention functioning. *Stress.* 2015. 18:280-288:

The article does not use one of the relevant measures.

Chen, Y.,Mao, Y.,Zhou, D.,Hu, X.,Wang, J.,Ma, Y.. Environmental enrichment and chronic restraint stress in ICR mice: Effects on prepulse inhibition of startle and Y-maze spatial recognition memory. *Behavioural Brain Research.* 2010. 212:49-55:

Clark, P. J.,Amat, J.,McConnell, S. O.,Ghasem, P. R.,Greenwood, B. N.,Maier, S. F.,Fleshner, M.. Running reduces uncontrollable stress-evoked serotonin and potentiates stress-evoked dopamine concentrations in the rat dorsal striatum. *PLoS ONE.* 2015. 10:#pages#:

Clark, P. J.,Ghasem, P. R.,Mika, A.,Daya, H. E.,Herrera, J. J.,Greenwood, B. N.,Fleshner, M.. Wheel running alters patterns of uncontrollable stress-induced cfos mRNA expression in rat dorsal striatum direct and indirect pathways: A possible role for plasticity in adenosine receptors. *Behavioural Brain Research.* 2014. 272:252-263:

Cook, M. D.,Martin, S. A.,Williams, C.,Whitlock, K.,Wallig, M. A.,Pence, B. D.,Woods, J. A.. Forced treadmill exercise training exacerbates inflammation and causes mortality while voluntary wheel training is protective in a mouse model of colitis. *Brain, Behavior, and Immunity.* 2013. 33:46-56:

Dahlqvist, P.,Rönnbäck, A.,Risedal, A.,Nergårdh, R.,Johansson, I. M.,Seckl, J. R.,Johansson, B. B.,Olsson, T.. Effects of postischemic environment on transcription factor and serotonin receptor expression after permanent focal cortical ischemia in rats. *Neuroscience.* 2003. 119:643-652:

Daneryd, P. L. E.,Hafström, L. R.,Karlberg, I. H.. Effects of spontaneous physical exercise on experimental cancer anorexia and cachexia. *European Journal of Cancer and Clinical Oncology.* 1990. 26:1083-1088:

de Sousa, A. A.,dos Reis, R. R.,de Lima, C. M.,de Oliveira, M. A.,Fernandes, T. N.,Gomes, G. F.,Diniz, D. G.,Magalhães, N. M.,Diniz, C. G.,Sosthenes, M. C. K.,Bento-Torres, J.,Diniz, J. A. P.,Vasconcelos, P. F. C.,Diniz, C. W. P.. Three-dimensional morphometric analysis of microglial changes in a mouse model of virus encephalitis: Age and environmental influences. *European Journal of Neuroscience.* 2015. 42:2036-2050:

de Sousa, A. A.,Reis, R.,Bento-Torres, J.,Trévia, N.,Lins, N. A. A.,Passos, A.,Santos, Z.,Diniz, J. A. P.,Vasconcelos, P. F. C.,Cunningham, C.,Perry, V. H.,Diniz, C. W. P.. Influence of enriched environment on viral encephalitis outcomes: Behavioral and neuropathological changes in albino Swiss mice. *PLoS ONE.* 2011. 6:#pages#:

Esser, K. A.,Harpole, C. E.,Prins, G. S.,Diamond, A. M.. Physical activity reduces prostate carcinogenesis in a transgenic model. *Prostate.* 2009. 69:1372-1377:

Fuller, A. K.,Bice, B. D.,Venancio, A. R.,Crowley, O. M.,Staab, A. M.,Georges, S. J.,Hidalgo, J. R.,Warncke, A. V.,Angus-Hill, M. L.. A Method to Define the Effects of Environmental Enrichment on Colon Microbiome Biodiversity in a Mouse Colon Tumor Model. *Journal of visualized experiments : JoVE.* 2018. #volume#:#pages#:

Gapp, K.,Bohacek, J.,Grossmann, J.,Brunner, A. M.,Manuella, F.,Nanni, P.,Mansuy, I. M.. Potential of environmental enrichment to prevent transgenerational effects of paternal trauma. *Neuropsychopharmacology.* 2016. 41:2749-2758:

Gerecke, K. M.,Kolobova, A.,Allen, S.,Fawer, J. L.. Exercise protects against chronic restraint stress-induced oxidative stress in the cortex and hippocampus. *Brain Research.* 2013. 1509:66-78:

Goes, T. C.,Antunes, F. D.,Teixeira-Silva, F.. Environmental enrichment for adult rats: Effects on trait and state anxiety. *Neuroscience Letters.* 2015. 584:93-96:

Goh, J.,Endicott, E.,Ladiges, W. C.. Pre-tumor exercise decreases breast cancer in old mice in a distance-dependent manner. *American Journal of Cancer Research.* 2014. 4:378-384:

Gómez-Galán, M.,Femenía, T.,. Running opposes the effects of social isolation on synaptic plasticity and transmission in a rat model of depression. *PLoS ONE.* 2016. 11:#pages#:

Greenwood, B. N.,Foley, T. E.,Burhans, D.,Maier, S. F.,Fleshner, M.. The consequences of uncontrollable stress are sensitive to duration of prior wheel running. *Brain Research.* 2005. 1033:164-178:

Greenwood, B. N.,Foley, T. E.,Day, H. E. W.,Campisi, J.,Hammack, S. H.,Campeau, S.,Maier, S. F.,Fleshner, M.. Freewheel running prevents learned helplessness/behavioral depression: Role of dorsal raphe serotonergic neurons. *Journal of Neuroscience.* 2003. 23:2889-2898:

Greenwood, B. N.,Kennedy, S.,Smith, T. P.,Campeau, S.,Day, H. E. W.,Fleshner, M.. Voluntary freewheel running selectively modulates catecholamine content in peripheral tissue and c-Fos expression in the central sympathetic circuit following exposure to uncontrollable stress in rats. *Neuroscience.* 2003. 120:269-281:

Greenwood, B. N.,Loughridge, A. B.,Sadaoui, N.,Christianson, J. P.,Fleshner, M.. The protective effects of voluntary exercise against the behavioral consequences of uncontrollable stress persist despite an increase in anxiety following forced cessation of exercise. *Behavioural Brain Research.* 2012.

233:314-321:

Greenwood, B. N.,Strong, P. V.,Brooks, L.,Fleshner, M.. Anxiety-like behaviors produced by acute fluoxetine administration in male Fischer 344 rats are prevented by prior exercise. *Psychopharmacology.* 2008. 199:209-222:

Greenwood, B. N.,Strong, P. V.,Dorey, A. A.,Fleshner, M.. Therapeutic Effects of Exercise: Wheel Running Reverses Stress-Induced Interference With Shuttle Box Escape. *Behavioral Neuroscience.* 2007. 121:992-1000:

Greenwood, B. N.,Strong, P. V.,Foley, T. E.,Thompson, R. S.,Fleshner, M.. Learned helplessness is independent of levels of brain-derived neurotrophic factor in the hippocampus. *Neuroscience.* 2007. 144:1193-1208:

Gurfein, B. T.,Hasdemir, B.,Milush, J. M.,Touma, C.,Palme, R.,Nixon, D. F.,Darcel, N.,Hecht, F. M.,Bhargava, A.. Enriched environment and stress exposure influence splenic B lymphocyte composition. *PLoS ONE.* 2017. 12:#pages#:

Haack, D.,Luu, H.,Cho, J.,Chen, M. J.,Russo-Neustadt, A.. Exercise reverses chronic stress-induced Bax oligomer formation in the cerebral cortex. *Neuroscience Letters.* 2008. 438:290-294:

Hase, Y.,Craggs, L.,Hase, M.,Stevenson, W.,Slade, J.,Chen, A.,Liang, D.,Ennaceur, A.,Oakley, A.,Ihara, M.,Horsburgh, K.,Kalaria, R. N.. The effects of environmental enrichment on white matter pathology in a mouse model of chronic cerebral hypoperfusion. *Journal of Cerebral Blood Flow and Metabolism.* 2018. 38:151-165:

Hase, Y.,Craggs, L.,Hase, M.,Stevenson, W.,Slade, J.,Lopez, D.,Mehta, R.,Chen, A.,Liang, D.,Oakley, A.,Ihara, M.,Horsburgh, K.,Kalaria, R. N.. Effects of environmental enrichment on white matter glial responses in a mouse model of chronic cerebral hypoperfusion. *Journal of Neuroinflammation.* 2017. 14:#pages#:

Hendricks, S.,Ojuka, E.,Kellaway, L. A.,Mabandla, M. V.,Russell, V. A.. Effect of maternal separation on mitochondrial function and role of exercise in a rat model of Parkinson's disease. *Metabolic Brain Disease.* 2012. 27:387-392:

Hendriksen, H.,Bink, D. I.,Daniels, E. G.,Pandit, R.,Piriou, C.,Slieker, R.,Westphal, K. G. C.,Olivier, B.,Oosting, R. S.. Re-exposure and environmental enrichment reveal NPY-Y1 as a possible target for post-traumatic stress disorder. *Neuropharmacology.* 2012. 63:733-742:

Hendriksen, H.,Meulendijks, D.,Douma, T. N.,Bink, D. I.,Breuer, M. E.,Westphal, K. G. C.,Olivier, B.,Oosting, R. S.. Environmental enrichment has antidepressant-like action without improving learning and memory deficits in olfactory bulbectomized rats. *Neuropharmacology.* 2012. 62:270-277:

Hescham, S.,Grace, L.,Kellaway, L. A.,Bugarith, K.,Russell, V. A.. Effect of exercise on synaptophysin and calcium/calmodulin-dependent protein kinase levels in prefrontal cortex and hippocampus of a rat model of developmental stress. *Metabolic Brain Disease.* 2009. 24:701-709:

Hoffman-Goetz, L.,MacNeil, B.,Arumugam, Y.. Tissue distribution of radiolabelled tumor cells in wheel exercised and sedentary mice. *International Journal of Sports Medicine.* 1994. 15:249-253:

Hong, Suzi. The effect of voluntary wheel running on natural killer cell cytotoxicity after olfactory

bulbectomy. *#journal#.* 2000. 0802633:#pages#:English

Ingram, D. K.,Reynolds, M. A.,Goodrick, C. L.. Relationship of sex, exercise, and growth rate to life span in the Wistar rat: a multivariate correlational approach. *Gerontology.* 1982. 28:23-31:English

Johansson, B. B.,Zhao, L. R.,Mattsson, B.. Environmental influence on neurotrophic gene expression after experimental brain infarction in the rat. *Maturation Phenomenon in Cerebral Ischemia Iii: Defensive Mechanisms Versus Apoptosis - Neuronal Recovery and Protection in Cerebral Infarction.* 1999. #volume#:261-266:

Kalogeraki, E.,Pielecka-Fortuna, J.,Hüppe, J. M.,Löwel, S.. Physical exercise preserves adult visual plasticity in mice and restores it after a stroke in the somatosensory cortex. *Frontiers in Aging Neuroscience.* 2016. 8:#pages#:

Ke, Z.,Yip, S. P.,Li, L.,Zheng, X. X.,Tam, W. K.,Tong, K. Y.. The effects of voluntary, involuntary, and forced exercises on motor recovery in a stroke rat model. *#journal#.* 2011. #volume#:8223-8226:

Khalaji, S.,Bigdeli, I.,Ghorbani, R.,Miladi-Gorji, H.. Research Paper: Environmental enrichment attenuates morphine-induced conditioned place preference and locomotor sensitization in maternally separated rat pups. *Basic and Clinical Neuroscience.* 2018. 9:181-190:

Kumar, R. S.,Narayanan, S. N.,Kumar, N.,Nayak, S.. Exposure to Enriched Environment Restores Altered Passive Avoidance Learning and Ameliorates Hippocampal Injury in Male Albino Wistar Rats Subjected to Chronic Restraint Stress. *International Journal of Applied & Basic Medical Research.* 2018. 8:231-236:

Langbein, H.,Hofmann, A.,Brunssen, C.,Goettsch, W.,Morawietz, H.. Impact of high-fat diet and voluntary running on body weight and endothelial function in LDL receptor knockout mice. *Atherosclerosis Supplements.* 2015. 18:59-66:

Liu, D.,Jiang, X. Y.,Zhou, L. S.. Enriched environment on the intestinal mucosal barrier and brain–gut axis in rats with colorectal cancer. *Experimental Biology and Medicine.* 2018. 243:1185-1198:

Lu, Y. P.,Lou, Y. R.,Nolan, B.,Peng, Q. Y.,Xie, J. G.,Wagner, G. C.,Conney, A. H.. Stimulatory effect of voluntary exercise or fat removal (partial lipectomy) on apoptosis in the skin of UVB light-irradiated mice. *Proceedings of the National Academy of Sciences of the United States of America.* 2006. 103:16301-16306:

MacNeil, B.,Hoffman-Goetz, L.. Chronic exercise enhances in vivo and in vitro cytotoxic mechanisms of natural immunity in mice. *Journal of Applied Physiology.* 1993. 74:388-395:

Marianno, P.,Abrahao, K. P.,Camarini, R.. Environmental enrichment blunts ethanol consumption after restraint stress in C57BL/6 mice. *PLoS ONE.* 2017. 12:e0170317:English

McQuaid, R. J.,Audet, M. C.,Jacobson-Pick, S.,Anisman, H.. Environmental enrichment influences brain cytokine variations elicited by social defeat in mice. *Psychoneuroendocrinology.* 2013. 38:987-996:

Melton, Christopher D.. Environmental enrichment preserves social odor preference and object recognition performance in maternally separated CD-1 mice. *#journal#.* 2014. 1561263:51:English

Mileva, G. R.,Moyes, C.,Syed, S.,Bielajew, C.. Strain Differences and Effects of Environmental

Manipulation on Astrocytes (Glial Fibrillary Acidic Protein), Glucocorticoid Receptor, and Microglia (Iba1) Immunoreactivity between Wistar-Kyoto and Wistar Females. *Neuropsychobiology.* 2017. 75:1-11:

Mirescu, Christian. The lasting effects of early life experience on adult neurogenesis. *#journal#.* 2004. 3123443:136:English

Mitra, R.,Sapolsky, R. M.. Effects of enrichment predominate over those of chronic stress on fear-related behavior in male rats. *Stress.* 2009. 12:305-312:

Mohammadian, J.,Najafi, M.,Miladi-Gorji, H.. Effect of enriched environment during adolescence on spatial learning and memory, and voluntary consumption of morphine in maternally separated rats in adulthood. *Developmental Psychobiology.* 2019. 61:615-625:

Mohtashami Borzadaran, F.,Joushi, S.,Taheri Zadeh, Z.,Sheibani, V.,Esmaeilpour, K.. Environmental enrichment and pain sensitivity; a study in maternally separated rats. *International Journal of Developmental Neuroscience.* 2020. #volume#:#pages#:

Moradi-Kor, N.,Ghanbari, A.,Rashidipour, H.,Yousefi, B.,Bandegi, A. R.,Rashidy-Pour, A.. Beneficial effects of Spirulina platensis, voluntary exercise and environmental enrichment against adolescent stress induced deficits in cognitive functions, hippocampal BDNF and morphological remolding in adult female rats. *Hormones and Behavior.* 2019. 112:20-31:

Morley-Fletcher, S.,Rea, M.,Maccari, S.,Laviola, G.. Environmental enrichment during adolescence reverses the effects of prenatal stress on play behaviour and HPA axis reactivity in rats. *European Journal of Neuroscience.* 2003. 18:3367-3374:

Murphy, M. O.,Petriello, M. C.,Han, SungGu,Sunkara, M.,Morris, A. J.,Esser, K.,Hennig, B.. Exercise protects against PCB-induced inflammation and associated cardiovascular risk factors. *Environmental Science and Pollution Research.* 2016. 23:2201-2211:English

Nakajima, S.,Ohsawa, I.,Ohta, S.,Ohno, M.,Mikami, T.. Regular voluntary exercise cures stress-induced impairment of cognitive function and cell proliferation accompanied by increases in cerebral IGF-1 and GST activity in mice. *Behavioural Brain Research.* 2010. 211:178-184:

Novati, A.,Hentrich, T.,Wassouf, Z.,Weber, J. J.,Yu-Taeger, L.,Déglon, N.,Nguyen, H. P.,Schulze-Hentrich, J. M.. Environment-dependent striatal gene expression in the BACHD rat model for Huntington disease. *Scientific Reports.* 2018. 8:#pages#:

Overton, J. M.,Tipton, C. M.,Matthes, R. D.,Leininger, J. R.. Voluntary exercise and its effects on young SHR and stroke-prone hypertensive rats. *Journal of Applied Physiology.* 1986. 61:318-324:

Patel, D. I.,Abuchowski, K.,Bedolla, R.,Rivas, P.,Musi, N.,Reddick, R.,Kumar, A. P.. Nexrutine and exercise similarly prevent high grade prostate tumors in transgenic mouse model. *PLoS ONE.* 2019. 14:#pages#:

Pettan-Brewer, C.,Goh, J.,Ladiges, W. C.. An immunohistochemical approach for monitoring effects of exercise on tumor stromal cells in old mice. *Pathobiology of Aging & Age Related Diseases.* 2014. 4:#pages#:

Rakhshani, Nasimeh. Effects of short- and long-term voluntary exercise training on diurnal rhythm, the acute stress response and adrenal sensitivity in male Sprague-Dawley rats. *#journal#.* 2006.

MR19754:113:English

Richter, S. H.,Schick, A.,Hoyer, C.,Lankisch, K.,Gass, P.,Vollmayr, B.. A glass full of optimism: Enrichment effects on cognitive bias in a rat model of depression. *Cognitive, Affective and Behavioral Neuroscience.* 2012. 12:527-542:

Richter, S. H.,Zeuch, B.,Riva, M. A.,Gass, P.,Vollmayr, B.. Environmental enrichment ameliorates depressive-like symptoms in young rats bred for learned helplessness. *Behavioural Brain Research.* 2013. 252:287-292:

Robertson, J. M.,Achua, J. K.,Smith, J. P.,Prince, M. A.,Staton, C. D.,Ronan, P. J.,Summers, T. R.,Summers, C. H.. Anxious behavior induces elevated hippocampal Cb2 receptor gene expression. *Neuroscience.* 2017. 352:273-284:

Roebuck, B. D.,McCaffrey, J.,Baumgartner, K. J.. Protective Effects of Voluntary Exercise during the Postinitiation Phase of Pancreatic Carcinogenesis in the Rat. *Cancer Research.* 1990. 50:6811-6816:

Rossi, S.,De Chiara, V.,Musella, A.,Kusayanagi, H.,Mataluni, G.,Bernardi, G.,Usiello, A.,Centonze, D.. Chronic psychoemotional stress impairs cannabinoid-receptor-mediated control of GABA transmission in the striatum. *Journal of Neuroscience.* 2008. 28:7284-7292:

Rule, L.,Yang, J.,Watkin, H.,Hall, J.,Brydges, N. M.. Environmental enrichment rescues survival and function of adult-born neurons following early life stress. *Molecular Psychiatry.* 2020. #volume#:#pages#:

Sasaki, Y.,Noguchi, T.,Yamamoto, E.,Giddings, J. C.,Ikeda, K.,Yamamoto, J.,Yamori, Y.. Effects of voluntary exercise on cerebral thrombosis AND endothelial function in spontaneously hypertensive rats (shrsp/izm). *Clinical and Experimental Pharmacology and Physiology.* 2004. 31:S47-S48:

Sasse, Sarah Kay. Voluntary exercise facilitates adaptation to repeated stress: Potential neurochemical mediators, effects of exercise duration and stressor intensity, and implications for health and well-being. *#journal#.* 2009. 3366636:202:English

Schneider, A.,Rogalewski, A.,Wafzig, O.,Kirsch, F.,Gretz, N.,Krüger, C.,Diederich, K.,Pitzer, C.,Laage, R.,Plaas, C.,Vogt, G.,Minnerup, J.,Schäbitz, W. R.. Forced arm use is superior to voluntary training for motor recovery and brain plasticity after cortical ischemia in rats. *Experimental and Translational Stroke Medicine.* 2014. 6:#pages#:

Seo, H.,Park, C. H.,Choi, S.,Kim, W.,Jeon, B. D.,Ryu, S.. Effects of voluntary exercise on apoptosis and cortisol after chronic restraint stress in mice. *Journal of Exercise Nutrition & Biochemistry.* 2016. 20:16-23:

Sifonios, L.,Trinchero, M.,Cereseto, M.,Ferrero, A.,Cladouchos, M. L.,Macedo, G. F.,Reinés, A.,Wikinski, S.. An enriched environment restores normal behavior while providing cytoskeletal restoration and synaptic changes in the hippocampus of rats exposed to an experimental model of depression. *Neuroscience.* 2009. 164:929-940:

Sun, Y.,Pence, B. D.,Wang, S. S.,Woods, J. A.. Effects of Exercise on Stress-induced Attenuation of Vaccination Responses in Mice. *Medicine and Science in Sports and Exercise.* 2019. 51:1635-1641:

Talan, M. I.,Ingram, D. K.. Effects of voluntary and forced exercise on thermoregulation and survival in

aged C57BL/6J mice. *Mechanisms of Ageing and Development.* 1986. 36:269-279:

Tanner, M. K.,Fallon, I. P.,Baratta, M. V.,Greenwood, B. N.. Voluntary exercise enables stress resistance in females. *Behavioural Brain Research.* 2019. 369:#pages#:

Tanti, A.,Rainer, Q.,Minier, F.,Surget, A.,Belzung, C.. Differential environmental regulation of neurogenesis along the septo-temporal axis of the hippocampus. *Neuropharmacology.* 2012. 63:374-384:

Tanti, A.,Westphal, W. P.,Girault, V.,Brizard, B.,Devers, S.,Leguisquet, A. M.,Surget, A.,Belzung, C.. Region-dependent and stage-specific effects of stress, environmental enrichment, and antidepressant treatment on hippocampal neurogenesis. *Hippocampus.* 2013. 23:797-811:

Tsuda, A.,Tanaka, M.,Nishikawa, T.,Iimori, K.,Hoaki, Y.,Ida, Y.,Kohno, Y.,Nagaski, N.. Priming effects of activity-stress ulcer in rats. *Physiology and Behavior.* 1982. 29:733-736:

Van Hoomissen, J. D.,Chambliss, H. O.,Holmes, P. V.,Dishman, R. K.. Effects of chronic exercise and imipramine on mRNA for BDNF after olfactory bulbectomy in rat. *Brain Research.* 2003. 974:228-235:

Viidik, A.,Skalicky, M.. Voluntary exercise and mild food restriction effectively retard the collagen biomarker of aging. *Aging Clinical and Experimental Research.* 2003. 15:475-481:

Wang, A.,Nie, W.,Li, H.,Hou, Y.,Yu, Z.,Fan, Q.,Sun, R.. Epigenetic upregulation of corticotrophin-releasing hormone mediates postnatal maternal separation-induced memory deficiency. *PLoS ONE.* 2014. 9:#pages#:

Wolff, G.,Balke, J. E.,Andras, I. E.,Park, M.,Toborek, M.. Exercise modulates redox-sensitive small GTPase activity in the brain microvasculature in a model of brain metastasis formation. *PLoS ONE.* 2014. 9:#pages#:

Yager, J. D., Jr.,Lichtenstein, M. J.,Bonney, R. J.,Hopkins, H. A.,Walker, P. R.,Dorn, C. G.,Potter, V. R.. Effects of various feeding and exercise regimens on rat growth and survival. *Journal of Nutrition.* 1974. 104:273-286:English

Yoo, H. S.,Tackett, R. L.,Crabbe, J. B.,Bunnell, B. N.,Dishman, R. K.. Antidepressant-like effects of physical activity versus imipramine: Neonatal clomipramine model. *Psychobiology.* 2000. 28:540-549:

Zhao, L. R.,Risedal, A.,Wojcik, A.,Hejzlar, J.,Johansson, B. B.,Kokaia, Z.. Enriched environment influences brain-derived neurotrophic factor levels in rat forebrain after focal stroke. *Neuroscience Letters.* 2001. 305:169-72:

Zhao, Li-Ru. Post-ischemic housing conditions influence on gene transcription and translation after permanent focal brain ischemia in rats. *#journal#.* 2004. C817560:71:English

The data was replicated from another publication.

Zhang Y, Xu D, Qi H, Yuan Y, Liu H, Yao S, Yuan S, Zhang J. Enriched environment promotes post-stroke neurogenesis through NF-κB-mediated secretion of IL-17A from astrocytes. Brain Res. 2018 May 15;1687:20-31. doi: 10.1016/j.brainres.2018.02.030. Epub 2018 Feb 23. PMID: 29481794.

Wu X, Liu S, Hu Z, Zhu G, Zheng G, Wang G. Enriched housing promotes post-stroke neurogenesis through calpain 1-STAT3/HIF-1α/VEGF signaling. Brain Res Bull. 2018 May;139:133-143. doi: 10.1016/j.brainresbull.2018.02.018. Epub 2018 Mar 22. PMID: 29477834.

Chen, JY., Yu, Y., Yuan, Y. *et al.* Enriched housing promotes post-stroke functional recovery through astrocytic HMGB1-IL-6-mediated angiogenesis. *Cell Death Discov.* **3,** 17054 (2017). https://doi.org/10.1038/cddiscovery.2017.54
