# Supplementary material for: Conventional laboratory housing increases morbidity and mortality in research rodents: results of a meta-analysis
Source: BMC Biol. 2022 Jan 13;20:15. doi: 10.1186/s12915-021-01184-0 (PMC8756709; doi:10.1186/s12915-021-01184-0)
Supplement: Supplementary file 14 — Additional file 14. Categorization of each resource. [file 12915_2021_1184_MOESM14_ESM.pdf]

**Resource**	**Author description or Clear Photograph**
space	- enriched cage was larger than conventional cage
shelter/hiding opportunities	- shelters, tubes or huts
climbing/3D movement	- ladders<br>- ramps<br>- multi-tiered cages
extra nesting materials/sleeping places	- more nesting material than conventional cage<br>- hammocks<br>- nest boxes
wheel	- running wheel (unlocked)
foraging opportunities	- authors specify or picture shows an opportunity to forage<br>- food was scattered or moved throughout the cage
gnawing/chewing opportunities	- authors specify or picture shows gnawing opportunities<br>- given objects designed to permit gnawing (e.g. wood or other chewable materials)
novelty	- provision of novel objects<br>- rotation/replacement of objects
