# Supplementary material for: Conventional laboratory housing increases morbidity and mortality in research rodents: results of a meta-analysis
Source: BMC Biol. 2022 Jan 13;20:15. doi: 10.1186/s12915-021-01184-0 (PMC8756709; doi:10.1186/s12915-021-01184-0)
Supplement: Supplementary file 16 — Additional file 16. A reference list of studies included in this systematic review. [file 12915_2021_1184_MOESM16_ESM.pdf]

References included in the review

1. Akillioglu K, Yilmaz MB, Boga A, Binokay S, Kocaturk-Sel S. Environmental enrichment does not reverse the effects of maternal deprivation on NMDAR and Balb/c mice behaviors. Brain research. 2015;1624:479-88.
2. Altun M, Bergman E, Edstrom E, Johnson H, Ulfhake B. Behavioral impairments of the aging rat. Physiology & Behavior. 2007;92(5):911-23.
3. Arranz L, De Castro NM, Baeza I, Maté I, Viveros MP, De La Fuente M. Environmental enrichment improves age-related immune system impairment: Long-term exposure since adulthood increases life span in mice. Rejuvenation Research. 2010;13(4):415-28.
4. Avrabos C, Sotnikov SV, Dine J, Markt PO, Holsboer F, Landgraf R, et al. Real-time imaging of amygdalar network dynamics in vitro reveals a neurophysiological link to behavior in a mouse model of extremes in trait anxiety. The Journal of neuroscience : the official journal of the Society for Neuroscience. 2013;33(41):16262-7.
5. Benaroya-Milshtein N, Apter A, Yaniv I, Kukulansky T, Raz N, Haberman Y, et al. Environmental enrichment augments the efficacy of idiotype vaccination for B-cell lymphoma. Journal of immunotherapy (Hagerstown, Md : 1997). 2007;30(5):517-22.
6. Bernberg E, Andersson IJ, Gan LM, Naylor AS, Johansson ME, Bergström G. Effects of social isolation and environmental enrichment on atherosclerosis in ApoE-/- mice. Stress. 2008;11(5):381-9.
7. Betof AS. Therapeutic Aerobic Exercise in a Mouse Model of Breast Cancer: Effects on Tumor Progression, Angiogenesis, and Response to Chemotherapy [Ph.D.]. Ann Arbor: Duke University; 2012.
8. Betof AS, Lascola CD, Weitzel D, Landon C, Scarbrough PM, Devi GR, et al. Modulation of Murine Breast Tumor Vascularity, Hypoxia, and Chemotherapeutic Response by Exercise. Jnci-Journal of the National Cancer Institute. 2015;107(5).
9. Bhansali P, Dunning J, Singer SE, David L, Schmauss C. Early life stress alters adult serotonin 2C receptor pre-mRNA editing and expression of the α subunit of the heterotrimeric G-protein Gq. Journal of Neuroscience. 2007;27(6):1467-73.
10. Bice BD, Stephens MR, Georges SJ, Venancio AR, Bermant PC, Warncke AV, et al. Environmental Enrichment Induces Pericyte and IgA-Dependent Wound Repair and Lifespan Extension in a Colon Tumor Model. Cell Reports. 2017;19(4):760-73.
11. Bjørnebekk A, Mathé AA, Brené S. The antidepressant effect of running is associated with increased hippocampal cell proliferation. International Journal of Neuropsychopharmacology. 2005;8(3):357-68.
12. Briones TL. The effects of environment on enhancing functional plasticity following cerebral ischemia [Ph.D.]. Ann Arbor: University of Michigan; 1997.
13. Bronikowski AM, Morgan TJ, Garland T, Jr., Carter PA. The evolution of aging and age-related physical decline in mice selectively bred for high voluntary exercise. Evolution. 2006;60(7):1494-508.
14. Buss LA, Dachs GU. Voluntary exercise slows breast tumor establishment and reduces tumor hypoxia in ApoE^{-/-} mice. Journal of Applied Physiology. 2018;124(4):938-49.
15. Buss LA, Ang AD, Hock B, Robinson BA, Currie MJ, Dachs GU. Effect of post-implant exercise on tumour growth rate, perfusion and hypoxia in mice. PLoS ONE. 2020;15(3).
16. Cao L, Liu X, Lin EJD, Wang C, Choi EY, Riban V, et al. Environmental and Genetic Activation of a Brain-Adipocyte BDNF/Leptin Axis Causes Cancer Remission and Inhibition. Cell. 2010;142(1):52-64.
17. Cesar L, Suarez SV, Adi J, Adi N, Vazquez-Padron R, Yu H, et al. An Essential Role for Diet in Exercise-Mediated Protection against Dyslipidemia, Inflammation and Atherosclerosis in ApoE-/- Mice. Plos One. 2011;6(2).
18. Chambliss HO, Van Hoomissen JD, Holmes PV, Bunnell BN, Dishman RK. Effects of chronic activity wheel running and imipramine on masculine copulatory behavior after olfactory bulbectomy. Physiology and Behavior. 2004;82(4):593-600.
19. Chekmareva NY, Sotnikov SV, Diepold RP, Naik RR, Landgraf R, Czibere L. Environmental manipulations generate bidirectional shifts in both behavior and gene regulation in a crossbred mouse model of extremes in trait anxiety. Frontiers in Behavioral Neuroscience. 2014;8:87.
20. Chen X, Zhang X, Liao W, Wan Q. Effect of Physical and Social Components of Enriched Environment on Astrocytes Proliferation in Rats After Cerebral Ischemia/Reperfusion Injury. Neurochemical Research. 2017;42(5):1308-16.
21. Chen X, Zhang X, Xue L, Hao C, Liao W, Wan Q. Treatment with Enriched Environment Reduces Neuronal Apoptosis in the Periinfarct Cortex after Cerebral Ischemia/Reperfusion Injury. Cellular Physiology & Biochemistry. 2017;41(4):1445-56.
22. Chen JY, Yu Y, Yuan Y, Zhang YJ, Fan XP, Yuan SY, et al. Enriched housing promotes post-stroke functional recovery through astrocytic HMGB1-IL-6-mediated angiogenesis. Cell Death Discovery. 2017;3:17054.
23. Chun XL, Jiang J, Qi GZ, Xin JZ, Wang W, Zhi JZ, et al. Voluntary exercise-induced neurogenesis in the postischemic dentate gyrus is associated with spatial memory recovery from stroke. Journal of Neuroscience Research. 2007;85(8):1637-46.
24. Colbert LH, Westerlind KC, Perkins SN, Haines DC, Berrigan D, Donehower LA, et al. Exercise effects on tumorigenesis in a p53-deficient mouse model of breast cancer. Medicine and Science in Sports and Exercise. 2009;41(8):1597-605.
25. Dahlqvist P, Zhao L, Johansson IM, Mattsson B, Johansson BB, Seckl JR, et al. Environmental enrichment alters nerve growth factor-induced gene A and glucocorticoid receptor messenger RNA expression after middle cerebral artery occlusion in rats. Neuroscience. 1999;93(2):527-35.
26. De Boer A, Storm A, Gomez-Soler M, Smolders S, Rué L, Poppe L, et al. Environmental enrichment during the chronic phase after experimental stroke promotes functional recovery without synergistic effects of EphA4 targeted therapy. Human Molecular Genetics. 2020;29(4):605-17.

27. De Waard MC, Duncker DJ. Prior exercise improves survival, infarct healing, and left ventricular function after myocardial infarction. Journal of Applied Physiology. 2009;107(3):928-36.
28. Diniz DG, Foro CA, Turiel MC, Sosthenes MC, Demachki S, Gomes GF, et al. Environmental influences on antibody-enhanced dengue disease outcomes. Memorias do Instituto Oswaldo Cruz. 2012;107(8):1021-9.
29. Diniz DG, Foro CAR, Sosthenes MCK, Demachki S, Gomes GF, Malerba GA, et al. Aging and environmental enrichment exacerbate inflammatory response on antibody-enhanced dengue disease in immunocompetent murine model. European Journal of Inflammation. 2013;11(3):719-31.
30. Elisia I, Cho B, Hay M, Li Y, Hofs E, Lam V, et al. The effect of diet and exercise on tobacco carcinogen-induced lung cancer. Carcinogenesis. 2019;40(3):448-60.
31. Farokhi-Sisakht F, Sadigh-Eteghad S, Mohaddes G, Ebrahimi-Kalan A, Karimi P, Farhoudi M. Physical and cognitive training attenuate hippocampal ischemia-induced memory impairments in rat. Brain Research Bulletin. 2019.
32. Foglesong G. Lifestyle Improvements Enhance Metabolic Function and Mitigate Breast Cancer Progression [Ph.D.]. Ann Arbor: The Ohio State University; 2017.
33. Foglesong GD, Queen NJ, Huang W, Widstrom KJ, Cao L. Enriched environment inhibits breast cancer progression in obese models with intact leptin signaling. Endocrine-Related Cancer. 2019;26(5):483-95.
34. Foley JM, Stark KD, Zajchowski S, Meckling KA. Fatty acids and exercise affect glucose transport but not tumour growth in F-344 rats. Canadian Journal of Applied Physiology. 2004;29(5):604-22.
35. Geibig CS, Keiner S, Redecker C. Functional recruitment of newborn hippocampal neurons after experimental stroke. Neurobiology of Disease. 2012;46(2):431-9.
36. Gertz K, Priller J, Kronenberg G, Fink KB, Winter B, Schröck H, et al. Physical activity improves long-term stroke outcome via endothelial nitric oxide synthase-dependent augmentation of neovascularization and cerebral blood flow. Circulation Research. 2006;99(10):1132-40.
37. Ghalandari-Shamami M, Nourizade S, Yousefi B, Vafaei AA, Pakdel R, Rashidy-Pour A. Beneficial effects of physical activity and crocin against adolescent stress induced anxiety or depressive-like symptoms and dendritic morphology remodeling in prefrontal cortex in adult male rats. Neurochemical Research. 2019;44(4):917-29.
38. Giles TC, Roebuck BD. Effects of voluntary exercise and/or food restriction on pancreatic tumorigenesis in male rats. Advances in Experimental Medicine and Biology. 1992;322:17-27.
39. Gobinath AR, Richardson RJ, Chow C, Workman JL, Lieblich SE, Barr AM, et al. Voluntary running influences the efficacy of fluoxetine in a model of postpartum depression. Neuropharmacology. 2018;128:106-18.
40. Goh J. Effects of Voluntary Running on Inflammation and Tumor Progression in Mice [Ph.D.]. Ann Arbor: University of Washington; 2012.
41. Goh J, Tsai J, Bammler TK, Farin FM, Endicott E, Ladiges WC. Exercise training in transgenic mice is associated with attenuation of early breast cancer growth in a dose-dependent manner. PLoS ONE. 2013;8(11).
42. Gomes GF, Peixoto RDDF, Maciel BG, Santos KFD, Bayma LR, Feitoza Neto PA, et al. Differential Microglial Morphological Response, TNFα, and Viral Load in Sedentary-like and Active Murine Models After Systemic Non-neurotropic Dengue Virus Infection. Journal of Histochemistry and Cytochemistry. 2019;67(6):419-39.
43. Gonçalves LV, Herlinger AL, Ferreira TAA, Coitinho JB, Pires RGW, Martins-Silva C. Environmental enrichment cognitive neuroprotection in an experimental model of cerebral ischemia: biochemical and molecular aspects. Behavioural Brain Research. 2018;348:171-83.
44. González-Pardo H, Arias JL, Vallejo G, Conejo NM. Environmental enrichment effects after early stress on behavior and functional brain networks in adult rats. PLoS ONE. 2019;14(12).
45. González-Pardo H, Arias JL, Vallejo G, Conejo NM. Influence of environmental enrichment on the volume of brain regions sensitive to early life stress by maternal separation in rats. Psicothema. 2019;31(1):46-52.
46. Goodrick CL. Effects of long-term voluntary wheel exercise on male and female Wistar rats. I. Longevity, body weight, and metabolic rate. Gerontology. 1980;26(1):22-33.
47. Grace L, Hescham S, Kellaway LA, Bugarith K, Russell VA. Effect of exercise on learning and memory in a rat model of developmental stress. Metabolic brain disease. 2009;24(4):643-57.
48. Hase Y, Polvikoski TM, Ihara M, Hase M, Zafar R, Stevenson W, et al. Carotid artery disease in post-stroke survivors and effects of enriched environment on stroke pathology in a mouse model of carotid artery stenosis. Neuropathology and Applied Neurobiology. 2019;45(7):681-97.
49. Higgins KA, Park D, Lee GY, Curran WJ, Deng X. Exercise-induced lung cancer regression: Mechanistic findings from a mouse model. Cancer. 2014;120(21):3302-10.
50. Hoffman-Goetz L, May KM, Arumugam Y. Exercise training and mouse mammary tumour metastasis. Anticancer Research. 1994;14(6 B):2627-31.
51. Holloszy JO, Smith EK, Vining M, Adams S. Effect of voluntary exercise on longevity of rats. Journal of Applied Physiology: Respiratory, Environmental and Exercise Physiology. 1985;59(3):826-31.
52. Holloszy JO, Schechtman KB. Interaction between exercise and food restriction: effects on longevity of male rats. Journal of Applied Physiology. 1991;70(4):1529-35.
53. Holloszy JO. Exercise increases average longevity of female rats despite increased food intake and no growth retardation. Journals of Gerontology. 1993;48(3):B97-B100.
54. Holloszy JO. Mortality rate and longevity of food-restricted exercising male rats: A reevaluation. Journal of Applied Physiology. 1997;82(2):399-403.
55. Holloszy JO. Longevity of exercising male rats: effect of an antioxidant supplemented diet. Mechanisms of Ageing and Development. 1998;100(3):211-9.

56. Hui JJ, Zhang ZJ, Liu SS, Xi GJ, Zhang XR, Teng GJ, et al. Hippocampal neurochemistry is involved in the behavioural effects of neonatal maternal separation and their reversal by post-weaning environmental enrichment: A magnetic resonance study. Behavioural Brain Research. 2011;217(1):122-7.
57. Hutton CP, Déry N, Rosa E, Lemon JA, Rollo CD, Boreham DR, et al. Synergistic effects of diet and exercise on hippocampal function in chronically stressed mice. Neuroscience. 2015;308:180-93.
58. Ikuyama T, Watanabe T, Minegishi Y, Osanai H. EFFECT OF VOLUNTARY EXERCISE ON 3'-METHYL-4-DIMETHYLAMINOAZOBENZENE-INDUCED HEPATOMAS IN MALE JC1-WISTAR RATS. Proceedings of the Society for Experimental Biology and Medicine. 1993;204(2):211-5.
59. Ilin Y, Richter-Levin G. Enriched environment experience overcomes learning deficits and depressive-like behavior induced by juvenile stress. PLoS One. 2009;4(1):e4329.
60. Ishikawa J, Ogawa Y, Owada Y, Ishikawa A. Hyperlocomotor activity and stress vulnerability during adulthood induced by social isolation after early weaning are prevented by voluntary running exercise before normal weaning period. Behav Brain Res. 2014;264:197-206.
61. Iwata E, Kikusui T, Takeuchi Y, Mori Y. Fostering and environmental enrichment ameliorate anxious behavior induced by early weaning in Balb/c mice. Physiol Behav. 2007;91(2-3):318-24.
62. Jiang F, Li WP, Kwiecien J, Turnbull J. A study of the purine derivative AIT-082 in G93A SOD1 transgenic mice. International Journal of Immunopathology and Pharmacology. 2006;19(3):489-98.
63. Jiang W, Zhu Z, Thompson HJ. Effects of physical activity and restricted energy intake on chemically induced mammary carcinogenesis. Cancer Prevention Research. 2009;2(4):338-44.
64. Jiang C, Yu K, Wu Y, Xie H, Liu G, Wu J, et al. Enriched Environment Enhances Poststroke Neurological Function Recovery on Rat: Involvement of p-ERK1/2. Journal of Stroke and Cerebrovascular Diseases. 2016;25(7):1590-8.
65. Johansson BB, Ohlsson AL. Environment, social interaction, and physical activity as determinants of functional outcome after cerebral infarction in the rat. Experimental Neurology. 1996;139(2):322-7.
66. Johansson BB. Functional outcome in rats transferred to an enriched environment 15 days after focal brain ischemia. Stroke. 1996;27(2):324-6.
67. Jones LW, Viglianti BL, Tashjian JA, Kothadia SM, Keir ST, Freedland SJ, et al. Effect of aerobic exercise on tumor physiology in an animal model of human breast cancer. Journal of Applied Physiology. 2010;108(2):343-8.
68. Jones LW, Antonelli J, Masko EM, Broadwater G, Lascola CD, Fels D, et al. Exercise modulation of the host-tumor interaction in an orthotopic model of murine prostate cancer. Journal of Applied Physiology. 2012;113(2):263-72.
69. Ju JY, Nolan B, Cheh M, Bose M, Lin Y, Wagner GC, et al. Voluntary exercise inhibits intestinal tumorigenesis in ApcMin/+ mice and azoxymethane/dextran sulfate sodium-treated mice. BMC Cancer. 2008;8(316):(02 November 2008).
70. Ke Z, Yip SP, Li L, Zheng XX, Tong KY. The effects of voluntary, involuntary, and forced exercises on brain-derived neurotrophic factor and motor function recovery: A rat brain ischemia model. PLoS ONE. 2011;6(2).
71. Keiner S, Wurm F, Kunze A, Witte OW, Redecker C. Rehabilitative therapies differentially alter proliferation and survival of glial cell populations in the perilesional zone of cortical infarcts. GLIA. 2008;56(5):516-27.
72. Kershaw MH, Westwood JA, Darcy PK. Environmental enrichment does not impact on tumor growth in mice. F1000Research. 2013;2.
73. Kim YJ, Lee J, Kang H, Jeon JY. Neurorestorative effect of erythropoietin and environmental enrichment in the early stage of stroke recovery. Animal Cells and Systems. 2016;20(3):165-73.
74. Knieling M, Metz GA, Antonow-Schlorke I, Witte OW. Enriched environment promotes efficiency of compensatory movements after cerebral ischemia in rats. Neuroscience. 2009;163(3):759-69.
75. Komitova M, Mattsson B, Johansson BB, Eriksson PS. Enriched environment increases neural stem/progenitor cell proliferation and neurogenesis in the subventricular zone of stroke-lesioned adult rats. Stroke. 2005;36(6):1278-82.
76. Komitova M, Zhao LR, Gidö G, Johansson BB, Eriksson P. Postischemic exercise attenuates whereas enriched environment has certain enhancing effects on lesion-induced subventricular zone activation in the adult rat. European Journal of Neuroscience. 2005;21(9):2397-405.
77. Lapmanee S, Charoenphandhu J, Charoenphandhu N. Beneficial effects of fluoxetine, reboxetine, venlafaxine, and voluntary running exercise in stressed male rats with anxiety- and depression-like behaviors. Behav Brain Res. 2013;250:316-25.
78. Lapmanee S, Charoenphandhu J, Teerapornpuntakit J, Krishnamra N, Charoenphandhu N. Agomelatine, venlafaxine, and running exercise effectively prevent anxiety- and depression-like behaviors and memory impairment in restraint stressed rats. PLoS One. 2017;12(11):e0187671.
79. Laufs U, Wassmann S, Czech T, Münzel T, Eisenhauer M, Böhm M, et al. Physical inactivity increases oxidative stress, endothelial dysfunction, and atherosclerosis. Arteriosclerosis, Thrombosis, and Vascular Biology. 2005;25(4):809-14.
80. Lehmann ML, Herkenham M. Environmental enrichment confers stress resiliency to social defeat through an infralimbic cortex-dependent neuroanatomical pathway. Journal of Neuroscience. 2011;31(16):6159-73.
81. Li M, Wang M, Ding S, Li C, Luo X. Environmental Enrichment during Gestation Improves Behavior Consequences and Synaptic Plasticity in Hippocampus of Prenatal-Stressed Offspring Rats. Acta histochemica et cytochemica. 2012;45(3):157-66.
82. Li G, Gan Y, Fan Y, Wu Y, Lin H, Song Y, et al. Enriched environment inhibits mouse pancreatic cancer growth and down-regulates the expression of mitochondria-related genes in cancer cells. Scientific Reports. 2015;5:7856.

83. Li MZ, Zhan Y, Yang L, Feng XF, Zou HY, Lei JF, et al. MRI Evaluation of Axonal Remodeling After Combination Treatment With Xiaoshuan Enteric-Coated Capsule and Enriched Environment in Rats After Ischemic Stroke. Frontiers in Physiology. 2019;10.
84. Lou YR, Peng QY, Li T, Nolan B, Bernard JJ, Wagner GC, et al. Oral caffeine during voluntary exercise markedly inhibits skin carcinogenesis and decreases inflammatory cytokines in UVB-treated mice. Nutrition and Cancer. 2013;65(7):1002-13.
85. Macneil B, Hoffman-Goetz L. Effect of exercise on natural cytotoxicity and pulmonary tumor metastases in mice. Medicine and Science in Sports and Exercise. 1993;25(8):922-8.
86. Maloyan A, Gulick J, Glabe CG, Kayed R, Robbins J. Exercise reverses preamyloid oligomer and prolongs survival in αB-crystallin-based desmin-related cardiomyopathy. Proceedings of the National Academy of Sciences of the United States of America. 2007;104(14):5995-6000.
87. Masrour FF, Peeri M, Hosseini MJ, Azarbayjani MA. Exercise during adolescence attenuated depressive-like behaviors and hippocampal mitochondrial dysfunction following early life stress in adult male rats. Iranian Journal of Pharmaceutical Research. 2018;17(Special Issue 2):124-33.
88. Masrour FF, Peeri M, Azarbayjani MA, Hosseini MJ. Voluntary Exercise During Adolescence Mitigated Negative the Effects of Maternal Separation Stress on the Depressive-Like Behaviors of Adult Male Rats: Role of NMDA Receptors. Neurochemical Research. 2018;43(5):1067-74.
89. Matsumoto A, Manthey HD, Marsh SA, Fassett RG, De Haan JB, Rolfe BE, et al. Effects of exercise training and RhoA/ROCK inhibition on plaque in ApoE-/- mice. International Journal of Cardiology. 2013;167(4):1282-8.
90. McCarter RJM, Shimokawa I, Ikeno Y, Higami Y, Hubbard GB, Yu BP, et al. Physical activity as a factor in the action of dietary restriction on aging: Effects in Fisher 344 rats. Aging Clinical and Experimental Research. 1997;9(1-2):73-9.
91. McMurphy T, Huang W, Queen NJ, Ali S, Widstrom KJ, Liu X, et al. Implementation of environmental enrichment after middle age promotes healthy aging. Aging. 2018;10(7):1698-721.
92. Mehl KA, Davis JM, Clements JM, Berger FG, Pena MM, Carson JA. Decreased intestinal polyp multiplicity is related to exercise mode and gender in ApcMin/+ mice. Journal of Applied Physiology. 2005;98(6):2219-25.
93. Meissner M, Lombardo E, Havinga R, Tietge UJF, Kuipers F, Groen AK. Voluntary wheel running increases bile acid as well as cholesterol excretion and decreases atherosclerosis in hypercholesterolemic mice. Atherosclerosis. 2011;218(2):323-9.
94. Meissner M, Wolters H, de Boer RA, Havinga R, Boverhof R, Bloks VW, et al. Bile acid sequestration normalizes plasma cholesterol and reduces atherosclerosis in hypercholesterolemic mice. No additional effect of physical activity. Atherosclerosis. 2013;228(1):117-23.
95. Meng Z, Liu T, Song Y, Wang Q, Xu D, Jiang J, et al. Exposure to an enriched environment promotes the terminal maturation and proliferation of natural killer cells in mice. Brain, Behavior, & Immunity. 2019;77:150-60.
96. Michna L, Wagner GC, Lou YR, Xie JG, Peng QY, Lin Y, et al. Inhibitory effects of voluntary running wheel exercise on UVB-induced skin carcinogenesis in SKH-1 mice. Carcinogenesis. 2006;27(10):2108-15.
97. Mileva GR, Bielajew C. Environmental manipulation affects depressive-like behaviours in female Wistar-Kyoto rats. Behav Brain Res. 2015;293:208-16.
98. Mu JW, Bakreen A, Juntunen M, Korhonen P, Oinonen E, Cui LL, et al. Combined Adipose Tissue-Derived Mesenchymal Stem Cell Therapy and Rehabilitation in Experimental Stroke. Frontiers in Neurology. 2019;10.
99. Mul JD, Soto M, Cahill ME, Ryan RE, Takahashi H, So K, et al. Voluntary wheel running promotes resilience to chronic social defeat stress in mice: a role for nucleus accumbens ΔFosB. Neuropsychopharmacology. 2018;43(9):1934-42.
100. Nachat-Kappes R, Pinel A, Combe K, Lamas B, Farges MC, Rossary A, et al. Effects of Enriched Environment on COX-2, Leptin and Eicosanoids in a Mouse Model of Breast Cancer. PLoS ONE. 2012;7(12).
101. Naik RR, Sotnikov SV, Diepold RP, Iurato S, Markt PO, Bultmann A, et al. Polymorphism in Tmem132d regulates expression and anxiety-related behavior through binding of RNA polymerase II complex. Translational Psychiatry. 2018;8(1):1.
102. Narath E, Skalicky M, Viidik A. Voluntary and forced exercise influence the survival and body composition of ageing male rats differently. Experimental Gerontology. 2001;36(10):1699-711.
103. Nawaz A, Batool Z, Ahmed S, Khaliq S, Sajid I, Anis L, et al. Attenuation of Restraint Stress-Induced Behavioral Deficits by Environmental Enrichment in Male Rats. Pakistan Veterinary Journal. 2017;37(4).
104. Nguyen AP. Use of estrogen and rehabilitation to improve recovery in the collagenase model of intracerebral hemorrhage in rats [M.Sc.]. Ann Arbor: University of Alberta (Canada); 2008.
105. Nguyen AP, Arvanitidis AP, Colbourne F. Failure of estradiol to improve spontaneous or rehabilitation-facilitated recovery after hemorrhagic stroke in rats. Brain Research. 2008;1193:109-19.
106. Nicolas S, Veyssière J, Gandin C, Zsürger N, Pietri M, Heurteaux C, et al. Neurogenesis-independent antidepressant-like effects of enriched environment is dependent on adiponectin. Psychoneuroendocrinology. 2015;57:72-83.
107. Niwa A, Nishibori M, Hamasaki S, Kobori T, Liu K, Wake H, et al. Voluntary exercise induces neurogenesis in the hypothalamus and ependymal lining of the third ventricle. Brain Structure and Function. 2016;221(3):1653-66.
108. Novaes LS, Dos Santos NB, Batalhote RFP, Malta MB, Camarini R, Scavone C, et al. Environmental enrichment protects against stress-induced anxiety: Role of glucocorticoid receptor, ERK, and CREB signaling in the basolateral amygdala. Neuropharmacology. 2017;113(Pt A):457-66.

109. Nowakowska E, Kus K, Ratajczak P, Cichocki M, Woźniak A. The influence of aripiprazole, olanzapine and enriched environment on depressant-like behavior, spatial memory dysfunction and hippocampal level of BDNF in prenatally stressed rats. Pharmacological Reports. 2014;66(3):404-11.
110. Nygren J, Wieloch T. Enriched environment enhances recovery of motor function after focal ischemia in mice, and downregulates the transcription factor NGFI-A. Journal of Cerebral Blood Flow and Metabolism. 2005;25(12):1625-33.
111. Odeon MM, Acosta GB. Repeated maternal separation: Alcohol consumption, anxious behavior and corticosterone were reversed by a non-pharmacological treatment. Progress in Neuro-Psychopharmacology and Biological Psychiatry. 2019;95:109726.
112. Pagliusi M, Jr., Bonet IJM, Brandão AF, Magalhães SF, Tambeli CH, Parada CA, et al. Therapeutic and Preventive Effect of Voluntary Running Wheel Exercise on Social Defeat Stress (SDS)-induced Depressive-like Behavior and Chronic Pain in Mice. Neuroscience. 2020;428:165-77.
113. Park JH, Iemitsu M, Maeda S, Kitajima A, Nosaka T, Omi N. Voluntary running exercise attenuates the progression of endothelial dysfunction and arterial calcification in ovariectomized rats. Acta Physiologica. 2008;193(1):47-55.
114. Park JM, Seong HH, Jin HB, Kim YJ. The Effect of Long-Term Environmental Enrichment in Chronic Cerebral Hypoperfusion-Induced Memory Impairment in Rats. Biological Research for Nursing. 2017;19(3):278-86.
115. Parry TL, Hayward R. Exercise training does not affect anthracycline antitumor efficacy while attenuating cardiac dysfunction. American Journal of Physiology - Regulatory Integrative and Comparative Physiology. 2015;309(6):R675-R83.
116. Parry TL, Hayward R. Exercise Protects against Cancer-induced Cardiac Cachexia. Medicine and Science in Sports and Exercise. 2018;50(6):1169-76.
117. Patel DI, Abuchowski K, Sheikh B, Rivas P, Musi N, Kumar AP. Exercise preserves muscle mass and force in a prostate cancer mouse model. European Journal of Translational Myology. 2019;29(4):328-33.
118. Patel DI, Wallace D, Abuchowski K, Rivas P, Gallegos A, Musi N, et al. Nexrutine® preserves muscle mass similar to exercise in prostate cancer mouse model. Physiological Reports. 2019;7(16).
119. Pedersen L, Idorn M, Olofsson GH, Lauenborg B, Nookaew I, Hansen RH, et al. Voluntary running suppresses tumor growth through epinephrine- and IL-6-dependent NK cell mobilization and redistribution. Cell Metabolism. 2016;23(3):554-62.
120. Pedersen KS, Gatto F, Zerahn B, Nielsen J, Pedersen BK, Hojman P, et al. Exercise-Mediated Lowering of Glutamine Availability Suppresses Tumor Growth and Attenuates Muscle Wasting. iScience. 2020;23(4).
121. Pellegrin M, Aubert JF, Bouzourène K, Amstutz C, Mazzolai L. Voluntary exercise stabilizes established angiotensin II-dependent atherosclerosis in mice through systemic anti-inflammatory effects. PLoS ONE. 2015;10(11).
122. Pence BD, Ryerson MR, Cruz AGB, Woods JA, Shisler JL. Voluntary wheel running does not alter mortality to or immunogenicity of vaccinia virus in mice: A pilot study. Frontiers in Physiology. 2018;8(JAN).
123. Pierce AN, Eller-Smith OC, Christianson JA. Voluntary wheel running attenuates urinary bladder hypersensitivity and dysfunction following neonatal maternal separation in female mice. Neurourology and Urodynamics. 2018;37(5):1623-32.
124. Pigna E, Berardi E, Aulino P, Rizzuto E, Zampieri S, Carraro U, et al. Aerobic Exercise and Pharmacological Treatments Counteract Cachexia by Modulating Autophagy in Colon Cancer. Scientific Reports. 2016;6.
125. Ploeger JM, Manivel JC, Boatner LN, Mashek DG. Caloric restriction prevents carcinogen-initiated liver tumorigenesis in mice. Cancer Prevention Research. 2017;10(11):660-70.
126. Puurunen K, Jolkkonen J, Sirviö J, Haapalinna A, Sivenius J. An α2-adrenergic antagonist, atipamezole, facilitates behavioral recovery after focal cerebral ischemia in rats. Neuropharmacology. 2001;40(4):597-606.
127. Qian HZ, Zhang H, Yin LL, Zhang JJ. Postischemic Housing Environment on Cerebral Metabolism and Neuron Apoptosis after Focal Cerebral Ischemia in Rats. Current Medical Science. 2018;38(4):656-65.
128. Ravenelle R, Byrnes EM, Byrnes JJ, McInnis C, Park JH, Donaldson ST. Environmental enrichment effects on the neurobehavioral profile of selective outbred trait anxiety rats. Behav Brain Res. 2013;252:49-57.
129. Ravenelle R, Santolucito HB, Byrnes EM, Byrnes JJ, Donaldson ST. Housing environment modulates physiological and behavioral responses to anxiogenic stimuli in trait anxiety male rats. Neuroscience. 2014;270:76-87.
130. Reddy BS, Sugie S, Lowenfels A. Effect of voluntary exercise on azoxymethane-induced colon carcinogenesis in male f344 rats. Cancer Research. 1988;48:7079-81.
131. Reijne AC, Talarovicova A, Ciapaite J, Bruggink JE, Bleeker A, Groen AK, et al. Running wheel access fails to resolve impaired sustainable health in mice feeding a high fat sucrose diet. Aging. 2019;11(5):1564-79.
132. Rha DW, Kang SW, Park YG, Cho SR, Lee WT, Lee JE, et al. Effects of constraint-induced movement therapy on neurogenesis and functional recovery after early hypoxic-ischemic injury in mice. Developmental Medicine and Child Neurology. 2011;53(4):327-33.
133. Risedal A, Mattsson B, Dahlqvist P, Nordborg C, Olsson T, Johansson BB. Environmental influences on functional outcome after a cortical infarct in the rat. Brain Research Bulletin. 2002;58(3):315-21.

134. Rogers J, Vo U, Buret L, Pang T, Meiklejohn H, Zeleznikow-Johnston A, et al. Dissociating the therapeutic effects of environmental enrichment and exercise in a mouse model of anxiety with cognitive impairment. Translational psychiatry. 2016;6(4):e794-e.
135. Rogers J, Li S, Lanfumey L, Hannan AJ, Renoir T. Environmental enrichment reduces innate anxiety with no effect on depression-like behaviour in mice lacking the serotonin transporter. Behav Brain Res. 2017;332:355-61.
136. Rosenfeld A, Weller A. Behavioral effects of environmental enrichment during gestation in WKY and Wistar rats. Behavioural brain research. 2012;233(2):245-55.
137. Ruscher K, Shamloo M, Rickhag M, Ladunga I, Soriano L, Gisselsson L, et al. The sigma-1 receptor enhances brain plasticity and functional recovery after experimental stroke. Brain. 2011;134(Pt 3):732-46.
138. Rzechorzek W, Zhang H, Buckley BK, Hua K, Pomp D, Faber JE. Aerobic exercise prevents rarefaction of pial collaterals and increased stroke severity that occur with aging. Journal of Cerebral Blood Flow and Metabolism. 2017;37(11):3544-55.
139. Sadeghi M, Peeri M, Hosseini MJ. Adolescent voluntary exercise attenuated hippocampal innate immunity responses and depressive-like behaviors following maternal separation stress in male rats. Physiol Behav. 2016;163:177-83.
140. Sah A, Sotnikov S, Kharitonova M, Schmuckermair C, Diepold RP, Landgraf R, et al. Epigenetic Mechanisms Within the Cingulate Cortex Regulate Innate Anxiety-Like Behavior. The international journal of neuropsychopharmacology. 2019;22(4):317-28.
141. Sahafi E, Peeri M, Hosseini MJ, Azarbayjani MA. Cardiac oxidative stress following maternal separation stress was mitigated following adolescent voluntary exercise in adult male rat. Physiol Behav. 2018;183:39-45.
142. Sato C, Tanji K, Shimoyama S, Chiba M, Mikami M, Koeda S, et al. Effects of voluntary and forced exercises on motor function recovery in intracerebral hemorrhage rats. NeuroReport. 2020:189-96.
143. Saucier DM, Yager JY, Armstrong EA, Keller A, Shultz S. Enriched environment and the effect of age on ischemic brain damage. Brain Research. 2007;1170(SUPPL.: COMPLETE):31-8.
144. Saucier DM, Yager JY, Armstrong EA. Housing environment and sex affect behavioral recovery from ischemic brain damage. Behavioural Brain Research. 2010;214(1):48-54.
145. Schloesser RJ, Lehmann M, Martinowich K, Manji HK, Herkenham M. Environmental enrichment requires adult neurogenesis to facilitate the recovery from psychosocial stress. Molecular psychiatry. 2010;15(12):1152-63.
146. Shing CM, Fassett RG, Peake JM, Coombes JS. Voluntary exercise decreases atherosclerosis in nephrectomised ApoE knockout mice. PLoS ONE. 2015;10(3).
147. Shtoots L, Richter-Levin G, Hugeri O, Anunu R. Juvenile stress leads to long-term immunological metaplasticity-like effects on inflammatory responses in adulthood. Neurobiol Learn Mem. 2018;154:12-21.
148. Sierakowiak A, Mattsson A, Gomez-Galan M, Feminia T, Graae L, Aski SN, et al. Hippocampal morphology in a rat model of depression: the effects of physical activity. The Open Neuroimaging Journal. 2014;9:1-6.
149. Skillings EA, Wood NI, Morton AJ. Beneficial effects of environmental enrichment and food entrainment in the R6/2 mouse model of Huntington's disease. Brain and Behavior. 2014;4(5):675-86.
150. Smeda M, Przyborowski K, Proniewski B, Zakrzewska A, Kaczor D, Stojak M, et al. Breast cancer pulmonary metastasis is increased in mice undertaking spontaneous physical training in the running wheel; a call for revising beneficial effects of exercise on cancer progression. American Journal of Cancer Research. 2017;7(9):1926-36.
151. Solberg LC, Horton TH, Turek FW. Circadian rhythms and depression: effects of exercise in an animal model. The American journal of physiology. 1999;276(1):R152-61.
152. Song Y, Gan Y, Wang Q, Meng Z, Li G, Shen Y, et al. Enriching the Housing Environment for Mice Enhances Their NK Cell Antitumor Immunity via Sympathetic Nerve-Dependent Regulation of NKG2D and CCR5. Cancer Research. 2017;77(7):1611-22.
153. Steiner JL, Davis JM, McClellan JL, Enos RT, Murphy EA. Effects of voluntary exercise on tumorigenesis in the C3(1)/SV40Tag transgenic mouse model of breast cancer. International Journal of Oncology. 2013;42(4):1466-72.
154. Strong PV, Greenwood BN, Fleshner M. The effects of the selective 5-HT(2C) receptor antagonist SB 242084 on learned helplessness in male Fischer 344 rats. Psychopharmacology (Berl). 2009;203(4):665-75.
155. Su Q, Pu H, Hu C. Neuroprotection by combination of resveratrol and enriched environment against ischemic brain injury in rats. Neurological Research. 2016;38(1):60-8.
156. Sugie S, Reddy BS, Lowenfels A, Tanaka T, Mori H. Effect of voluntary exercise on azoxymethane-induced hepatocarcinogenesis in male F344 rats. Cancer Letters. 1992;63(1):67-72.
157. Tang Q, Yang Q, Hu Z, Liu B, Shuai J, Wang G, et al. The effects of willed movement therapy on AMPA receptor properties for adult rat following focal cerebral ischemia. Behavioural Brain Research. 2007;181(2):254-61.
158. Tang Y, Li MY, Zhang X, Jin X, Liu J, Wei PH. Delayed exposure to environmental enrichment improves functional outcome after stroke. Journal of Pharmacological Sciences. 2019;140(2):137-43.
159. Thompson HJ, Wolfe P, McTiernan A, Jiang W, Zhu Z. Wheel running-induced changes in plasma biomarkers and carcinogenic response in the 1-methyl-1-nitrosourea-induced rat model for breast cancer. Cancer Prevention Research. 2010;3(11):1484-92.
160. Vaanholt LM, Daan S, Garland Jr T, Visser GH. Exercising for life? Energy metabolism, body composition, and longevity in mice exercising at different intensities. Physiological and Biochemical Zoology. 2010;83(2):239-51.

161. Vahid-Ansari F, Albert PR. Chronic Fluoxetine Induces Activity Changes in Recovery From Poststroke Anxiety, Depression, and Cognitive Impairment. Neurotherapeutics. 2018;15(1):200-15.
162. van Deel ED, de Boer M, Kuster DW, Boontje NM, Holemans P, Sipido KR, et al. Exercise training does not improve cardiac function in compensated or decompensated left ventricular hypertrophy induced by aortic stenosis. Journal of Molecular and Cellular Cardiology. 2011;50(6):1017-25.
163. Vega-Rivera NM, Ortiz-López L, Gómez-Sánchez A, Oikawa-Sala J, Estrada-Camarena EM, Ramírez-Rodríguez GB. The neurogenic effects of an enriched environment and its protection against the behavioral consequences of chronic mild stress persistent after enrichment cessation in six-month-old female Balb/C mice. Behavioural Brain Research. 2016;301:72-83.
164. Wang X, Chen A, Wu H, Ye M, Cheng H, Jiang X, et al. Enriched environment improves post-stroke cognitive impairment in mice by potential regulation of acetylation homeostasis in cholinergic circuits. Brain Research. 2016;1650:232-42.
165. Wang C, Zhang Q, Yu K, Shen X, Wu Y, Wu J. Enriched Environment Promoted Cognitive Function via Bilateral Synaptic Remodeling After Cerebral Ischemia. Frontiers in neurology [electronic resource]. 2019;10:1189.
166. Wang J, Truong T, Ladiges W, Goh J. Rapamycin increases breast tumor burden in young wheel-running mice. Pathobiology of Aging & Age Related Diseases. 2019;9(1):1647746.
167. Watanabe J, Kagami N, Kawazoe M, Arata S. A simplified enriched environment increases body temperature and suppresses cancer progression in mice. Experimental Animals. 2020;69(2):207-18.
168. Welsch MA, Cohen LA, Welsch CW. INHIBITION OF GROWTH OF HUMAN BREAST-CARCINOMA XENOGRAFTS BY ENERGY-EXPENDITURE VIA VOLUNTARY EXERCISE IN ATHYMIC MICE FED A HIGH-FAT DIET. Nutrition and Cancer-an International Journal. 1995;23(3):309-18.
169. Wolff G, Davidson SJ, Wrobel JK, Toborek M. Exercise maintains blood-brain barrier integrity during early stages of brain metastasis formation. Biochemical and Biophysical Research Communications. 2015;463(4):811-7.
170. Wu Y, Gan Y, Yuan H, Wang Q, Fan Y, Li G, et al. Enriched environment housing enhances the sensitivity of mouse pancreatic cancer to chemotherapeutic agents. Biochemical and Biophysical Research Communications. 2016;473(2):593-9.
171. Wurm F, Keiner S, Kunze A, Witte OW, Redecker C. Effects of skilled forelimb training on hippocampal neurogenesis and spatial learning after focal cortical infarcts in the adult rat brain. Stroke. 2007;38(10):2833-40.
172. Xiao R, Bergin SM, Huang W, Slater AM, Liu X, Judd RT, et al. Environmental and Genetic Activation of Hypothalamic BDNF Modulates T-cell Immunity to Exert an Anticancer Phenotype. Cancer Immunology Research. 2016;4(6):488-97.
173. Yan L, Demars LC. Effects of non-motorized voluntary running on experimental and spontaneous metastasis in mice. Anticancer Research. 2011;31(10):3337-44.
174. Yu K, Wu Y, Hu Y, Zhang Q, Xie H, Liu G, et al. Neuroprotective effects of prior exposure to enriched environment on cerebral ischemia/reperfusion injury in rats: the possible molecular mechanism. Brain Research. 2013;1538:93-103.
175. Yu K, Wu Y, Zhang Q, Xie H, Liu G, Guo Z, et al. Enriched environment induces angiogenesis and improves neural function outcomes in rat stroke model. Journal of the Neurological Sciences. 2014;347(1-2):275-80.
176. Yu KW, Wang CJ, Wu Y, Wang YY, Wang NH, Kuang SY, et al. An enriched environment increases the expression of fibronectin type III domain-containing protein 5 and brain-derived neurotrophic factor in the cerebral cortex of the ischemic mouse brain. Neural Regeneration Research. 2020;15(9):1671-7.
177. Zhang X, Chen XP, Lin JB, Xiong Y, Liao WJ, Wan Q. Effect of enriched environment on angiogenesis and neurological functions in rats with focal cerebral ischemia. Brain Research. 2017;1655:176-85.
178. Zhang Z, Li R, Zhang X, Wei Y, Ma H, Zhu L, et al. Voluntary exercise promotes neurotrophic factor and suppresses apoptosis in hippocampal ischemia. Journal of integrative neuroscience. 2019;18(1):65-70.
179. Zhang Q, Wu JF, Shi QL, Li MY, Wang CJ, Wang X, et al. The Neuronal Activation of Deep Cerebellar Nuclei Is Essential for Environmental Enrichment-Induced Post-Stroke Motor Recovery. Aging & Disease. 2019;10(3):530-43.
180. Zheng H, Liu Y, Li W, Yang B, Chen D, Wang X, et al. Beneficial effects of exercise and its molecular mechanisms on depression in rats. Behav Brain Res. 2006;168(1):47-55.
181. Zheng X, Cui XX, Huang MT, Liu Y, Shih WJ, Lin Y, et al. Inhibitory effect of voluntary running wheel exercise on the growth of human pancreatic Panc-1 and prostate PC-3 xenograft tumors in immunodeficient mice. Oncology Reports. 2008;19(6):1583-8.
182. Zheng X, Cui XX, Gao Z, Zhao Y, Shi Y, Huang MT, et al. Inhibitory effect of dietary atorvastatin and celecoxib together with voluntary running wheel exercise on the progression of androgen-dependent LNCaP prostate tumors to androgen independence. Experimental and Therapeutic Medicine. 2011;2(2):221-8.
183. Zheng X, Cui XX, Huang MT, Liu Y, Wagner GC, Lin Y, et al. Inhibition of progression of androgen-dependent prostate LNCaP tumors to androgen independence in SCID mice by oral caffeine and voluntary exercise. Nutrition and Cancer. 2012;64(7):1029-37.
184. Zhu ZJ, Jiang WQ, Sells JL, Neil ES, McGinley JN, Thompson HJ. Effect of nonmotorized wheel running on mammary carcinogenesis: circulating biomarkers, cellular processes, and molecular mechanisms in rats. Cancer Epidemiology, Biomarkers & Prevention. 2008;17(8):1920-9.
185. Zhu Z, Jiang W, McGinley JN, Thompson HJ. Energetics and mammary carcinogenesis: Effects of moderate-intensity running and energy intake on cellular processes and molecular mechanisms in rats. Journal of Applied Physiology. 2009;106(3):911-8.

186. Zhu H. An enriched environment reverses the cognitive impairment and synaptic plasticity deficit induced by chronic cerebral hypoperfusion [Ph.D.]. Ann Arbor: Wuhan University (People's Republic of China); 2011.
