# Supplementary figures and images for: Conventional laboratory housing increases morbidity and mortality in research rodents: results of a meta-analysis
Source: BMC Biol. 2022 Jan 13;20:15. doi: 10.1186/s12915-021-01184-0 (PMC8756709; doi:10.1186/s12915-021-01184-0)

**A**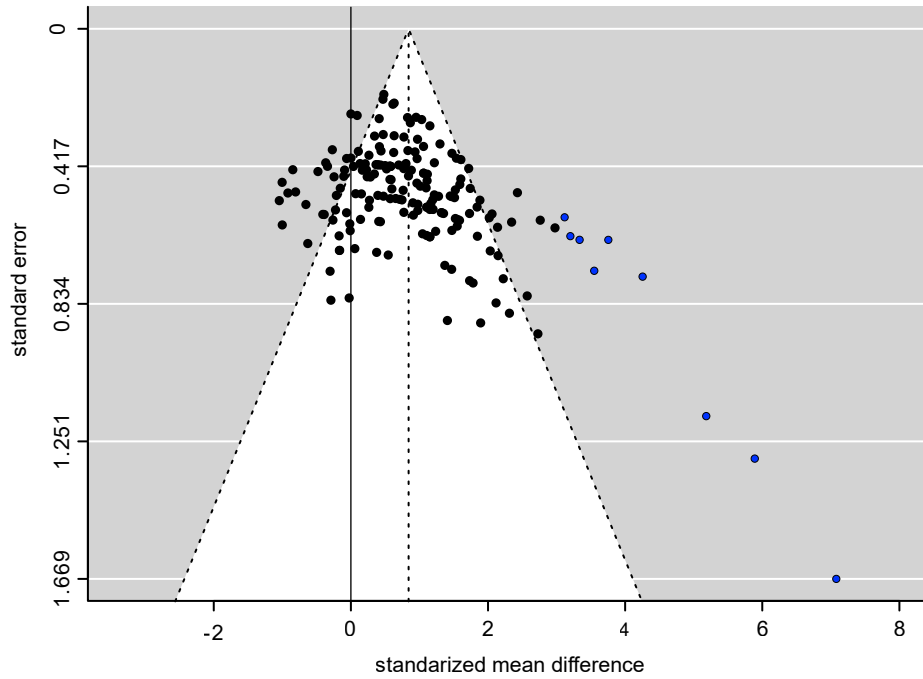**B**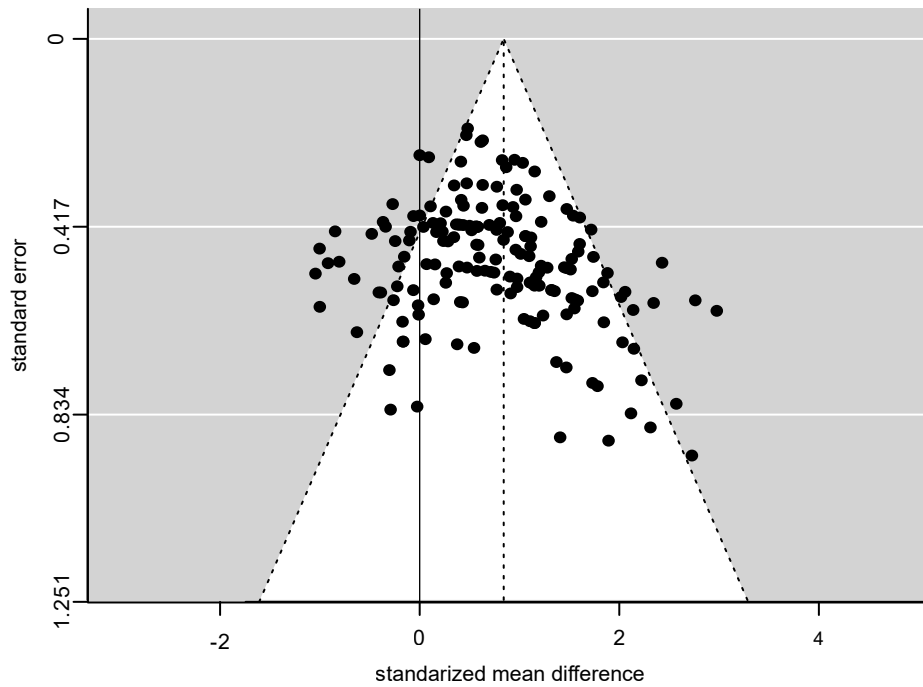

Supplement: Supplementary file 18 — Additional file 18. Funnel plot for all stress-sensitive diseases. (A) Initial funnel plot including all stress-sensitive disease data. Blue dots indicate studies contributing to plot asymmetry (B) Funnel plot of studies included in final analysis with the publication bias removed. Bold line = null result, dotted line = standardized mean estimate calculated from all included studies. A plot with no publication bias should look symmetrical around the dotted line. [file 12915_2021_1184_MOESM18_ESM.pdf]

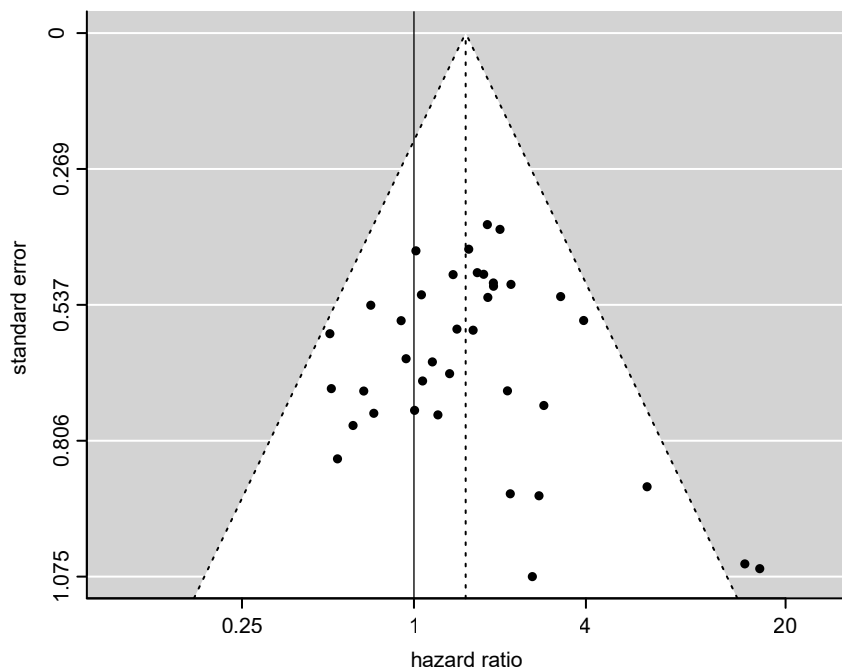

Supplement: Supplementary file 19 — Additional file 19. A funnel plot of all studies included in the analysis reporting hazard ratios. Bold line = null result, dotted line = hazard ratio estimate calculated from all included studies. [file 12915_2021_1184_MOESM19_ESM.pdf]

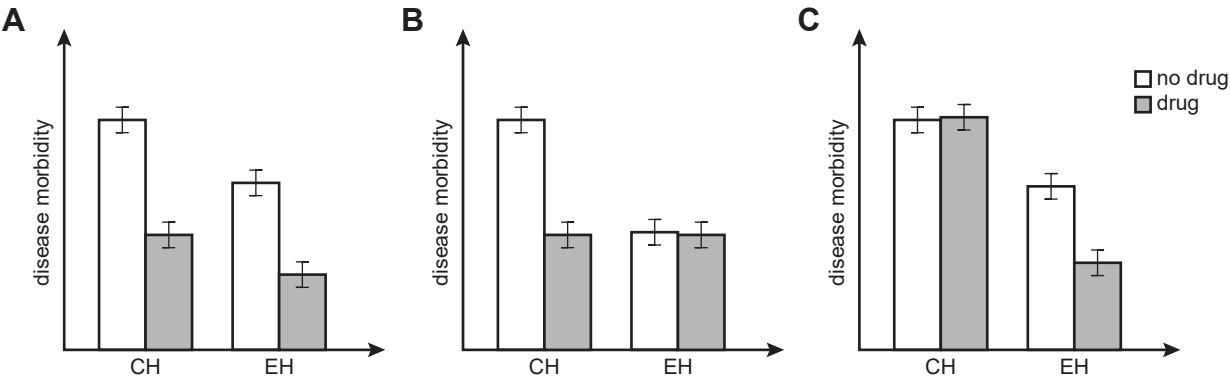

Supplement: Supplementary file 21 — Additional file 21. Hypothetical data illustrating how ‘enrichments’ could impact external validity. This figure demonstrates how interactive effects between a treatment (e.g. a drug) and EH could impact experimental conclusions. Note that in each graph the error bars do not change, as EH does not change data variability. Also note that EH is not one unitary thing, but something that can vary in kind and degree (for example to deliberately introduce heterogeneity). (A) No interactive effect. No matter the cage condition, the experimental conclusion is the same: the drug reduces disease but does not cure it. EH does not affect external validity. (B) The drug effect is absent with EH. This drug could be useful for specific populations (e.g. chronically stressed and/or overweight subjects) but not others (e.g. physically fit content subjects). Testing the drug only under CH conditions will generate false positives, unless the target population is specifically one which is stressed and/or overweight etc. (C) The drug effect is only detectable in EH. This suggests the drug could be useful for some populations (e.g. ones which are physically fit and content) but not others (e.g. chronically stressed and/or overweight) Testing the drug only under CH conditions will generate false negatives, unless the target population is specifically one which is stressed and/or overweight. [file 12915_2021_1184_MOESM21_ESM.pdf]
