# Supplementary material for: Conventional laboratory housing increases morbidity and mortality in research rodents: results of a meta-analysis
Source: BMC Biol. 2022 Jan 13;20:15. doi: 10.1186/s12915-021-01184-0 (PMC8756709; doi:10.1186/s12915-021-01184-0)
Supplement: Supplementary file 20 — Additional file 20. GRADE assessment of confidence of cumulative evidence. SMD = standardized mean difference. [file 12915_2021_1184_MOESM20_ESM.pdf]

**No. of studies**	**Risk of bias in individual studies**	**Inconsistency**	**Indirectness**	**Imprecision**	**Publication bias**	**Housing effect result summary**	**Quality**
mortality data (25)	serious risk of bias	no serious inconsistency	no serious indirectness	no serious imprecision	undetected	hazard ratio = 1.48<br>95%CI = 1.25-1.74<br>$p < 0.0001$	High
disease data (157)	serious risk of bias	no serious inconsistency	no serious indirectness	no serious imprecision	detected	SMD = 0.73<br>95%CI = 0.63-0.84<br>$p < 0.0001$	High
